# Expression and prognostic analyses of the insulin-like growth factor 2 mRNA binding protein family in human pancreatic cancer

**DOI:** 10.1186/s12885-020-07590-x

**Published:** 2020-11-27

**Authors:** Xiao-Han Cui, Shu-Yi Hu, Chun-Fu Zhu, Xi-Hu Qin

**Affiliations:** 1 Department of General Surgery, the Affiliated Changzhou No. 2 People’s Hospital of Nanjing Medical University, 68 Pohu Middle Road, Changzhou, Jiangsu 213000 P.R. China; 2 Nanjing Medical University, Nanjing, Jiangsu 211166 P.R. China; 3 Department of General Surgery, Liver Transplantation Center, The First Affiliated Hospital of Nanjing Medical University, Nanjing, Jiangsu P.R. China

**Keywords:** Pancreatic Cancer, Expression, Prognosis, IGF2BP, Bioinformatic

## Abstract

**Background:**

Despite advances in early diagnosis and treatment, cancer remains the leading cause of mortality worldwide. The insulin-like growth factor 2 mRNA binding protein (IGF2BP) family has been reported to be involved in a variety of human malignant tumours. However, little is known about their expression and prognostic value in human pancreatic cancer. Therefore, we performed a detailed cancer versus normal differential analysis.

**Methods:**

The Cancer Genome Atlas (TCGA) and Gene Expression Profiling Interactive Analysis (GEPIA) databases were used to analyse the mRNA expression levels of the IGF2BP family in various cancers, including pancreatic cancer. Then, the LinkedOmics and GEPIA databases were used to assess the relation between the expression levels of IGF2BPs and overall survival (OS). Then, univariate and multivariate Cox regression analyses were performed, and subgroups based on grade and stage were analysed. The signalling pathways associated with IGF2BP2 and IGF2BP3 were then investigated via gene set enrichment analysis (GSEA).

**Results:**

IGF2BP2 and IGF2BP3 were associated with each subset of OS based on grade and stage. Further clinical correlation analysis of IGF2BP2 and IGF2BP3 confirmed that IGF2BP2 and IGF2BP3 are fundamental factors in promoting pancreatic cancer progression.

**Conclusion:**

IGF2BP2 and IGF2BP3 are key factors in promoting the progression of pancreatic cancer and are closely related to overall survival.

## Background

Pancreatic cancer is a high-mortality tumour with a five-year overall survival rate of approximately 7% [1, 2]. Among the causes of cancer-related death, this malignant tumour ranks fourth in the United States and sixth in China [1, 3]. Approximately 80% of patients with pancreatic cancer have dissemination at the time of diagnosis [1, 4]. These patients have lost the chance for radical treatment of pancreatic cancer. In the past decade, despite advancements in anti-metabolism therapy and targeted therapy, the overall survival rate of patients has not significantly improved due to the late pathological stage, high invasive phenotype and chemotherapy resistance.

Insulin-like growth factor 2-mRNA binding proteins (IGF2BPs), also known as IGF-II mRNA binding proteins (IMPs), are encoded by different genes that belong to the regulatory RNA binding protein family and are involved in the localization of their target RNA, stability and translation control [5]. As the names of these proteins indicate, they are recognized members of the IGF axis that can be linked to IGF2 transcripts [6, 7]. To date, insulin-like growth factor 2 mRNA binding proteins, including IGF2BP1 (IMP1), IGF2BP2 (IMP2), and IGF2BP3 (IMP3), are a unique family of m6A readers that target the common m6A sequence by recognizing thousands of mRNA transcripts [8]. In mammals, the protein domains of the three members of the IGF2BP protein family are strikingly similar. All three members of the protein family contain two N-terminal RRMs and four C-terminal hnRNPK homology (KH) domains. The latter are arranged in two dual domains (KH1 + 2 and KH3 + 4) [9]. Consistent with the conservation of six potential RNA binding domains, all three IGF2BPs bind to single-stranded RNA in vitro and in vivo [9–11]. However, the role of the entire IGF2BP family in pancreatic cancer remains controversial. Therefore, it was necessary to probe the role of the IGF2BP family in pancreatic cancer.

The Cancer Genome Atlas (TCGA) is considered to be the largest cancer database, containing more than 20,000 primary cancer samples and normal matched samples for multiple cancer types. Therefore, we can use bioinformatics methods to study tumour data more deeply. To evaluate the relationship between the IGF2BP family and pancreatic cancer progression, we analysed mRNA expression in pancreatic cancer samples from the TCGA with R software and verified it in patients.

## Methods

### GEPIA dataset

Gene Expression Profiling Interactive Analysis (GEPIA) is a new web-based tool for gene expression analysis between tumour and normal data from The Cancer Genome Atlas (TCGA) and the Genotype-Tissue Expression (GTEx) project, applying a standard processing pipeline. It provides customizable functions such as tumour and normal differential expression analysis, and we can demonstrate the expression of IGF2BP1–3 in pancreatic cancer and normal tissues. GEPIA possesses key variable and interactive functions, including profile plotting, differential expression analysis, patient survival analysis, similar gene detection and dimensionality reduction analysis.

### LinkedOmics dataset

LinkedOmics is a new and unique tool in the software ecosystem for disseminating data from all 32 TCGA cancer types. It can be used to access, analyse, and compare multiomics data within and across tumour types. We performed a prognostic analysis for the IGF2BP gene family using the LinkedOmics pancreatic cancer dataset.

### TCGA data acquisition and differentially expressed IGF2BP gene analysis

The pancreatic cancer data in the TCGA contains 178 pancreatic cancer samples with important information, including pathological grade and clinical stage. All mRNA expression data, along with clinical data, were downloaded and further analysed with R software.

We utilized the “limma” package in R software to normalize the original expression levels of mRNAs downloaded from the TCGA. The “limma” package was used to analyse the expression of each IGF2BP gene between every grade and stage of cancer tissues. Last, a *P*-value < 0.05 was set as the filter condition for differentially expressed IGF2BP.

### Gene set enrichment analysis of pancreatic cancer

Performed gene enrichment analysis (version 3.0, the broad Institute of MIT and Harvard, http://software.broadinstitute.org/gsea/downloads.jsp) between pancreatic cancer and normal tissues to study the biological pathways of pancreatic cancer. Specifically, set “collapse data set to gene symbols” to false, set the number of marks to 1000, set the “permutation type” to phenotype, set the “enrichment statistic” to weighted, and utilized the Signal2Noise metric to rank genes. The high expression group was taken as the experimental group, and the low expression group was taken as the reference group. The “c2.cp.kegg.v7.0.symbols.gmt” gene set database was utilized for enrichment analysis. Cut-off criteria including gene set size > 500 and < 15, FDR < 0.25, and nominal *P*-value < 0.05.

### Functional enrichment analyses of pancreatic cancer

Kyoto Encyclopedia of Genes and Genomes (KEGG) and Gene Ontology (GO) functional enrichment analyses were performed to analyse IGF2BP2 and IGF2BP3. The Database for Annotation, Visualization, and Integrated Discovery (DAVID, https://david.ncifcrf.gov/) was applied to identify enriched KEGG and GO pathways and terms.

### Cell lines and reagents

The human pancreatic cancer cell lines, including ASPC-1, SW1990, PANC-1, MIA Paca-2 and HPDE6-C7, were purchased from the University of Colorado Cancer Center Cell Bank. ASPC-1, PANC-1, MIA Paca-2 and HPDE6-C7 cells were cultured in DMEM medium with 10% FBS (Invitrogen, Carlsbad, CA, USA) at 37 °C in a 5% CO_2_ atmosphere. SW1990 cells were cultured in RPMI 1640 medium supplemented with 10% FBS (Invitrogen, Carlsbad, CA, USA) at 37 °C in a 5% CO_2_ atmosphere. Cells were digested and passaged when cell confluence reached 80–90%.

### Quantitative reverse transcription polymerase chain reaction

Total RNA was extracted from the ASPC-1, SW1990, PANC-1, MIA Paca-2 and HPDE6-C7 cell lines using TRIzol reagent (Life Technologies) according to the instructions provided by the manufacturer. Total RNA (1 μg) was used as a template to synthesize complementary DNA (cDNA) using a PrimeScript RT Reagent Kit with cDNA Eraser (Takara Biotechnology). Subsequently, qRT-PCR was performed using SYBR Premix Ex Taq (Takara Bio Inc.). The primer sequences used for real-time PCR are listed in Table S1. All qRT-PCR assays were performed on an ABI 7900 system (Applied Biosystems).

### Cell proliferation assay

The Cell Counting Kit-8 (CCK-8) assay (MedChemExpress) was used according to the protocol provided by the manufacturer to assess cell proliferation. ASPC-1 and SW1990 cells were seeded into 96-well plates (5 × 10^3^ cells/plate) and cultured at 37 °C. Ten microlitres of CCK-8 solution was added to each well of the plate at the following times: 0 h, 24 h, 48 h, 72 h and 96 h. Optical density (OD) was measured at 1–4 days at a wavelength of 450 nm using a Multiskan FC microplate reader (Thermo Fisher Scientific, Inc.).

### Protein extraction and Western blot analysis

Utilized RIPA lysis buffer with 1% phenylmethanesulfonyl fluoride (PMSF) and DL-dithiothreitol (DTT) to extracted total protein. The BCA protein assay kit (Beyotime Biotechnology) was used to determine the concentration of the protein lysate. Equivalent (30 μg) protein was isolated by 10% SDS-PAGE. Then, the proteins were transferred to PVDF membranes (0.45 mm; Beijing Solarbio Science & Technology Co., China). Before incubated the membranes with IGF2BP2 and IGF2BP3 antibodies (1:1000, R&D Systems, MN, USA) at 4 °C for 12 h, the membranes were blocked at room temperature with 5% BSA for 1 h. Then GAPDH rabbit polyclonal antibody (1:4000, Proteintech, USA) was utilized as a loading control for normalization. HRP-conjugated secondary anti-rabbit antibody (1:4000; ProteinTech Group) was incubated at room temperature about 1 h. Finally, the bands were placed on an Omega Lum G machine (Aplegen, USA) and visualized using ECL reagents (Thermo Fisher Scientific).

### Colony formation assay

SW1990 cells were seeded into 6-well plates (1 × 10^3^ cells/plate) and cultured for 14 days. Then, cells were fixed with 10% formaldehyde for 5 min and stained with 1% crystal violet for 30 s prior to counting the number of colonies.

### Cell invasion assay

Cell invasion was performed with transwell plates (24-well insert, 8 μm pore size; BD Biosciences, Bedford, MA, USA). The filters (Corning Inc., USA) were covered with 55 μL of Matrigel (1:8 dilution; BD Biosciences). Then, 5 × 10^4^ SW1990 cells were distributed in 100 μl of serum-free RPMI-1640 medium and inoculated into the upper chamber. Next, 600 μl of 90% RPMI-1640 medium supplemented with 10% FBS was added to the bottom chamber. After 24 h of incubation, the chamber was fixed with 4% paraformaldehyde for 30 min and then stained with 0.1% crystal violet for 30 min. Finally, magnification microscope to count the number of invading cells in the bottom chamber.

### Statistical analysis

In this study, the experiments were carried out in triplicate, and the data were expressed as the mean ± standard deviation. The t-test was utilized for the statistical analysis of the data. Comparisons between multiple groups were performed with one-way ANOVA followed by an LSD-t test. *P* < 0.05 was considered significant.

## Results

### Transcriptional levels of IGF2BPs in patients with pancreatic Cancer

**Fig. 1:**
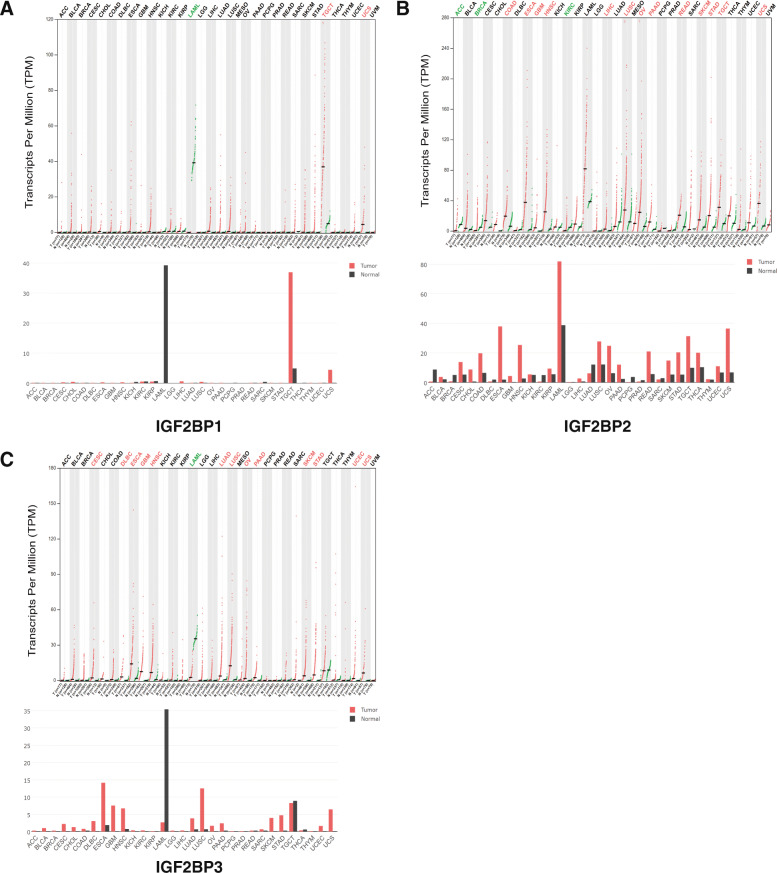
The Transcription Levels of IGF2BP Factors in Different Types of Cancer (GEPIA). **a**-**c** The expression of IGF2BPs in pan-cancer

Three IGF2BP factors were identified in mammalian cells, and the expression levels of the IGF2BPs in various cancers were compared via the GEPIA database. The IGF2BP1 mRNA expression level was not upregulated in pancreatic cancer, but the IGF2BP2 and IGF2BP3 mRNA expression levels were upregulated in pancreatic cancer to varying degrees (Fig. 1).

The GEPIA database was utilized to further analyse whether there was a difference in the expression of IGF2BP factors between pancreatic cancer and normal pancreatic tissues. According to ONCOMINE, the expression of IGF2BP1 in pancreatic cancer tissue was not significantly different from that in normal pancreatic tissue. However, in the pancreatic cancer datasets described by Segara, Pei, and Badea, IGF2BP2 was overexpressed in pancreatic carcinoma tissue compared with normal tissue, with fold changes of 3.446, 2.657, and 2.01, respectively (Table 1) [12–14]. Regarding IGF2BP3, all four pancreatic cancer datasets indicated overexpression in both pancreatic carcinoma and pancreatic ductal adenocarcinoma (Table 1) [12–16].

**Table 1:** The Significant Changes of IGF2BP Expression in Transcription Level between Different Types of Pancreatic Cancer (ONCOMINE Database)

Gene ID	Types of Pancreatic Cancer versus Norma	Fold Change	*p* Value	t Test	References
IGF2BP2	Pancreatic Carcinoma versus Normal	3.446	1.90E-06	7.957	Segara Pancreas Statistics [12]
	Pancreatic Carcinoma versus Normal	2.657	1.93E-07	6.193	Pei Pancreas Statistics [13]
	Pancreatic Ductal Adenocarcinoma versus Normal	2.01	5.63E-09	6.407	Badea Pancreas Statistics [14]
IGF2BP3	Pancreatic Carcinoma versus Normal	9.327	4.11E-12	9.295	Pei Pancreas Statistics [13]
	Pancreatic Ductal Adenocarcinoma versus Normal	3.528	1.61E-08	6.643	Badea Pancreas Statistics [14]
	Pancreatic Ductal Adenocarcinoma versus Normal	2.355	0.005	2.725	Ishikawa Pancreas Statistics [15]
	Pancreatic Ductal Adenocarcinoma Epithelia versus Normal	5.606	0.007	2.703	Grutzmann Pancreas Statistics [16]

### Expression levels of IGF2BPs in normal and pancreatic Cancer tissues

**Fig. 2:**
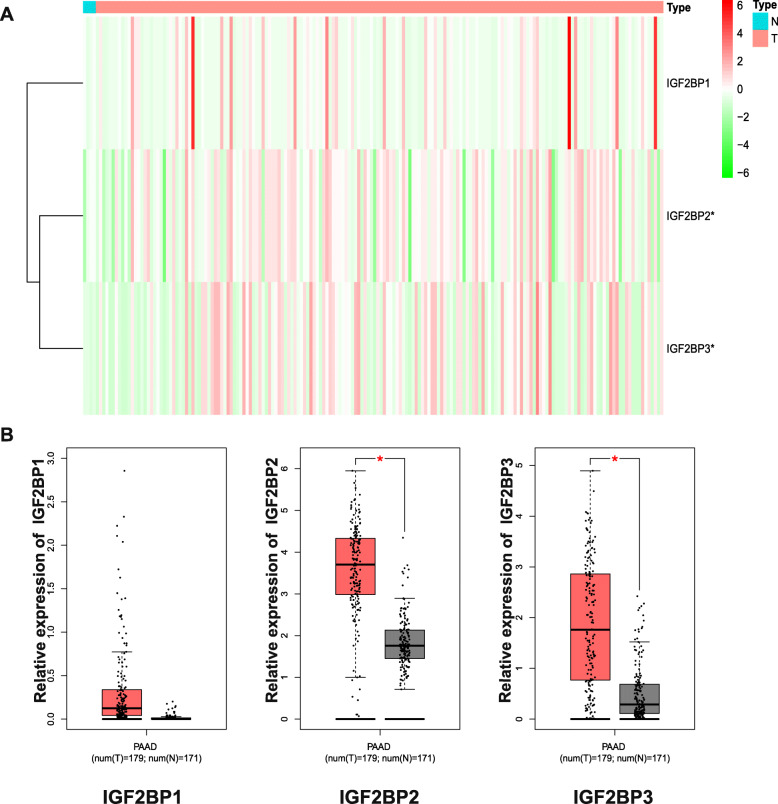
The Expression of IGF2BPs in Pancreatic Cancer (TCGA and GEPIA). **a** The expression of IGF2BPs in pancreatic cancer analysed with the TCGA. **b** The expression of IGF2BPs in pancreatic cancer analysed with GEPIA. **a**, **b** ^*^P < 0.05

We utilized the TCGA database to compare the expression levels of the IGF2BP family in normal pancreatic and pancreatic cancer tissues. Among the family members, the expression levels of IGF2BP2 and IGF2BP3 were significantly increased in pancreatic cancer tissues (Fig. 2a). With the GEPIA (Gene Expression Profiling Interactive Analysis) dataset (http://gepia.cancer-pku.cn/), we compared the mRNA expression levels of the members of the IGF2BP protein family between pancreatic cancer and normal tissues. The results showed that the expression of the IGF2BP1 gene in pancreatic cancer tissue was not different from that in normal pancreatic tissue. However, the expression levels of IGF2BP2 and IGF2BP3 were higher in pancreatic cancer tissue than in normal pancreatic tissue (Fig. 2b).

### Clinical correlation analysis in pancreatic Cancer patients

**Fig. 3:**
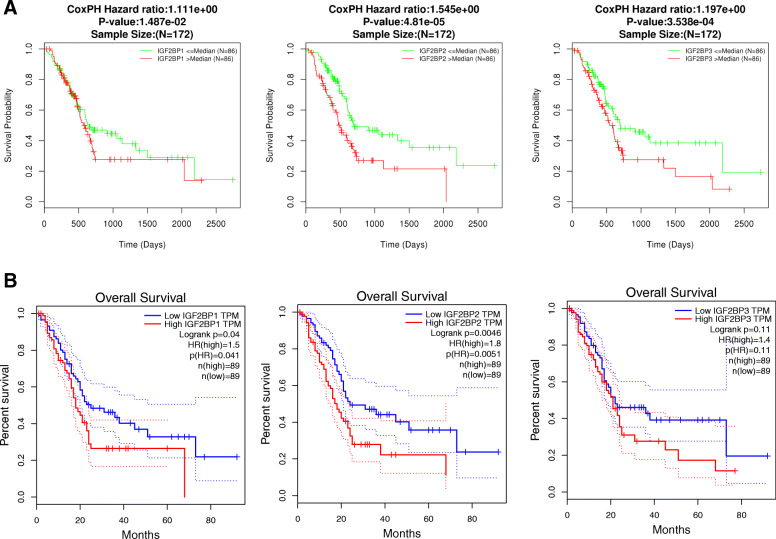
The Prognostic Value of the mRNA Levels of IGF2BP Factors in Pancreatic Cancer Patients (LinkedOmics and GEPIA). **a** The prognostic value of the mRNA levels of IGF2BP factors in pancreatic cancer patients analysed with LinkedOmics. **b** The prognostic value of the mRNA levels of IGF2BP factors in pancreatic cancer patients analysed with GEPIA

Furthermore, we performed a prognostic analysis of IGF2BP1, IGF2BP2, and IGF2BP3 in pancreatic cancer with the LinkedOmics and GEPIA datasets. In the LinkedOmics dataset, the high expression of IGF2BP1, IGF2BP2, and IGF2BP3 was significantly associated with the poor overall survival of pancreatic cancer patients (Fig. 3a). Interestingly, regarding IGF2BP1 and IGF2BP2, consistent results were obtained from the prognostic analysis of the GEPIA dataset (Fig. 3b).

**Fig. 4:**
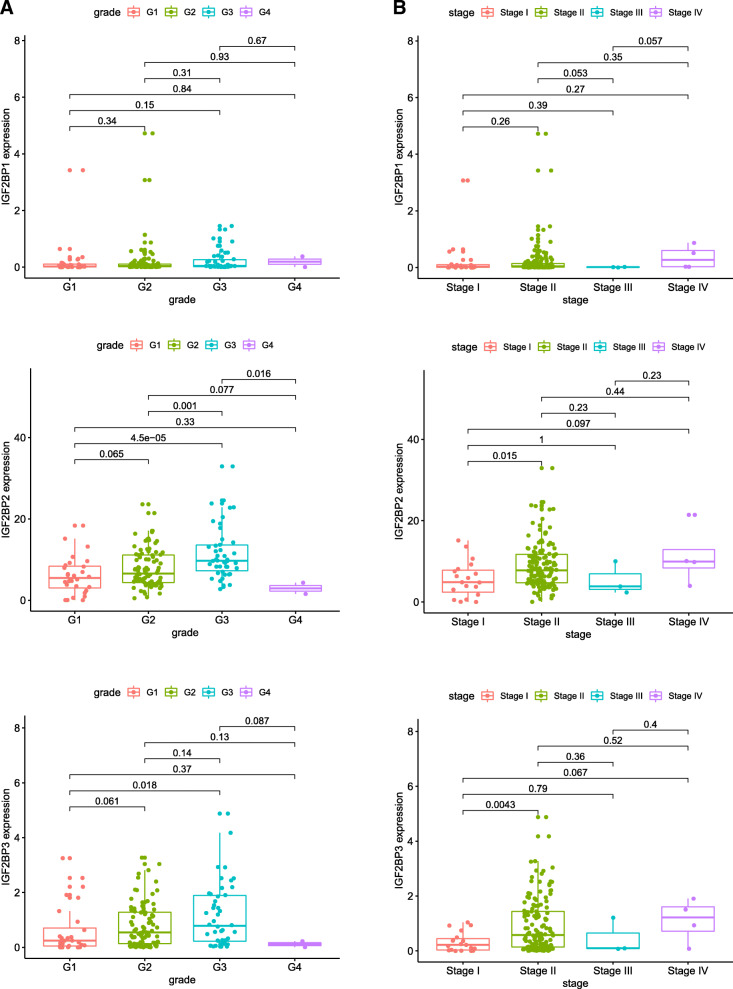
Correlation analysis between grade and stage and IGF2BP1–3 expression in 178 pancreatic cancer samples. **a** Correlation analysis between pathological grade and IGF2BP1–3 expression in 178 pancreatic cancer samples. **b** Correlation analysis between clinical stage and IGF2BP1–3 expression in 178 pancreatic cancer samples

The association between IGF2BP1–3 and each subset based on grade and stage was analysed with R software via the Wilcox test. A *P*-value< 0.05 was considered statistically significant. We found that the expression levels of IGF2BP2 and IGF2BP3 continuously increased in each subgroup of grade except for grade 4 (Fig. 4a). Concerning the clinical stage, IGF2BP1–3 gradually increased in each subgroup, but there was no significant difference (Fig. 4b).

#### Univariate and multivariate cox regression analyses

**Fig. 5:**
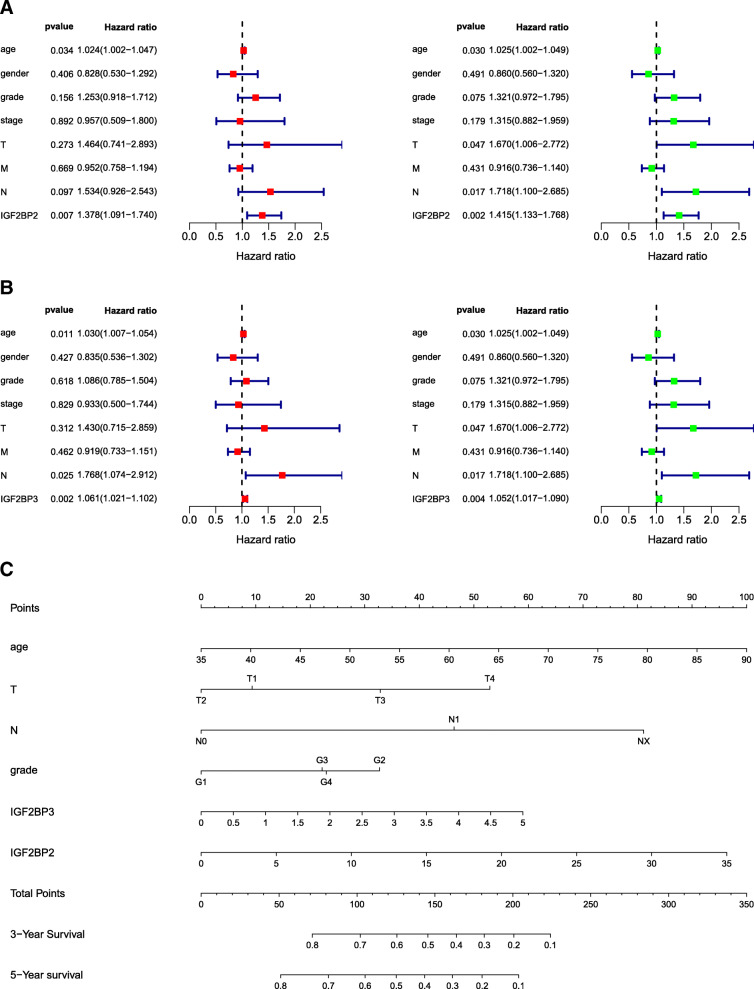
Cox’s proportional hazards model of correlative factors in Pancreatic Cancer Patients. **a** Univariate and multivariate Cox regression analyses of eight factors (age, sex, grade, stage, T classification, M classification, N classification and IGF2BP2) affecting overall survival. **b** Univariate and multivariate Cox regression analyses of eight factors (age, sex, grade, stage, T classification, M classification, N classification and IGF2BP3) affecting overall survival. **c** An established nomogram to predict the survival of pancreatic cancer patients based on the Cox model

Cox’s proportional hazards model was applied to analyse related factors that may affect the overall survival of pancreatic cancer patients, in which IGF2BP2 and IGF2BP3 were identified as independent prognostic factors (Fig. 5a, b). In both univariate and multivariate analyses, low expression of IGF2BP2 and 3 suggested improved OS. In the multivariate analysis, the HR of IGF2BP2 was 1.415, with a 95% CI of 1.133–1.768, and the HR of IGF2BP3 was 1.052, with a 95% CI of 1.017–1.019. Furthermore, based on the results of the multivariate Cox regression analysis, we established a nomogram model that may predict patient survival (Fig. 5c).

### Gene mutation information

**Fig. 6:**
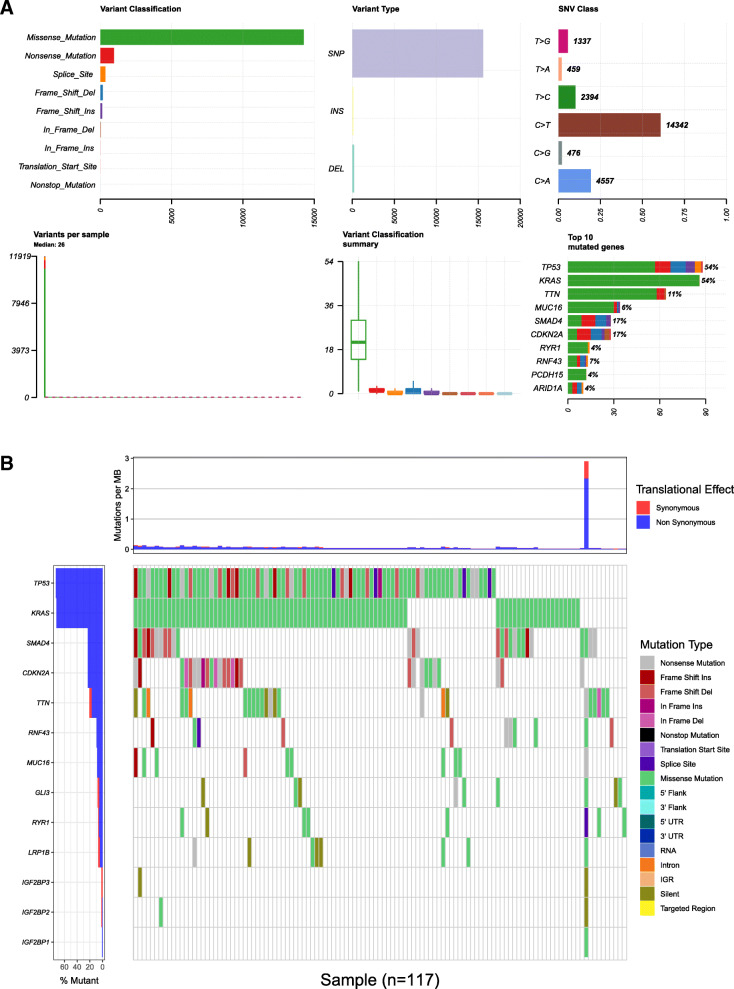
Information on gene mutations in pancreatic cancer. **a** Missense mutation was the most frequent mutation class in pancreatic cancer. SNP and C > T were confirmed to be the most fundamental variant type and SNV class, respectively. The median variation in each sample was approximately 26. The top 10 mutated genes in pancreatic cancer. **b** The mutation types of IGF2BP1–3 compared with the top 10 mutation types in pancreatic cancer

cBioPortal was utilized to calculate the gene mutation rate in pancreatic cancer samples from the TCGA database. In general, missense mutations were the most frequent mutation type in pancreatic cancer. Collectively, SNP and C > T were confirmed to be the most fundamental variant type and SNV class, respectively. The median variation in each sample was approximately 26. Finally, we determined the top 10 mutated genes in pancreatic cancer as follows: TP53, KRAS, TTN, MUC16, SMAD4, CDKN2A, RYR1, RNF43, PCDH15 and ARID1A (Fig. 6a). Then, by performing a comparison with the top 10 mutation types, we determined the most frequent mutation types of IGF2BP1–3, which were missense mutations and silent mutations (Fig. 6b).

### Gene set enrichment analysis of IGF2BP2–3

**Fig. 7:**
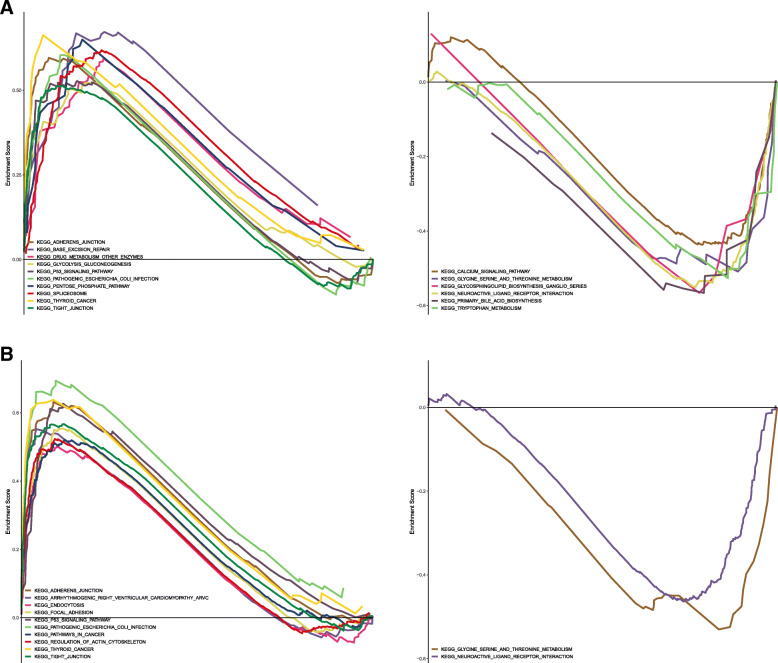
GSEA of IGF2BP2 and IGF2BP3. **a** The upregulated and downregulated enriched pathways associated with IGF2BP2. **b** The upregulated and downregulated enriched pathways associated with IGF2BP3

To fully understand the biological attributes of IGF2BP2 and IGF2BP3, we conducted gene set enrichment analysis. Based on the results of the GSEA, the top three upregulated enriched pathways associated with IGF2BP2 were as follows: adherens junction, pentose phosphate pathway and pentose and glucuronate interconversions. The principal downregulated biological pathways enriched in IGF2BP2 were as follows: primary bile acid biosynthesis, neuroactive ligand receptor interaction and glycosphingolipid biosynthesis ganglio series (Fig. 7a). The top three upregulated pathways associated with IGF2BP3 were as follows: pathogenic *Escherichia coli* infection, thyroid cancer and adherens junction. The principal downregulated biological pathways enriched in IGF2BP3 were as follows: glycine serine and threonine metabolism and neuroactive ligand receptor interaction (Fig. 7b).

### Gene functional enrichment analysis

**Fig. 8:**
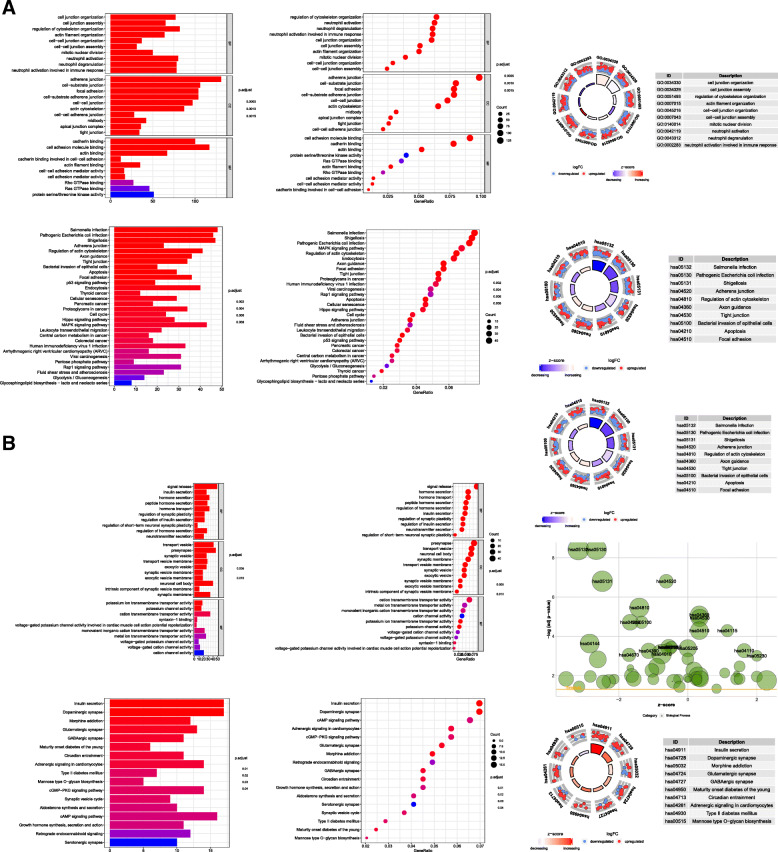
GO and KEGG enrichment analyses of IGF2BP2 and IGF2BP3 (differentially expressed genes); GO, Gene Ontology; KEGG, Kyoto Encyclopedia of Genes and Genomes

To fully understand the biological attributes of IGF2BP2 and IGF2BP3, we performed Kyoto Encyclopedia of Genes and Genomes (KEGG) and Gene Ontology (GO) analyses. We determined the biologically enriched genes, based on the results of DAVID, that are positively related to the expression levels of IGF2BP2 and IGF2BP3. In the GO analysis, the three biological processes in which genes positively related to IGF2BP2 expression are involved were as follows: regulation of cytoskeleton organization, neutrophil activation and neutrophil degranulation. The three cell components involved in these co-expressed genes were as follows: adherens junction, cell-substrate junction and focal adhesion. In addition, the three main molecular functions of these co-expressed genes were as follows: cell adhesion molecule binding, cadherin binding and actin binding (Fig. 8a). In the pathway analysis of genes that were positively related to IGF2BP2 expression, the top three enriched terms were as follows: salmonella infection, shigellosis and pathogenic *Escherichia coli* infection (Fig. 8a). In the GO analysis, the three biological processes in which genes positively related to IGF2BP3 expression are involved were as follows: viral life cycle, regulation of chromosome organization and regulation of mRNA metabolic process. The three cell components involved in these co-expressed genes were as follows: adherens junction, cell-substrate junction and focal adhesion. In addition, the three main molecular functions of these co-expressed genes were as follows: cell adhesion molecule binding, cadherin binding and transcription coregulator activity (Fig. 8b). In the KEGG pathway analysis of genes that were positively related to IGF2BP2 expression, the top three enriched terms were as follows: human papillomavirus infection, endocytosis and salmonella infection (Fig. 8b). Finally, the GO and KEGG analyses of biologically enriched genes that were positively correlated with IGF2BP2 and IGF2BP3 expression revealed the top 10 relevant biological processes, including cell junction organization, salmonella infection, mitotic nuclear division, and cell cycle (Supplement Figure 1).

### IGF2BP2 and IGF2BP3 expression and function in cell lines

**Fig. 9:**
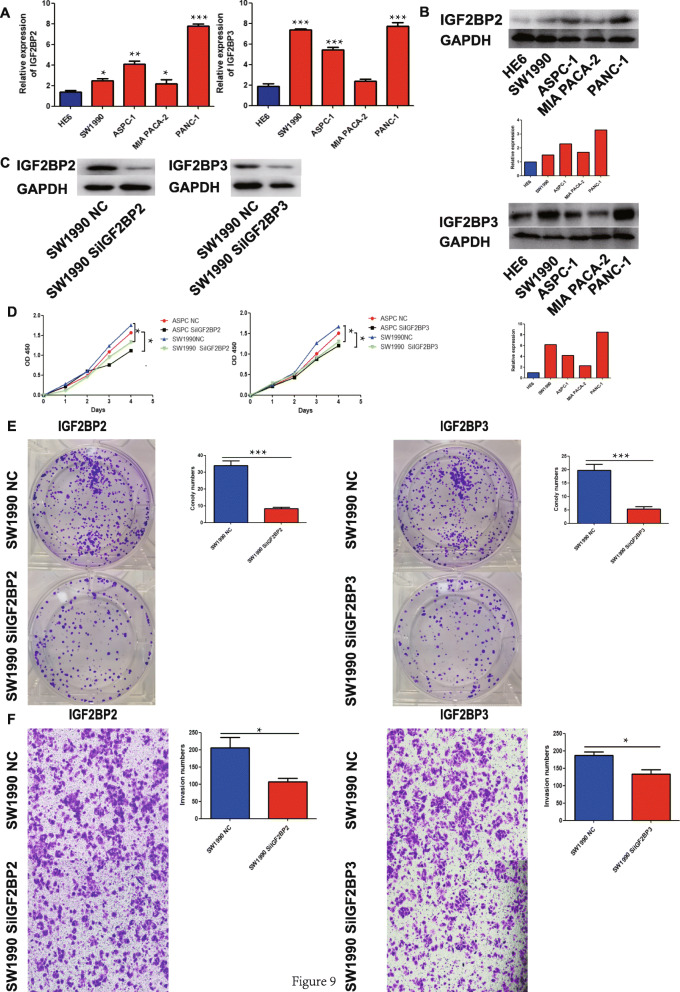
**a**. Expression of IGF2BP2 and IGF2BP3 in pancreatic cancer and normal cell lines. **b**. Expression of IGF2BP2 and IGF2BP3 in pancreatic cancer and normal cell lines assessed by western blot analysis. **c**. Proliferation of SW1990 and ASPC-1 cells detected by CCK-8 assays. **d**. Proliferation of SW1990 (NC vs SiIGF2BP2) (NC vs SiIGF2BP3) cells detected by colony formation assays. **d**. Invasion of SW1990 (NC vs SiIGF2BP2) (NC vs SiIGF2BP3) cells detected by transwell assays (magnification 400x). Data are presented as the mean ± SD of at least three independent measurements. *P < 0.05, **P < 0.01 and ***P < 0.001. *P* < 0.05 was considered statistically significant

To further explore IGF2BP2 and IGF2BP3 expression in cell clines, qPCR was performed. As expected, IGF2BP2 and IGF2BP3 proteins were significantly increased in pancreatic cancer cells compared with HPDE6-C7 cells, while the expression of IGF2BP2 and IGF2BP3 in pancreatic cancer cells was further increased (Fig. 9a, b). As predicted in the GSEA above, we inferred that IGF2BP2 and IGF2BP3 promote the proliferation or metastasis of pancreatic cancer cells to accelerate progression. The growth rates of pancreatic cancer cell lines transfected with IGF2BP2 siRNA and IGF2BP3 siRNA were significantly slower than that of cell lines transfected with the NC siRNA (Fig. 9c, d, e). In the cell invasion analysis, the knockdown of IGF2BP2 and IGF2BP3 significantly decreased the invasion rate of SW1990 cells (Fig. 9f).

## Discussion

In the past few years, despite tremendous efforts in pancreatic cancer research, the 5-year patient survival rate has not improved significantly. Patients with early pancreatic cancer have a good prognosis and can be cured by surgery combined with adjuvant therapy. However, most patients with advanced pancreatic cancer cannot undergo surgical resection alone. For patients with advanced pancreatic cancer, it is essential to explore more effective prognostic markers and therapeutic targets. Therefore, we screened the IGF2BP protein family through bioinformatics and conducted a differential analysis. IGF2BP2 and IGF2BP3, which are related to pancreatic cancer progression and survival, were further analysed, and their functions were verified in vitro.

In the preliminary analysis, three members of the IGF2BP protein family were identified to have differential expression between pancreatic cancer and adjacent tissues. Further analysis confirmed that only IGF2BP2 and IGF2BP3 were associated with pancreatic cancer progression. Therefore, only IGF2BP2 and IGF2BP3 were subjected to gene enrichment analysis to assess their cell compositions, molecular functions and biological characteristics.

In the gene set enrichment analysis, the base excision repair (BER) pathway was determined to be the most relevant pathway for IGF2BP2. Notably, the BER pathway plays a significant role in maintaining genome integrity, and many human health issues occur when any part of the BER pathway is aberrant [17]. This pathway begins with glycosylation enzymes and recognizes and excises lesions through the cleavage of glycosidic bonds [17]. Dianov et al. verified that aberrant P53 signalling could lead to failure of the BER coordination mechanism, APE1 overexpression and genome instability [18]. In our enrichment analysis, P53 was also upregulated, consistent with the conclusion of Dianov et al. Although the relationship between abnormalities in the BER pathway and the development and prognosis of cancer has been studied [19–21], in pancreatic cancer, whether IGF2BP2 is associated with this process has not yet been elucidated. The positive correlation between pathogenic *Escherichia coli* (*E. coli*) infection and colon cancer has been confirmed in multiple studies [22, 23]. The infection of pathogenic *E. coli* destroys the microenvironment of the microflora in the intestinal tract, thereby inducing colon cancer [22–24]. In studies of pathogenic *Escherichia coli* infection-induced pathways for pancreatic cancer, there is a lack of clear evidence that this pathway is associated with pancreatic cancer. The upregulation of IGF2BP3 expression in pancreatic cancer tissues supports research on this pathway. IGF2BP3 imbalance-induced pancreatic cancer may be related to pathogenic *E. coli* infection.

The autoimmune response to IGF2BP2 observed in hepatocellular carcinoma and colorectal, ovarian, and breast cancers supports the potential of autoantibodies against IGF2BP2 as biomarkers for cancer screening, diagnosis, and prognosis [5]. Consistent with the results of our Cox regression model in pancreatic cancer, the overexpression of IGF2BP2 in basal-like breast cancer and oesophageal adenocarcinoma predicts short-term survival for patients. At the cellular level, IGF2BP2 enhances genome instability and stimulates cancer cell proliferation and migration. Cao et al. believed that the dysregulation of IGF2BP2 was related to insulin resistance, diabetes and carcinogenesis and may potentially become a powerful biomarker and candidate target for related diseases [24]. In fact, IGF2BP3 might differentiate normal tissues from cancerous tissues and serve as a prognostic marker for colorectal, hepatocellular, and ovarian clear-cell carcinomas [25–27]. Previous research has confirmed that IGF2BP3 is involved in cell growth and migration in early embryonic development [28]. Similarly, both of our results confirmed the role of IGF2BP2 and IGF2BP3 in inhibiting tumour progression.

## Conclusion

In summary, we successfully revealed that members of the IGF2BP protein family can be used for the diagnosis and prognosis of advanced pancreatic cancer. Both IGF2BP2 and IGF2BP3 have great potential to become biomarkers for pancreatic cancer, as verified in patients. Although we explored the mutation types and possible carcinogenic mechanisms of IGF2BP2 and IGF2BP3 in pancreatic cancer, the mechanisms that promote the progression of pancreatic cancer need further study.

## Supplementary Information

**Additional file 1 **: **Supplemental Figure 1**. Top 10 pathways revealed by GO and KEGG enrichment analyses of IGF2BP2 and IGF2BP3 (differentially expressed genes); GO, Gene Ontology; KEGG, Kyoto Encyclopedia of Genes and Genomes. **Table S1**. Primers and SiRNA sequences used in this research (5’-3’).

## Data Availability

The datasets used and analysed during the current study are available from the corresponding author on reasonable request.

## Abbreviations

IGF2BP: The insulin-like growth factor 2 mRNA binding protei
TCGA: The Cancer Genome Atlas
GEPIA: Gene Expression Profiling Interactive Analysis
OS: Overall Survival
GSEA: Gene Set Enrichment Analysis
IMP: IGF-II mRNA binding proteins
KH: hnRNPK homology
GTEx: Genotype-Tissue Expression
KEGG: Kyoto Encyclopedia of Genes and Genomes
GO: Gene Ontology
DAVID: The Database for Annotation, Visualization, and Integrated Discovery
cDNA: Complementary DNA
CCK-8: The Cell Counting Kit-8
OD: Optical Density
PMSF: Phenylmethanesulfonyl Fluoride
DTT: DL-dithiothreitol
E. coli: Escherichia coli
