# Supplementary material for: Expression and prognostic analyses of the insulin-like growth factor 2 mRNA binding protein family in human pancreatic cancer
Source: BMC Cancer. 2020 Nov 27;20:1160. doi: 10.1186/s12885-020-07590-x (PMC7694419; doi:10.1186/s12885-020-07590-x)
Supplement: Supplementary file 1 — Additional file 1 : Supplemental Figure 1. Top 10 pathways revealed by GO and KEGG enrichment analyses of IGF2BP2 and IGF2BP3 (differentially expressed genes); GO, Gene Ontology; KEGG, Kyoto Encyclopedia of Genes and Genomes. Table S1. Primers and SiRNA sequences used in this research (5’-3’). [file 12885_2020_7590_MOESM1_ESM.pdf]

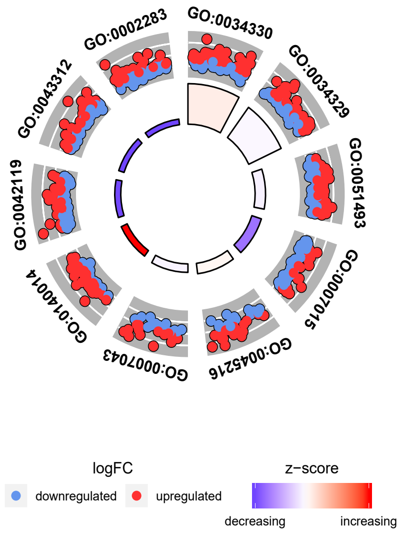

ID	Description
GO:0034330	cell junction organization
GO:0034329	cell junction assembly
GO:0051493	regulation of cytoskeleton organization
GO:0007015	actin filament organization
GO:0045216	cell−cell junction organization
GO:0007043	cell−cell junction assembly
GO:0140014	mitotic nuclear division
GO:0042119	neutrophil activation
GO:0043312	neutrophil degranulation
GO:0002283	neutrophil activation involved in immune response

IGF2BP2

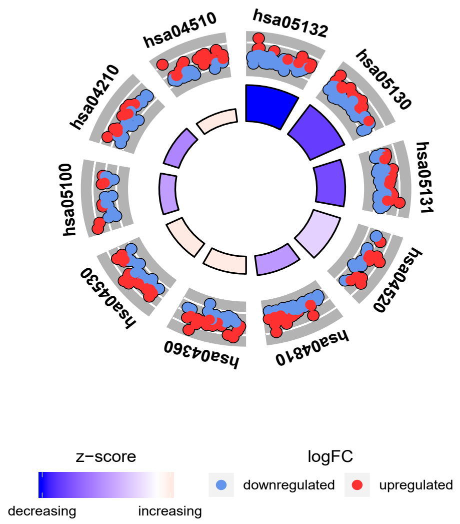

ID	Description
hsa05132	Salmonella infection
hsa05130	Pathogenic Escherichia coli infection
hsa05131	Shigellosis
hsa04520	Adherens junction
hsa04810	Regulation of actin cytoskeleton
hsa04360	Axon guidance
hsa04530	Tight junction
hsa05100	Bacterial invasion of epithelial cells
hsa04210	Apoptosis
hsa04510	Focal adhesion

IGF2BP2

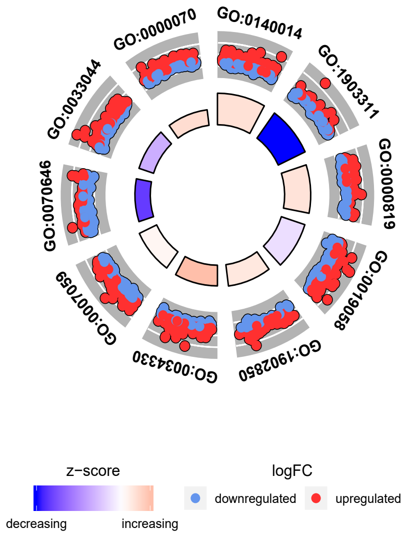

ID	Description
GO:0140014	mitotic nuclear division
GO:1903311	regulation of mRNA metabolic process
GO:0000819	sister chromatid segregation
GO:0019058	viral life cycle
GO:1902850	microtubule cytoskeleton organization involved in mitosis
GO:0034330	cell junction organization
GO:0007059	chromosome segregation
GO:0070646	protein modification by small protein removal
GO:0033044	regulation of chromosome organization
GO:0000070	mitotic sister chromatid segregation

IGF2BP3

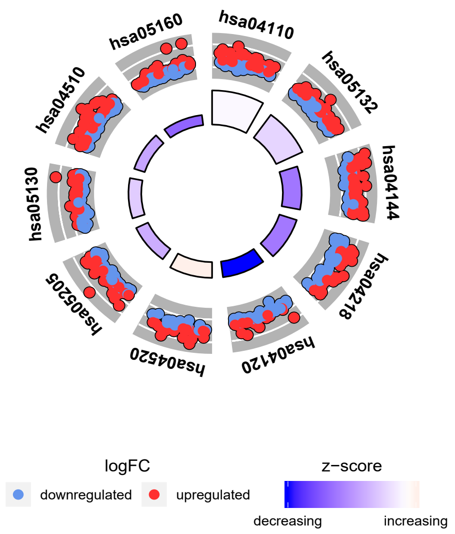

ID	Description
hsa04110	Cell cycle
hsa05132	Salmonella infection
hsa04144	Endocytosis
hsa04218	Cellular senescence
hsa04120	Ubiquitin mediated proteolysis
hsa04520	Adherens junction
hsa05205	Proteoglycans in cancer
hsa05130	Pathogenic Escherichia coli infection
hsa04510	Focal adhesion
hsa05160	Hepatitis C

IGF2BP3

Table S1: Primers and SiRNA sequences used in this research (5’-3’)

Name	Sequence
IGF2BP2 forward	5′-TGGCTTGGTTGGAAGACTGATTGG-3′
IGF2BP2 reverse	5′-GCCTGGGTTGGGATGAAGAGATTC-3′
IGF2BP3 forward	5′-GCTCTATCAGTCGGTGCCATCATC-3′
IGF2BP3 reverse	5′-CTGGTCACGAGGGACAACAACTTC-3′
GAPDH forward	5′-AAGGTGAAGGTCGGAGTCAAC-3′
GAPDH reverse	5′-GGGGTCATTGATGGCAACAATA-3′
IGF2BP2 SiRNA	TCGGGTAAAGTGGAATTGCATGG
IGF2BP3 SiRNA	AAGAGACAAAAGTAGTTTTTC

Gene	Related Genes	Correlation
IGF2BP2	E2F8	Positive
	KITLG	
	MREG	
	WNT7B	
	ARPC5	
	AK4P1	
	PADI1	
	SLC22A18AS	
	RELB	
	RAD51AP1	
	CLDN12	
	MAIP1	
	GPR157	
	CAMK2N1	
	UBQLN4	
	NTN4	
	TSPAN5	
	VPS9D1-AS1	
	ARAP3	
	HNRNPL	
	PPP1R18	
	BMP4	
	ACTN1	
	EIF3I	
	ANKRD10-IT1	
	LHB	
	RND3	
	TNIK	
	TMEM144	
	AC007283.2	
	LGALS4	
	TNIP2	
	ARHGAP11A	
	ZNF341-AS1	
	NFATC2IP	
	AMMECR1L	
	NFE2L2	
	FAM111A	
	BCL3	
	PYCARD	
	GCKR	
	AC026470.2	
	VGLL4	

AGFG1
AC130456.3
MAPK15
ARHGEF2
CENPU
NSUN5
C11orf86
SERPINB1
AL161431.1
CARD19
ANXA4
CD109
GYS1
PPM1J
DNM2
HMGA1P1
CHEK1
WFDC2
ACBD3-AS1
HIST1H2AC
FBXO28
SUMF1
XKR9
SUN1
YEATS2
TNNI2
COP1
CLNS1A
FTSJ1
CD82
FBXL19
FAM89B
IL20RB
OIP5
RASSF7
PPIAP22
LINC00705
CHML
CD47
TRIB1
PSMB10
CEACAM5
AC022165.1
PPP1R11

IRAK1
PRPS2
OSBPL10
PROM1
PRDM8
C15orf48
OGFOD1
TFCP2L1
MAP3K5
MMP11
VGLL1
METTL7B
NOL4L
OPTN
FBP1
MUC16
CLMN
SMOX
UBE2I
LPAR2
RPN1
RIPK4
SNORD89
PLK3
MYO19
ITPRID2
PFKP
SLC16A4
UBE2V1
DESI2
HSPD1
HIST2H2BF
PSMD13
FBXO34
LRG1
CHRAC1
CSNK2A2
RNA5SP18
PPIA
C8orf31
PTBP1
SHISA5
AC021078.1
SNORD12B

TM9SF3
TOP1
GTF2E2
B4GALT1
EXPH5
AC132008.2
MIR559
TMEM183A
TRIM31-AS1
PSCA
EIF1AD
GALNT7
PSORS1C1
OAS3
DHRS9
MYO5B
PVT1
NCBP2
FRMD4B
SATB2-AS1
ENC1
LMNB1
TGFB2
AC008687.2
BCL2L14
PLEKHA8P1
GJB5
SEPT10
ADORA2B
RAB5A
RPL37P1
LINC02257
BARD1
ILDR1
AC025154.2
OXR1
TIMM23
NUMB
RAB5IF
ARL5B
HNMT
MAP3K6
TGIF2
DDX27

IFITM10
ZNRF1
AC092611.2
SPTBN2
DUSP10
PANX1
LINC01572
SKIL
PACS1
RAD18
SLC44A4
C11orf91
TONSL
ZFYVE21
TNFRSF14-AS1
DIRC2
CDC42EP1
AC254633.1
RALBP1
OSBPL5
AP006621.4
GNAI3
OCIAD2
MCM2
AC025580.2
TRAK1
CTSV
VSIG10
EZH2
AFMID
AP000974.1
LIMA1
LDHAP5
CYTH2
C15orf62
MMADHC
UTP4
SAPCD2
AC012645.4
PNP
PLAG1
PACSIN3
NEK7
CENPK

RPS6KA1
MARCKSL1
SCEL
PALB2
ARRDC1
PPP1R3B
SIX4
KBTBD2
ELF4
TMPRSS3
STK38L
SUMO1
AL512274.1
PPRC1
ATL3
STAP2
ATXN2L
DIAPH2-AS1
SLC22A3
FAM114A1
IFITM3
STK26
GABRE
CCNB1
AC022211.1
ADAM15
HPS3
TCIRG1
PLEKHM1P1
EIF2S3
PELI1
UBE2E1
SUMO3
AC245041.1
BHLHE41
AC023043.1
ORC6
CRNDE
ZNFX1
LIN9
COLGALT1
AC005034.3
TTC31
MRGBP

CYB5R2
ADAM28
PPFIBP1
TMEM179B
CLDN16
RAB11FIP5
PPARD
KLK6
PLEKHA5
RNF207
IL10RB
THAP9-AS1
KDELR3
TRIM54
FAM110A
IKZF2
SIM2
IARS2
PTPRF
ARHGAP12
ACTRT3
GDPD3
PPIC
LMNB2
AP000695.2
PI3
RER1
KIF13B
LRFN4
ZNF488
PAFAH1B2
AL365181.3
PSMA1
SNAP23
TMEM30B
DDIT4
CST6
CYP4A22-AS1
SHCBP1
MUC5AC
EFNA1
OPN3
SUMO4
FAM214B

PRRG1
PARP3
AC078923.1
ADAM10
SLC52A2
MRPS10
FXYD5
PTPN14
SULT2B1
TSTA3
PTP4A2
NRAS
SPAG5
LINC00482
STX4
GPSM2
CASC8
PARP12
PICART1
FUT2
TMEM185B
TMEM241
ANXA8L1
BPNT1
NEAT1
DPY30
GDE1
TGFBI
AP003555.3
GPD2
FOXD1
LRRFIP1
RNU2-27P
NAT10
B3GNT2
AL606489.1
LIMK1
AL021578.1
MAPK3
TCN1
LINC01137
ARHGEF16
EXO1
AC022819.1

PSME4
ZFYVE19
TAP1
OCLN
KRT18P11
AL591806.1
ARHGAP26
SYNGR2
MRPL19
MAFF
HSPA1A
KISS1
DIAPH3
AC009133.1
ANXA8
BZW1
SPRED2
BROX
AC009065.5
YWHAZP6
HSPB1
VPS35
NAALADL2
GPATCH2
BATF
CTTNBP2NL
FAT1
TMCC1
TNFSF10
RNU6ATAC18P
AC009065.2
SLC6A20
DLG5
CEACAM6
SLC35C1
TNFAIP2
LPAR5
CEP70
DDX18
ARNTL2-AS1
UBALD2
SYPL1
AC093673.1
PRDX1

TRIM31
PCGF1
AC068580.2
MRPL3
MIR135B
DEPDC1B
TBC1D2
DTX3L
SMC4
HAS3
SLFN13
PKP4
FAM3C2
ITGB8
SPTY2D1
KIF15
DUTP1
ERN2
XRCC5
DPP9
DERA
DLG1
CYP2S1
MCM4
CDC6
MMEL1
CDC42SE1
CDA
GALNT10
SOWAHC
HOXB9
TM4SF1-AS1
GAPDH
RF00405
TOB1
TRAF7
CRYBG1
TMTC2
ADM
PRIM2
TANC1
PPM1G
FAM83A
KNOP1

FLNB-AS1
PARP14
AC093732.1
NFYA
AC022211.3
ARPC1A
LINC02014
BX470102.1
DPY19L1
DYNC1I2
RRAS2
FAM110C
DGKA
AL121772.3
CORO1C
FRMD5
NUP93
ADIPOR1
AC245041.2
TICRR
PSMD10P2
SERPINH1
PLXNA1
MFSD10
HOXB6
EIF4E2
VDAC1
BCL6
PDLIM7
EPS8L3
TMEM50A
GTPBP2
FBLIM1
CTSE
P4HA1
PSMB8
SRD5A3
WDR26
STEAP3
RARRES3
JOSD1
ANO6
ABLIM3
SLC35A2

MYORG
ARL4C
CENPE
ST3GAL1
SYTL2
PXN
TNFRSF1A
PRELID3B
AC073283.1
FUT6
CDCA2
MIR210HG
GART
ZC3H15
LINC02188
PPP3R1
CCDC9B
DNTTIP1
GALM
AGTRAP
FOSL2
EPHX4
AC106886.2
IPO7
SPTBN1
PRELID2
PLSCR1
ENDOD1
SLC22A20P
C1RL
SQOR
BCKDK
SNX7
KDM2A
INCENP
RNASEH1
PCLAF
C1orf198
ACTB
EML4
LITAF
TRIM21
RIT1
ABI1

DUSP11
AC008687.3
LDHAP3
ATP2C1
MTMR11
SGO1
NCAPG
AC005753.1
RN7SL130P
MISP
SLC35B2
AC010542.5
PLP2
DNAJC13
EPHA1
BDH2
POF1B
HOXB-AS4
HSBP1L1
CCDC58
AP001453.2
GCC2-AS1
EHD1
C1orf112
AC009237.15
PDCD6IP
RFC4
CDK2AP2
AL590666.2
DEDD
PTTG1
PTPRK
SSRP1
CCDC120
AC138150.2
HOXA-AS2
RNU6-26P
AC120498.4
LINC01186
TNNT1
AC105020.1
ATP2C2-AS1
HOXB7
IER3

DSC2
AP001033.2
SP140L
AC023043.4
AC133528.1
HEBP2
SPC24
CYBA
ARL14
AC068946.1
AC008687.4
MYO1C
MIR4435-2HG
ATIC
AC015660.1
ANO9
JAG1
KNL1
TTK
LGALS9
SFTA2
SLC44A1
CNKSR1
PPP1R15A
MFSD9
OSER1
TUBB
CPOX
SLC8B1
ZYX
AL590652.1
PICALM
SGMS2
MXD1
SAMD9
EFHD2
LIMS1
BLZF1
TSG101
PGK1
PTPRH
AC005041.3
ATL2
BCAR3

BACH1
EHBP1L1
MTHFD1
LINC00887
SH3TC2
USP43
ID1
CAP1
CYFIP1
FAM111B
MBOAT2
IL32
VWA2
LRRC59
WDR75
UBE2T
DNAJB1
C10orf55
YOD1
RHOV
EIF2A
AL138689.1
CTDSPL
MPZL1
RNPEPL1
AC108058.1
KRT8P12
CLIC3
GLRX2
RUNX1
TRADD
MCU
SYT8
ZW10
AFAP1L2
CENPN
ETHE1
ARF6
ZDHHC16
CCDC6
POLB
FADD
AL606834.1
KRT18P28

ZDHHC6
DOK4
SLC17A9
RALY
AMMECR1
TWF2
C6orf141
YES1
CAPRIN1
SEC14L2
AP003555.2
CCND1
LPCAT4
AC108134.1
TMEM54
CDCA4
FRRS1
AUP1
PIMREG
CORO1B
LINC02081
MICALL2
CAPN8
BUB1B
PPP4C
ZBTB7A
AP003068.2
PRR13
PSMD7
ESPN
PTPRU
CGB7
PTPRE
EHF
EFNA5
PPP4R1L
LTBR
HLA-V
HOXB5
GTF2IRD1
CDK7
KRT87P
CDC42EP5
CAPN12

AGR2
RIPK3
SLC50A1
METRNL
PARD3
VDR
ERGIC1
MMP7
SF3B4
NTF4
CASP6
B4GALT4
GIPC1
CGAS
TBX6
TMEM87A
BIK
NMU
STIP1
NCK1
AGPS
GRHL1
MLPH
CLDN23
BACE2
UBA6
ANXA1
AL365356.5
AC112220.2
CDH1
IFI27
CENPI
AL158166.1
AL035661.1
POLQ
LINC02298
CYP27C1
ESRRA
RAD51
PRPF40A
SMCO2
DTL
COX6B2
TRIM15

IFNGR2
KRT16
DCBLD2
AC073410.1
TXN
AL445933.1
SYT12
SYTL4
ANKRD36C
PKM
TMEM63B
LASP1
RAB10
PAK2
ABHD4
CREB3L1
GCNT3
PHLDA3
TMEM173
LRRC8E
LRP10
GPR35
MMP28
KNTC1
RHBDF2
PGM2
ABHD11
AURKB
ENO1
TRPV4
TFF2
HMMR
RACGAP1
KIF4A
STK3
STK38
ELF3-AS1
CAPZA1
AC103702.2
ARL6IP6
CARD11
NCAPH
RTKN
PAWR

FOXM1
TNIP1
CTDSP1
KLK10
OAS1
SPDEF
MAL2-AS1
ARAP2
BIRC5
CCDC68
GAN
NCEH1
HDAC7
NUTF2
KRAS
NUF2
IQGAP1
VPS37C
S100A2
AURKA
FAM83E
VCL
ANTXR2
ADAP1
TALDO1
MOB1A
STPG4
AVPI1
AMIGO2
RAB6A
SLC44A2
AFAP1
RTP4
SKA3
SH3D21
CRABP2
POC1A
VSIG2
VTI1A
CHMP1A
RBMS2
MGST1
FAM222B
PYGB

TTC9C
TROAP
RAP2B
S100A13
OSMR
AC068580.1
SH3BP2
ARHGEF19
HS3ST1
AK4
AC092964.1
AC092611.3
SLC22A18
SAT1
FAM136A
TMEM43
TMEM63A
NIP7
BHLHE40
LONRF3
CACUL1
CATSPER1
HERC4
FAM3C
PATJ
TMEM51
BAZ1A
SLC2A10
PDLIM1
ACY1
BCL9L
SFT2D1
KCNK6
USP4
CD9
KLF6
F3
ZFP36L1
PML
SLC23A3
STK24
TKT
ALG3
CEBPB

IQANK1
TSPAN14
REST
PKMYT1
ADGRF4
MANCR
RBBP8
YWHAZP5
CSTF2
CD58
SLC25A43
IL4R
PTBP3
GPN1
LINC00511
MYBL2
PHLDA1
KIF18B
SLC25A24
ESPL1
KRT80
AREG
MRPS6
NAPRT
ZPR1
TAX1BP3
RNF223
AL137782.1
CASP10
BEAN1-AS1
CSTB
LYPLA2
LINC02595
AC004923.4
OPLAH
PLS1
SLC16A1
BRI3
PRRG4
AC027031.2
HPCAL1
CACNB3
REXO2
RMI2

EIF3M
JPT2
ARPC2
HELLS
RAB38
AC007336.1
ARHGAP21
AL157838.1
RPE
ATP1B3
SLC35A3
AC106900.2
SLC39A4
ELMO3
FXYD3
MSL3P1
AC093162.2
GNG5
CMTM6
NAA50
IGF2BP2-AS1
FOSL1
SLC10A3
ATP11B
ZDHHC20
OASL
CIB1
CDCA8
RAD54L
CCNA2
CAB39
SH3PXD2A-AS1
ACTR3
TUFT1
RHBDL2
LINC01232
MARK2
CENPA
RALA
UNC93B1
PLAU
PLS3
AHNAK
CAPN5

	ARPC1B
	IRS1
	KPNA7
	TMC6
	DNMBP-AS1
	RNPEP
	SLPI
	CHMP4B
	C1orf116
	NAB1
	RCC1
	PERM1
	PPP1CA
	HCAR1
	STIL
	RAB27B
	TMEM41B
	IL1RN
	DUSP6
	ARHGEF5
	PRXL2B
	FANCA
	PGM2L1
	RNF168
	ZC3H12A
	RNF181
	ARHGAP42
	ALDOA
	ANXA2P1
	SH3BP4
	XPNPEP1
	SINHCAF
	FZD6
	RAC1P2
	EIF6
	BCL2L15
	BEAN1
	AC124947.2
	SLCO4A1
	KCP
	MYL12B
	KRT19P1
	SF3B6
	AP001453.3

CDK6
TRIM29
LBX2
CARS
MYD88
RBM41
EHD2
CNOT11
SPICE1
EREG
TET3
ARL6IP1
PRC1
BUB1
AC100861.1
DNAJB11
MMP14
CLDN7
TBILA
GPR87
MLKL
VPS37B
TNFRSF10D
AC068594.1
MIR222HG
CD44-AS1
EPN3
ADGRF1
PDP1
STEAP1
TPM3
NRM
AC068580.3
CLCF1
STAT6
POLD4
AC099850.3
RPSAP52
ABHD11-AS1
CHP1
GSDMB
TP53BP2
HIST1H2BD
MYO1E

AP005233.2
CTBP2
B3GNT7
TNFRSF21
SLC5A3
KIFC1
MAP3K13
CMTM1
SH3BGRL3
CKAP2L
CASP8
HKDC1
CKLF-CMTM1
LMNA
KIF23
KRT17
TPM4
5-Mar
VPS26A
CDC25C
SNX33
CDK18
PLA2G16
TRIM16
MUC20
BFAR
CTNNB1
SIAH2
SBNO2
ADAM9
KDM3A
JRKL
TRAF4
IL17RE
DPP3
PIAS3
SP100
ABHD17C
FAM160A1
DTX2
MGLL
GLUD1
KIF2C
SEMA3B

TFPI
LINC02323
PIK3CB
NT5E
CDR2L
MUC4
STK39
OTX1
TMEM123
LCN2
IER5
CAV2
LINC00857
POLR2H
GPC4
TSPO
ITPKC
HES1
UGT1A10
SMNDC1
ASPH
KIF18A
SDR16C5
EPB41L1
SLC39A11
PPP1R14BP3
FNDC3B
METAP1
AL451042.1
PARP4
FER1L4
KLF3
ERBB3
SFR1
BARX2
STX6
STRN
KCTD5
MAP4K4
GMPS
AC003965.2
RAB25
RETSAT
MKRN2OS

KRT18P10
ASPM
CNNM4
LINC00941
IRF6
CABP4
RIOK3
PITX1
ASF1B
EIF4G1
PLAT
TRIM5
RNF7
ACOT9
CDC42EP2
PPP1R14B
EXT1
C11orf80
PSEN1
PAK1
LRRC8A
ACSF2
LSR
ZNF217
CELSR1
RCC2
KARS
PRR11
TNFRSF10A-AS1
LINC02577
AC009237.3
UCA1
CYB5B
MKI67
VEZT
USP39
TLCD2
DSG2
COL7A1
GGCX
SEPT9
TNS4
PON2
RASEF

AP1S3
AQP5
USB1
NUSAP1
FAM83B
GRB7
KIF20A
APOL1
GLRX3
BAK1
TSPAN1
DTX4
AL590723.1
RNF39
IGSF9
NFKB2
SEMA4B
FSCN1
GPR39
SMS
DIAPH1
CBLC
ZDHHC7
RHOF
AC008760.1
REEP3
KIF14
AJUBA
LEMD1
ADAMTSL5
AC009237.14
TBL1XR1
EGFR
PPP4R1
ZWINT
MOCOS
CKS2
CENPF
KCNK1
HOXA3
ALPK1
AC114488.1
CFL1
ITGB5

	FA2H
	AC026401.3
	E2F7
	FERMT1
	CD44
	LY75
	KIAA1217
	IL15RA
	ZNF185
	TRIP6
	AC107308.1
	NMI
	EPS8L2
	PDZK1IP1
	NMD3
	MBOAT1
	MYH14
	AC019117.1
	TTYH3
	APH1A
	TTLL4
	SHC1
	MELK
	TMEM92-AS1
	MYEOV
	S100P
	SRPK1
	IGF2BP3
	TMC5
	SLC52A3
	TRPM4
	CCNB2
	PABPC1
	SDCBP2
	ERO1A
	NOB1
	SLC25A37
	KRTCAP3
	PGAM1P8
	VMP1
	BICDL2
	PLIN3
	TSPAN6
	VILL

CNIH4
CBX3
ANKRD22
DLGAP5
CDCA5
PRR15
FOXC1
TSPAN15
STAU1
TAGLN2
RRM2
ERLIN1
ALMS1-IT1
MSLN
TMEM189
SLC20A1
TTC7A
BTBD10
DHCR24
P2RY2
TSKU
ELOVL1
SMAGP
CDC20
LPCAT2
FLNB
PROM2
CORO2A
ETV4
TOP2A
GRTP1
TJP2
TRIM8
KRT8
F2RL1
ADGRE5
UHRF1
BZW2
TMOD3
LRRC1
PLA2R1
AL355312.3
DHX32
PIEZO1

ELF3
PLBD1
IL1RAP
MYL12A
RPS6KA4
QSOX1
AC046143.1
GALNT5
NQO1
XDH
BCL10
CHMP4C
PCDH1
ESRP1
KIF11
HTATIP2
GALNT3
LDLR
CKS1B
AHNAK2
TRIM47
FRMD8
FGFRL1
CLIC1
BCL2L12
MDFI
TFAP2A
SLC1A5
TGM2
UNC13D
COL17A1
EFNB2
H1F0
PAQR4
PLK1
CAST
PTGES
IL18
NEK2
TAF1D
GJB2
ETV6
SPINT1
ABTB2

KRT15
MICA
KPNA4
MIR4653
INSIG2
EFNB1
SP1
FUT3
MALL
CNN2
CHMP2B
ARMC10
SOX4
ACTL6A
CD2AP
SRC
INF2
TJP3
RARG
TMSB10
ACSL5
TP53I3
GJB4
SHB
MPZL3
SH3RF2
OR7E14P
TMEM44
UBE2E3
SMURF1
CGN
NKILA
ADSS
ARHGAP27
PMEPA1
CRYBG2
SLC39A1
ERBB2
OVOL1
SMAD3
AGRN
DOCK5
MAL2
S100A6

CDK1
RIPK2
CASP4
OXSR1
CLDN4
CKLF
STYK1
ECT2
LMO7
ASAP2
CTNNA1
DKK1
PROSER2
SLC35F2
LINC02041
TES
ITGA2
AHR
RCE1
LAMA3
PARD6B
ANO1
LBX2-AS1
HNRNPF
YAP1
ATG16L1
BAG3
SFXN3
ALS2CL
AL451042.2
STAMBP
PLEKHN1
DHRS3
ESRP2
ZDHHC3
ADGRG6
BLACAT1
PDCD10
TMEM105
TMEM159
FAM83H
KCMF1
INPP4B
SH2D4A

FAM129B
ADAM8
PTPN12
ITPR3
PEX13
TFG
NGEF
PRICKLE3
S100A14
HRH1
EPS8L1
TMBIM1
GSTP1
PERP
NPAS2
ALDH3B1
NDE1
NFE2L3
CD151
BCL2L1
MACC1
ARHGAP32
ABCC3
RHOC
IRAK2
RAC1
TPX2
YBX3
B4GALT5
HK1
MGAT4B
SLC16A5
RYK
ZDHHC9
DNMBP
PTMA
GBP2
ANLN
EPS8
MROH6
PLAUR
GALE
MUC1
HMGA2

CLDN1
UBE2C
STN1
PRKCI
IQGAP3
LY6E
KRT18
ANXA3
C3orf52
B3GNT3
TNFRSF10A
EFNA4
TNKS1BP1
PATL1
SLC2A1
S100A10
TMEM139
RHPN2
RNF149
VAMP8
MELTF
TNFRSF12A
PPARG
PTTG1IP
ANXA11
SPATS2L
EZR
TMC7
TK1
SERPINB5
TOR4A
ARNTL2
PLEC
SOX9
ZDHHC5
CAPN2
B3GNT5
HJURP
AL049555.1
MPZL2
JUP
VASP
PKP3
CTNND1

TM4SF1
APLP2
PLEKHA7
TMEM41A
SLK
GNA15
TNFRSF10B
YWHAZ
SDC1
JPT1
SLC16A3
HK2
EVPL
TCF7L2
PPP1R13L
PHLDA2
RALB
RELA
FOXL1
TGIF1
KDM5B
NECTIN4
CMTM7
ITGB6
CEP55
KRT7-AS
ITGA6
PLCD3
TLDC1
MVP
FHL2
LIPH
HDGF
GRHL2
KIAA1522
RRAS
TMEM92
TPBG
TEAD3
CAPG
CTTN
PLCB3
SSH3
TACSTD2

SCNN1A
ST14
TUBA1C
LGALS3
LAD1
BAIAP2L1
C6orf132
MYOF
FOXQ1
LAMC2
CDK2
TMPRSS4
ANXA2P2
PLPP2
RHOD
INAVA
S100A16
F11R
RHBDF1
EPHA2
LDHA
OSBPL3
ITGA3
ACTN4
ANXA2
SERINC2
KRT19
CDH3
EPHB4
S100A11
GPRC5A
PLEK2
SFN
PRSS22
NET1
HMGA1
MST1R
KCNN4
FGD6
PRSS8
PTK6
SH2D3A
CDCP1
KLF5

	SDC4	
	C19orf33	
	GJB3	
	CAPN1	
	TRIP10	
	TINAGL1	
	LAMB3	
	KRT7	
	ITGB4	
	TGFA	
	MET	
	RAB39B	Negative
	TSPAN7	
	MROH8	
	OTUD7A	
	AC106795.2	
	NAP1L2	
	APLP1	
	MAGEE1	
	IQSEC3	
	PACSIN1	
	SCML2	
	RTN1	
	SEC11C	
	DLGAP3	
	FGF14-AS2	
	NOVA1	
	ATP6V0E2-AS1	
	KCNH6	
	DIRAS1	
	PDZD4	
	ACSL6	
	ZNF540	
	SERPINI1	
	UNC79	
	EDA	
	SYP	
	MAPK8IP1	
	SPTBN4	
	ACTL6B	
	TMEM63C	
	AC027575.2	
	TCEAL2	
	ATP2B1-AS1	

PAK3
MPP2
XKR7
ELAC1
SNAP91
FAM218A
TENT5C
MSI1
CELF4
ZNF793-AS1
MYT1
CYP46A1
CXorf57
AC023509.4
AMER3
SNPH
AC007541.1
RUNDC3A
UNC13A
AC012313.2
GPRASP1
KCNB1
CACNA2D2
EPHX2
LINC00683
NFASC
AL021368.3
AL162377.1
CDO1
AC008808.2
ZNF582
AC233976.1
AC012146.1
ZNF491
C5orf38
TCEAL5
JAKMIP2
VLDLR-AS1
SLC4A8
BSN
CCDC92B
IL6R-AS1
FAM69B
SVOP

GPX3
SDK1
CACNB2
GNG2
AC006369.1
LINC02106
ADGRG5
AC022893.2
AC008669.1
AC104985.2
SPTB
SLC8A3
EML6
CACNA1A
DSCAM
NCALD
DPP6
LRRC4B
NAP1L5
GSTA4
NOL4
FAM117A
USP51
RTL5
PNMA8C
GPR150
DUSP15
ICA1L
GPR162
SCN3B
TMCC2
SLC25A42
AL158055.1
LYRM9
RAB3C
LINC01431
TSPAN33
NIM1K
PIPOX
CHRNB2
SEZ6L
CD99L2
FMN2
CELF3

AC074032.1
OGDHL
TUBA3FP
HLF
NAP1L3
AC008115.4
ZNF667
IRX2
AL133343.2
SLC22A17
SERPINA10
RIC3
ZCCHC18
ZSCAN16-AS1
CYB5D2
DUSP26
SCOC-AS1
AC005076.1
GLIPR1L2
QDPR
CCDC188
AC005696.4
HS6ST3
CLGN
WSCD1
KCNK3
MAGEH1
GTSE1-DT
LINC01128
CERS4
AC117489.1
LIFR-AS1
LINC02559
B4GAT1
INSM1
LINC02447
MTUS2
KCNJ6
GKAP1
SLC12A5
FBXL16
PPP1R1A
ANGEL1
LHFPL4

SSTR3
ZNF483
AL023806.1
RIPPLY2
CALY
SLC25A5-AS1
FAM155B
AC008443.4
C17orf51
SGSM1
AP3B2
CELF5
RIMBP2
AL035701.1
ZNF667-AS1
BEX4
FAR2P2
ATP1B2
TMEM179
MAPK10
GPR135
REEP2
SSTR2
AC138965.3
RBM11
CYP51A1-AS1
ST18
ZNF236-DT
MAP6
SLC26A11
AC156455.1
INA
USP27X
AC079089.1
AC010501.1
CCDC13
AL139353.1
SLC29A4
LINC01963
NRL
SLC8A2
FAR2P3
CRIP3
CDK5R2

BEX1
A1BG-AS1
FXYD6
AC092437.1
AC015961.1
FAM135B
UNC80
AC078925.4
BCDIN3D-AS1
ATP1A3
SLC38A4
LINC01484
SOCS2-AS1
CPE
AC009185.1
ATP6V1G2
AC140481.1
KCNJ2-AS1
ASB16
AP001486.2
CXXC4
ZNF181
AL137779.2
AC110609.1
GNAO1
ADGRF3
AL133299.1
NUCB2
SEZ6
AL161938.1
ANKRD7
SLC7A14
C22orf39
STXBP5L
ZFP2
KCNJ11
LRRC10B
FAM167A
LINC00957
AC010478.1
LINC01586
SYT4
GRIA2
ELOVL4

CBX7
SNAP25-AS1
HFM1
KCNMB2
RFXAP
FAM184A
LINC00870
CRMP1
MTMR7
APBB1
WDR17
AC010307.4
CAMKK1
DDX25
FGF14
AL158163.2
LINC02101
ATP2A3
AC016582.3
AMPH
RIIAD1
KCNA5
AL645728.1
PNMA3
CPLX2
ADCY1
VASH1-AS1
PPM1K
SEPT5
RFX6
AC126177.7
P2RX6
SCAMP5
AC084033.3
RAB9B
FAM222A
AC104985.1
VWA5B2
DNAJC18
FBLL1
PAXBP1-AS1
SYT5
AC126177.6
GCK

NRG2
AC120049.1
AC105277.1
C16orf96
TRIM52-AS1
MAPK8IP2
SNAP25
ZNF441
CDIP1
AC090617.5
KIF1A
RUNDC3B
AC026369.1
AC099684.1
AP000757.1
GDAP1L1
AC048382.5
SHISA7
FBXO15
AC112204.3
ASTN1
ZNF781
LINC01014
NRXN1
SLC12A5-AS1
RCAN2
KIF5A
PTPRN
NEURL1
TECTA
WNK3
KLF2P2
LRRTM2
KIF5C
GPR142
VAMP2
BSN-DT
LINC00663
AC116614.1
ACACB
RND2
LINC00909
A1BG
TAT-AS1

GRM4
PHF21B
EFR3B
AL133325.3
FBXO10
FRRS1L
HMGCLL1
AL589765.1
AC005225.4
AC005498.2
TMEM145
LINC01146
MIR7-3
AC106795.3
COQ10A
ADGRB3
HHATL-AS1
INSYN1
CYFIP2
AC112204.1
AL122008.3
AC005498.3
KLHL22
AL391807.1
AC095057.3
INKA2
GRM1
EFNB3
ZNF778
ZNF853
C19orf81
GPRASP2
DPY19L2P4
SLC25A53
SCN3A
GRIK5
KSR2
RPS27P25
CCDC13-AS1
KHDRBS2
AC124319.3
AL845472.2
ASPDH
CIRBP

AL022337.1
4-Mar
DHRS4L1
AC016820.1
EID2B
CNKSR2
ELMO1
SALL2
AL391261.2
TMX4
AC113554.1
ZNF157
PNMA8A
MIR7-3HG
PACRG
NANOS1
CAMK2B
SLC6A17
APOH
STK33
MOCS2
DNAI2
GRIA3
AL121929.2
AC004656.1
MAP3K15
SYT3
AC025175.1
TTC7B
SH3GL2
EPM2A
HRAT5
AC087752.3
GPR148
LGI3
SLC1A4
MIR670HG
AC090061.1
DNAH9
AC129507.3
NEXMIF
RGS9
OXCT1-AS1
FMC1

AC091891.1
NOVA2
ATP6V0E2
RASA4
AC233723.2
AF279873.3
RBM26-AS1
AL358933.1
PPP4R4
AL034550.2
AL355994.2
TMEM88
AC114271.1
ERO1B
CDH10
ACAT1
MAGI2
ADGRL1
CNIH2
NKX2-2
AC007495.1
AC008808.1
ARMC2
UBE2QL1
TMEM121B
AL354920.1
SLC7A8
TOGARAM2
ZDHHC15
AC091271.1
AC226101.1
KIAA1324
NXF2B
ABHD14A
TMOD1
AL691432.2
NCAM1
CAMK2N2
XKR4
C3orf14
UCHL1
CCDC178
CPT1C
LINC01927

WNT4
PTPRN2
ABHD15
AC091825.1
AC000068.1
AC002511.1
SERP2
ENHO
AL139274.2
AC008438.1
AC011477.7
DCT
LINC01370
GPLD1
AC006116.10
FGF12
GALNT16
ADGRD1-AS1
AC073857.1
AC012313.5
NECAB2
FITM2
CCDC151
NLRP1
GNAZ
AC087457.1
AC004832.1
KCNB2
NT5M
DZIP3
F10
TRAF3IP2-AS1
IPO5P1
AC068473.5
LRFN1
HDHD2
ABCC8
PSMG3-AS1
BEND5
ARX
AC005355.1
PHF24
AC005034.4
AC097468.1

KLB
RNF180
SBK2
EXOC3-AS1
CPEB1-AS1
NAT8L
ANKS1B
SMIM27
TMEM59L
AL606970.1
AC104825.1
C15orf61
TMEM151B
COL26A1
PPP2R2B
RPH3AL
LINC00158
MTURN
PPM1E
MAMLD1
LINC01284
ZDHHC22
GLS2
FSTL4
C10orf143
LINC00334
AL121601.1
AL355877.1
ARHGEF7-AS2
AC002511.2
PRRT3
ZNF287
KL
CERS1
AL583856.2
LIPE-AS1
AC008761.3
TDRP
CPA5
SESN1
GS1-124K5.4
TMEM132D
GCH1
TCP11X2

C17orf107
KCNC1
AC012508.2
ATRNL1
AC004877.1
SLC2A11
AC006033.1
EPX
AL845472.1
AP003559.1
TTC41P
GDAP1
RWDD2A
AC036176.1
FAM104B
ZBED3-AS1
ZNF658
AC107081.1
AC099521.1
TCP11X1
TRAM1L1
BEX2
POLR3H
PCP4
AC092574.1
SNTG2
IRX1
AGBL4
KLHDC1
JPH3
AC091152.4
SAXO2
AC006012.1
PCDHA1
SH3GL3
ATP8A1
ELAVL4
AL390208.1
PCSK2
PPP1R42
KCNN3
GNG7
ZNF583
CTXN2

CD200
ZNF582-AS1
AC068338.2
AC239367.3
SEC14L5
DTNA
AP003721.4
MAST3
AC106795.5
SCG3
AC063926.1
KIAA0319
FAM110B
AL133419.1
SNTG1
AL022332.1
AP000892.2
PCDHB1
SCG2
AL596244.1
F7
FKBP11
ACTR3B
AC092119.3
AC022417.1
AC009163.6
CASKIN1
PTPRT
AC027807.1
ABAT
FOXP2
KCNH3
MARK1
PAX6
AC096733.2
NBEA
TRIM9
AC125257.1
AC006538.1
CFC1
C1QL1
NPAS3
PEMT
AF111167.2

CACNA1B
SYT14
FGF17
AC127070.1
GARNL3
C3orf18
AVPR1B
AL512791.2
KCNN2
AL442663.3
CPEB1
AC129507.2
ERVK9-11
C22orf42
LINGO4
FO393419.3
DNAI1
AL133325.2
AL009181.1
SLC6A16
SLC6A4
PPP1R3F
SRR
KIF6
FAM227B
TRO
CHST10
TEX38
MAP7D2
ZNF280B
AC010624.2
MGAT4C
CAND2
C21orf58
NYAP1
SSR4
AC015967.1
TSPYL2
ZSCAN18
AC116003.3
MAST1
AC009102.2
KATNAL2
CYP4F24P

AC005224.1
AC021242.3
CCNA1
MXD4
AL138690.1
10-Mar
MLYCD
RNU7-171P
TUNAR
AC120498.10
AL049796.1
TMEM178B
TNRC6C
AC079610.1
FYB2
RTN4RL1
FAM120C
AC104051.2
MIR153-1
SYNGR4
TMEM74
HAO1
TMEM198
SARM1
AL121672.2
PLPPR1
AC012158.1
MYL7
ZNF709
VWC2L
C14orf132
PELI3
LINC02458
KBTBD6
LINC00644
AC098484.2
LYRM7
MEG8
ATP8A2
PCSK1
ZNF671
LRRC4
DKFZP434H168
AC100793.4

GHR
AC007563.1
FAM57B
LRRC37A5P
LINC00643
SCG5
DPH1
AL031595.3
SOCS2
OSBPL6
AC109454.2
LRP2BP
FICD
AC010327.4
AL603910.1
AC005730.2
LRPAP1
PART1
UBE2D4
ZNF569
MAPT
GABRG2
TMEM170B
RAPGEF4-AS1
RAB3A
FAM184B
ANK2
SMIM10L2A
FRY-AS1
KCNJ8
ARSG
C18orf32
PDCD6IPP2
LANCL2
LINC01942
PITPNA-AS1
AL031658.2
B9D1
AC037459.2
DRC3
JAKMIP3
SLCO5A1
AC008966.1
AL589765.7

LNP1
LINC00900
AC096734.2
AC126283.1
PTENP1-AS
LINC01531
PLD6
AC007221.1
NR0B1
LY6H
PRLR
CRHBP
MDH1B
ARHGEF26-AS1
USP2
RSPH9
SNCB
UNQ6494
EPB41L3
DPH6-DT
SPATA4
DNAJC27-AS1
AC093510.2
WASF3
C9orf147
ZNF821
MED9
LINC01018
CADPS
AL662844.4
ZNF467
LOH12CR2
AC010980.2
AC012513.1
SELENOKP1
GPR158
AC009126.1
FAM131C
FSIP1
AC012513.3
SEPSECS-AS1
ISL1
AC079385.3
AF186192.2

ZNF843
AC015819.1
SCGN
LRRTM4
AMIGO1
ZSCAN1
ARNT2
AL133520.1
TMPRSS6
AC091167.4
RLN1
KCNJ6-AS1
AC008743.1
TACR1
AP002847.1
NRSN2
WDFY3-AS2
DNAJC6
DMRTC1B
METTL16
CARMIL3
TXNDC15
BX284668.2
LINC00641
AC026471.2
DYNC1I1
JAKMIP1
BTBD17
LINC00907
ZNF660
AC093642.2
AF186192.1
RAB6C
11-Mar
ZIM2-AS1
DRAIC
ATP2B2
CCM2L
SLC35F3
NRCAM
LRRN3
ZFP3
PRKN
TMEM196

PPP5D1
NUTM2B
AC010980.1
BX842568.4
AC117457.1
ITPR1-DT
AC090241.3
SLC43A2
CCDC110
AC025162.2
HAR1A
AC015961.2
NIPSNAP3B
TUB
AC239585.2
KANK1P1
BX649632.1
Z97653.1
LHFPL1
LINC01522
Z97989.1
PDXP
ANGPTL5
SNTA1
NUDT9
AC133540.1
EID3
TMEM151A
CAPSL
SYT7
RXYLT1
GALNT13
BRINP2
FFAR3
HEIH
AC112219.2
AC015726.1
KCNJ3
RNF157-AS1
SCRT2
CGRRF1
AC012640.2
AC010226.1
GPC5-AS1

AC007938.1
C5
AC107952.2
AC063926.3
NEUROD1
CACNG2
USHBP1
KCNH2
CCDC62
AC069224.1
PPP1R3E
USP27X-AS1
ZBED3
TTC23L
ZNF542P
GRK4
DYNLT3P1
AC092720.2
S100Z
CACNA1C-IT2
ADGRV1
TMED8
AL353743.1
AC023762.1
AC116609.1
ZKSCAN2
USP20
GPR119
AC008403.3
ASXL3
ZNF596
RTL9
SPATA7
MAN1B1-DT
DNAJC28
RLN2
CHGB
AC004836.1
AC064801.1
AC008667.2
SLC45A2
SLC16A12
ERP29
ZNF835

CLIP3
BEX5
EFCAB8
AC138356.2
AC005962.1
AFF3
FGFBP3
AP001267.3
AC104986.2
PKMP3
CACNA2D3
SRSF12
BRD3OS
RN7SL832P
AC124016.2
TCEANC
NDUFA6-DT
PCDHB4
OAZ2
AL121583.1
ZNF497
MVB12B
PCLO
RAB39A
AL022313.4
PCSK1N
RPL10P11
GDF11
RPL34-AS1
PRADC1
UCN3
AL137028.1
CFAP53
AC097641.1
AMER2
AL117339.4
AC011444.2
AC004023.1
NAT16
RAMP2-AS1
KAT14
CCDC184
DMGDH
AC007686.3

ZDHHC11B
AC024075.2
AC127070.2
MED14OS
AC066612.1
SARAF
CRYBA2
AC002451.1
AF111169.3
FTCD
RTCA-AS1
AC005837.1
AC245297.1
DSCAML1
STXBP1
SYNGR1
IRAK1BP1
GAD2
ZCWPW2
LRP3
RGS7
AL022068.1
AC027281.1
TNR
AC022107.1
RIMS2
AC079140.3
SV2A
SAYSD1
AL161772.1
AL356489.2
VLDLR
TOM1L2
CRY2
LINC01863
AL357146.1
LINC00271
APH1B
TTC25
KCNMA1
CCDC175
AF106564.1
KIF9-AS1
ZNF132

TMEM254-AS1
AC097461.1
LONRF2
AL354707.2
C18orf65
CBX6
RGS22
CCKBR
AC087500.1
ZNF554
ARIH2OS
ST8SIA3
AC103736.1
AL162171.1
KCND3
LRRC37A3
AC104333.3
AC105339.1
FAM122A
HCN2
PCBP3
TBL1X
CYP4F62P
NAMA
CROCC2
ZNF571-AS1
AF131216.4
C1orf158
RPS20P22
CKMT2-AS1
CYCSP8
RFPL2
ZNF568
DCLK2
HMGN2P15
TENM1
AC008050.1
RAPGEF4
MAN1C1
NMNAT3
NME5
AC108475.1
RAB26
AC121761.2

AL035071.1
AC005920.3
ALDH5A1
MYO3A
AL512604.3
TTLL11
AC015540.1
CNNM3-DT
AC009570.1
AC012508.1
AL096711.2
LINC01715
QSOX2
NPR1
AC104806.2
PSPN
NLGN4X
AC063926.2
KARSP2
AC087071.1
AC006116.8
VGF
AL049543.1
SYNE1
ADAM29
TATDN3
LINC00526
ATCAY
ALG14
AC004597.1
SOCS2P2
LINC00602
SLC25A4
SETBP1
AC121338.2
AC006449.6
AL138688.2
PCDHA2
SRCIN1
AC026691.1
ZHX3
EGFL7
AC064807.2
AQP4-AS1

AC110998.1
MIR670
DNAH6
MTNR1B
ST3GAL3
LINC00476
PARD6A
HS3ST4
REM2
CEP126
SLC7A2
SCRT1
AC109454.4
MAGEL2
CDADC1
RBP7
TP53TG3D
RASD1
AP000802.1
SOX5
LY6G5C
AC005062.1
AC243830.2
TAGLN3
C2orf73
AC104109.2
ESRRB
AC008758.6
RNF157
LSINCT5
ST7-AS1
SERTM1
AC020558.2
AL356599.1
CFAP61
SRP14-AS1
AC012313.8
AC138965.2
AC103952.1
AL359710.1
AC025871.2
PDE3B
OR7E47P
FAM228B

MOB1B
AL118558.4
IFFO1
PRKAR2B
AC012360.1
PLCXD3
PCDHA3
AC007780.1
LINC02495
ZNF844
PARVB
HPCA
METTL7A
AC034243.1
AC091153.3
IGLVIV-59
CERK
POMT1
GABRB3
PSD
AL136304.1
USP30
MCF2L2
SLC6A13
SLIT1
AC073316.3
CD320
SFRP1
C12orf60
ISCA1
RN7SL336P
AC020934.1
TMEM232
AL355297.3
TPPP
ERICH3
AC010536.3
AC046185.2
ANGPTL3
DLG4
AC009812.1
AL157788.1
AL359764.1
PGBD5

KLF2P3
AL022324.2
EML5
CLUL1
AC105020.5
AC024257.5
SDHCP4
LIPJ
AC064807.4
COPS3
AC012213.4
BRSK2
OLFM1
TSPEAR
SERPINE3
CRTC1
SH2D3C
LINC01976
HAR1B
PRR34
CDS2
AC016705.2
AC004947.2
KLKB1
SOBP
HDHD5-AS1
NKX6-1
FAM229B
AC106047.1
AL358781.2
AC113615.1
RGS11
ZNF602P
AC012213.1
CHRM5
SMIM32
PABPC5
AC011290.1
MAP2
TAT
LAMA5-AS1
PTGDR2
SLC12A1
GPR27

SCAMP1-AS1
AC011899.1
FLG-AS1
DIS3L
ARL15
ZMAT1
GJD2
KNDC1
TMEM38B
ATP9B
CACNA1C
SLC9B1
TBC1D13
ZSWIM2
AL117192.1
AC243562.2
MEG9
SOCS7
OTOGL
TCTN1
CAPS2
TCEAL3
NAPB
STXBP5-AS1
AP002360.1
AC010624.1
ZC3H10
AL451085.1
TRMO
VN2R17P
MYO16
AC023632.2
HSD17B14
TCEAL1
SIL1
CLU
WDR7
AL359513.1
ZNF429
CXorf58
RFESD
PPFIA3
AC096644.1
TMOD2

FAM153C
CETN4P
AC055811.2
GEMIN7-AS1
AC011008.2
KCNK16
AC107982.3
SPAG6
FAM171A2
AC105052.4
PEG3
SPINK9
HCG2040054
SLC35F4
ZNF674-AS1
SLC23A2
AC008543.5
ULK2
OPRD1
C5orf49
ADCK1
AC011477.3
RTBDN
LYSMD2
GPR179
ZIK1
NXF5
RAB6C-AS1
DISP2
RAMP2
AP003108.5
TIMM22
LINC01529
FFAR2
AL353746.1
AL161658.1
NACAD
RNF182
C8orf86
COX6CP13
PWRN4
PLPPR2
SLC46A1
UCHL1-AS1

LRRC9
PAIP2
CYP4F30P
RAB36
AL592293.1
AL645924.1
CYP4F32P
MICU3
AL355990.2
FEM1A
SEMA5A
AL513165.1
DENND2A
PDCD4-AS1
AL022069.2
AC051619.5
AL138752.2
TMEM192
AC027307.3
AL022324.1
CDKN2AIPNL
TRPC7
CES4A
CPB2-AS1
LINC01750
MYO18B
LINC00515
AL731684.1
AC021752.1
ZNF658B
AP003486.1
AL135999.3
ENPP2
GADD45G
PROX2
POU6F2
LINC01765
HCN4
DPF1
PHACTR1
Z83847.1
OTULINL
ELL2
SLC25A14

RXRG
AC023830.3
LINC01554
SGK3
FGFR1
AC112184.1
MBLAC1
NRIP2
DDC
AL590787.1
SARDH
AL121768.1
AL035250.2
AL121758.1
GPRIN3
PIGP
FGF12-AS2
PAUPAR
PARM1
AC004803.1
MAFB
AL021396.1
AC107398.3
AC128687.2
AC048351.1
TPI1P2
CTNND2
PGR
AL353719.1
RN7SL268P
FAM163A
AC008752.2
AL162412.1
AMDHD1
LINGO2
SCN8A
RAI2
CLEC3B
SLC36A4
PRR18
MCOLN3
FNDC5
BRINP1
PCDH10

POU6F2-AS1
AL442128.2
AL163051.1
DAO
FAM107A
BLMH
NCOA5
PYY2
AL133320.1
AC026367.1
KHDRBS2-OT
LINC02001
AL160286.2
ZNF14
IGLL3P
KRT222
ENO1-AS1
C3orf33
MANSC4
FLJ37453
AC006942.1
AC010624.3
AC012603.1
AP5S1
AC025211.1
PGBD1
AL356740.1
AF274858.1
PANO1
KRT33A
CYP2U1
AL158163.1
AC091860.2
AC072061.1
AC020928.1
AC012313.10
CERKL
ASCL1
LINC01091
RNF150
CDK2AP2P2
FAM201B
AC011487.1
AC097468.2

TRPC7-AS1
CFAP161
THAP7
AC087501.4
DLG2
TMEM72
AL079301.1
PIK3CD-AS2
SLC4A10
GNG4
ATP5PBP5
AP001627.1
RAB11FIP3
DIRAS3
CFAP70
ARHGEF9
MIR600HG
DUX4L27
AL022329.1
BX649601.1
C10orf53
KDM8
PEBP4
AC016257.1
AMACR
USP41
NPHS1
CA10
AC073508.3
ZNF594
LINC00266-1
NDRG4
MOCS1
AL391069.3
SDCBP2-AS1
AC008806.1
AC087683.1
LRRN2
SIDT2
FEV
AQP4
NPM2
DNAJC30
GNAS

LRCH2
MESTIT1
ZFP82
HECW2
FAM174A
HMGB3P32
MAPK4
AP001972.5
RPL12P47
TSPOAP1
MDP1
FBXL21
CCDC38
KCNJ2
AC004696.1
SCARNA11
SLC35F1
AC244669.2
AC026801.2
AL353743.3
BBS5
LINC01779
AL138960.1
AC004540.2
KCNK17
WWOX
AC063979.2
SERPINF2
TUSC8
AC092667.1
AC127024.3
C14orf178
AL359853.1
R3HCC1
KIRREL2
P2RX5-TAX1BP3
AC104232.1
AC008629.1
CXorf67
AC244035.1
C8orf48
TUBB1
FAM216A
AC087623.3

FAM19A4
VPS33A
ZSWIM5
THTPA
LMO1
ADCY2
8-Mar
ADD2
ILF3-DT
SIK2
RPS6KA6
AC005899.3
AC025048.2
AC004825.2
SEPT7-AS1
LINC00888
EFCAB6
AC083964.2
AC026748.1
CITED2
AL355432.1
AC079949.1
LCMT1-AS1
LRRC24
AL356489.1
AP001160.3
AL035427.1
AL731566.2
RNF167
FAM169A
IRS3P
SNAI3-AS1
KLLN
PRPS1
SYN1
ALKBH5
WIPF3
NXPE3
SHANK3
AC004543.1
SEMA3G
CA11
AC005410.2
NSMCE3

MIS18A-AS1
EFCAB12
GNB3
JADE1
AP001533.1
MAPK11
ZNF607
TMTC1
RFPL1S
ID4
AC108704.1
MED31
C17orf75
AL390783.1
TBKBP1
ZNF18
AC074351.1
ZNF625
ATP5F1A
SPECC1L
AC091965.4
PRRT1
RIMBP3
NLGN1
AC053527.1
AL136531.1
OXGR1
CORO2B
CDC37L1-DT
AOX1
FLJ38576
ZNF710-AS1
CEP83-DT
AC008250.1
TSPAN19
LINC01952
AL358472.2
DGCR6
ATP8B2
BRF1
SMDT1
SCGB1D5P
PNMA8B
RNF212

AL360295.1
AC073911.3
JAZF1-AS1
INKA2-AS1
LINC01534
AL691515.1
PEBP1
AL109947.1
AC112236.2
ZNF396
LINC01985
CHGA
POTEI
DAB1
TNS2
CFAP99
SYS1-DBNDD2
U62317.3
AC015689.1
AC124319.2
SGSM2
GRM7-AS3
AL035420.3
AL137784.3
GTF2IRD1P1
KCNT2
CADM1
AL022328.4
AL079343.1
CHST8
RF00066
AC092296.2
TMSB15B
MOAP1
USH2A
CNBD2
POTEJ
ZNF559
AC104063.1
AC012640.5
CCDC96
C1orf194
AL109811.3
BTF3P14

FBXL17
AC004221.1
ZCCHC3
UNC119B
PWAR6
YIPF7
AC011450.1
AC127024.6
TXNL4A
AC092535.1
ANKRD46
LINC01664
FSD1L
LINC02226
GPR173
TBC1D9
SMPD1
C1QTNF4
LINC02251
ENAM
GSTM2
AC007406.5
AL590399.4
RASGRF1
EVL
AL133279.1
MAPK12
AC068620.1
ARHGEF26
AC016582.1
AL356019.2
AC002470.1
SSTR1
LMO3
U47924.2
DPY19L2P2
EFL1P1
CCDC87
MTMR3
AL355297.4
GPR158-AS1
AC099568.2
AC124303.2
AP004609.3

PDK4
AC084036.1
UCK1
NKAPP1
AC079610.3
RUNDC3A-AS1
METTL21EP
AKAIN1
FAM237A
ANKRD36BP2
RAP1GAP2
LRRC73
LINC00562
UOX
CALM1
PDE2A
TSHB
AC123768.3
AC025284.1
FAM47E
AOX3P
DCX
IKZF4
AC099684.2
PAPPA2
SHANK1
AC005911.1
AC015909.5
NUDT9P1
TMEM132B
EMCN
ABCA5
LIFR
AC034228.2
PKIG
AQP7
RN7SL305P
CIDEB
HMGB1P44
SERPIND1
NEFM
MRLN
AC124242.1
ZNF382

AP001496.2
AC061961.1
CSRNP3
AP001781.1
FXN
FFAR1
SLITRK2
NSG2
VN2R19P
LINC01567
GOLGA7B
TTYH2
AC010300.1
AC016907.2
ALOX12P1
APBA1
ARVCF
AC009088.1
AC074029.4
ARG2
CRADD
AC138207.3
OGFOD3
AC011444.3
FAM172A
ZNF876P
SAPCD1-AS1
PHYHIPL
KCNA2
AP003072.4
AMZ2P1
ELOVL2
AC087500.2
RN7SL693P
RFX2
AC024361.2
AC090993.1
HYDIN
AC079949.2
CYB561D1
SNAI1P1
AL021997.2
KIF3A
FGF12-AS3

GPR6
PRODH2
SNORD113-3
AC105383.1
FUNDC2
MAP1B
AC138356.1
CCDC65
GABARAP
AL080250.1
RPS6KL1
ZNF625-ZNF20
MRM3
TIGD6
GRK3
ATP6V0A1
GPR42
CUBN
ARHGAP19-SLIT1
SH3BGR
DCDC1
GGA2
AC035140.1
AC110079.2
AL359317.2
GRK1
PCYOX1L
GDF9
ADORA2A
MIR4488
GNRH2
KBTBD7
AC066612.2
STC2
LCMT2
NKAPL
AC092335.1
AL121932.1
AC091965.1
AL162231.1
MIR3682
ZNF775
RPL3
MED12L

GALNT11
AC105020.6
KCNC2
AC079760.1
ADAMTS18
SLC30A4
SRRM3
AC004882.1
KIZ
SLITRK1
ECHDC3
AF127577.5
PNPLA7
TCTA
AC119751.3
H1FNT
AC254562.3
AL138820.1
SNHG14
AL589739.1
CHST9
TAL1
AC024587.2
TMED6
NAA20
AC005775.1
NPAS4
LINC00533
CFAP65
SEPHS1P1
PMM1
DPH5
AC015819.2
FLJ37035
BRS3
C6orf118
CTBP2P6
LINC02044
RN7SL417P
GOLGA8M
AC009560.1
SEPT7P9
AC005593.1
PDCD4

Z97055.2
IGFBPL1
FOXRED2
AC090921.1
ZNF10
A2M-AS1
GNAS-AS1
CACNA1I
RN7SL121P
AC011445.1
AC079385.2
FLJ31104
TUSC3
STAT4
LINC01998
ZNF253
AC135507.1
SLC24A5
LINC02082
WRB
PTPRS
PRKAR2A-AS1
AC005393.1
BRSK1
AC011933.3
RADIL
AC024580.2
DIRAS2
MCF2L
DPY19L2
CCDC158
NPM1P47
SMIM10L2B
ELP1
AL591501.1
AL391261.4
MBLAC2
ZNF682
LINC00535
AC092634.3
AC090241.2
C17orf100
SPAG8
MMD2

	AC090531.1	
	AP005131.7	
	ATOH8	
	FRMPD1	
	AC012213.2	
	FOXN4	
	AC011511.1	
	AL133279.3	
	AC004147.4	
	MCMDC2	
	G6PC3	
	LINC01901	
	AL512408.1	
	Z95115.1	
	AC139100.1	
	CRB1	
	COPS4	
	DHRS2	
	TMEM61	
	LIN7B	
	AC107464.1	
	ISCU	
	UBOX5	
	FBXO9	
	RN7SL771P	
	SNORD115-45	
	AC068338.3	
	AL118558.3	
	FLG	
	CTTNBP2	
	GIN1	
	STX18-AS1	
	AC245140.1	
	MTERF2	
	RAB6B	
IGF2BP3	HSH2D	Positive
	AC023347.1	
	IPO8	
	KRT8P25	
	FDFT1	
	AC092569.1	
	EVX1-AS	
	KCNK5	
	SALRNA3	

LINC01068
PRRC2B
AC090151.1
AC106876.1
CRTC2
RRP9
PRPF38AP1
RNF207
DNAJC19P5
AC093001.1
AC092117.2
CTSH
GRID2
SENP5
AC124283.3
ALG1
RAB32
ERG28
LCN2
AC079768.1
SWT1
AL158824.1
UST-AS1
BCAR1
AC005481.1
AC026410.1
LINC00922
CIAPIN1P
AC026333.3
TFF2
RF00406
OR7E25P
AL365217.1
TAS2R13
PDGFB
CEACAM22P
RNA5SP73
ELFN1-AS1
HLA-C
SLC9C1
FNBP4
AC073593.1
FGF20
AL356235.1

SLC9A3
NBPF14
UBAP2
LINC01127
AC004832.5
AC016745.2
MARCO
AF131216.1
POLR3E
SLC25A39P1
LRRFIP2
LAMA4
TPT1P11
MPRIP
KRT5
AC245100.7
PSMG2
AL359740.1
MIR4706
NRARP
AC002383.1
OR4D12P
AC005546.1
AC007919.1
TTC33
CTSC
COQ10BP2
SLC26A5
AC020922.1
RSBN1L
MIR4525
RNU1-106P
UBQLN2
ERH
ZNF880
TGFBRAP1
LINC01271
AL358115.1
IDO1
NPM1P38
PRDX3
RPS3P2
RCN1
NME1-NME2

SOCS5P2
LGALS7
GBA
RNU6-1078P
AL157398.1
PPIP5K2
AP005329.2
BET1
AL035401.1
MMAA
HOXD11
YWHAEP1
AC010186.3
AGBL1-AS1
AP006193.1
LCA5
HNRNPCP8
AC020909.1
AC073367.1
PSMD3
BRMS1L
TMEM256P2
MIR3617
ZNF646
ESRRA
FAM160A1-DT
GLRA4
PPP1CC
AC023157.3
AC105118.1
PRKAA2
AC087311.1
AC019109.1
SELENOTP1
TTC17
CLRN3
CHST15
HNRNPA3P7
LINC02516
MIR3155A
PPFIA4
PLEKHA8P1
AC024937.1
LYRM4-AS1

ANKRD36
PIPSL
CTSK
AC097523.2
STIM1
AC009237.1
SMIM34B
AL121612.1
RPL32P35
NDUFB4P4
RNA5SP273
CFL1P2
RNU6-91P
FTH1
ANGPTL2
AC027315.1
SLC7A11-AS1
FEN1
CCT7
SNRPCP4
IRF2BP2
XRCC1
GPA33
AC005392.3
NADK
AL356801.1
MIR6512
GDF5OS
BCLAF1P1
AC078845.1
AC020633.1
SCIN
MEX3B
HRCT1
MIR4254
AC145146.1
SIN3A
AC009313.2
LINC00974
AC024581.1
MID1
SNX25P1
AC093668.2
AL022344.2

ALDH1A3
OR1J4
TMPRSS11F
RNA5SP435
CA9
RABGAP1
CALM3
ZNF462
IRF3
AC008752.1
AC020978.5
GTF3C4
ATP5PDP4
ZNF223
XXYLT1-AS2
AL590240.1
SHLD2P2
VTA1P1
LUCAT1
HTR1B
AC011676.2
AC012184.2
FAM72C
PTMAP1
BST1
HDAC2
GALNT1
NBAT1
FANCL
OGA
AC096656.1
LRP11
KRT16P6
CPNE1
DHRS7
BNIP3P4
OPHN1
TMEM164
AL390729.1
GGH
MRPS36P2
MIR151A
SOX11
AC025165.3

PAX8
BORCS8-MEF2B
GORASP1
RPS26P41
DIABLO
AL445490.1
AP001931.1
MCCC2
AC025419.1
HIST2H3D
NEU2
LINC01833
SMARCAL1
MIR6889
STOML3
OR7E122P
ROR1
FLAD1
HPSE
AC087439.1
LYAR
AL121761.1
AC023813.2
AL512353.3
PXDNL
AKR1C3
AC019226.2
RF00575
PLEKHM1P1
SIPA1L3
AC092647.2
PJA1
CRKL
OR5M6P
AC008782.1
ACTG1P3
TMEM132A
CCNL1
BLVRA
CYLC1
PP2D1
PCNPP3
GAREM1
ZFAND3

C1QTNF12
HADHA
DUSP9
CCDC18
RNU6-935P
ZNF485
IQCK
PFKFB3
AL132780.2
PTPDC1
AC008687.1
TSPO
RPP25
NEPNP
C7orf57
TEX13B
LINC02492
PGAM5
UPK3B
VPS41
AC007362.1
ZNF621
POLA2
C1orf131
AC040970.1
YY1AP1
AC244093.5
TMEM47
AKAP5
LRCH4
AC073325.2
MND1
RN7SL377P
AL356274.1
DSE
NKAIN4
PPFIBP2
AL121820.1
TSPAN17
FDPSP8
FAM76B
AC103705.1
AC068305.1
KRT3

TGFB1I1
AC129926.1
PMM2P1
AL360268.2
AC105429.1
HIST1H3C
MROH7
GRPEL1
ACRV1
LINC02142
CYP24A1
TNS3
HMGN1P37
CASC9
PAICSP6
TMEM69
RPL22P24
PCGF1
GAPDHP14
AC021066.1
AL138752.1
CYP2G1P
AC243773.1
DCAF6
ATP5F1AP10
PTP4A1
OST4
TAF4B
AC084783.1
COMP
MAPKAPK3
CCDC138
RAI1
AC079804.2
PCNX3
DUSP14
TMEM189-UBE2V1
HAPLN1
ADIPOR2
AC007318.1
AL356421.2
AP003478.1
UGT1A8
AL080312.1

ACTBP1
VENTXP3
TSPAN13
TIMM9P3
SNRPE
DHRS1
AL591845.1
NUAK1
KIAA1143
TOMM20P4
ZNF664
PDPK2P
AL354707.1
PAPOLG
SLC30A9
NFKBIZ
AC003070.1
ALKBH8
GPR160
AC016909.2
CXorf49
SOX7
AC106864.1
NKX3-2
AC087575.1
ZMIZ2
TRIM32
THEM6
GEMIN2
SNORD65C
TMSB4XP1
RAP1BP3
AC091588.3
RNA5SP351
INSC
AC004870.4
PIGC
BUD13P1
AC022336.1
LINC01106
TRBJ2-1
HIBCH
SPON2
AL731556.1

ZNF469
LINC01673
UBE2Q1
MXI1
SMARCA5
PSMA4
SMG1P5
THEGL
CATIP-AS2
CISD2
EMC3-AS1
METTL6
GADD45A
E2F5
AC073316.2
AL121772.1
ACTR3P2
FOXC2-AS1
MIR3149
SAAL1
AL451142.2
NIFKP2
FOPNL
AL162511.1
TXNP7
AC008277.1
TIMM8A
LINC01980
AC068491.2
PFDN2
BBC3
MTND4P26
AC024580.1
AL353804.2
MIR3680-1
HIST1H1D
IKBIP
AC069542.1
RNF5
BAIAP2L2
NOTCH3
ATXN7
BX293995.1
AL390728.4

LATS1
AP001282.1
RNU6ATAC22P
AC063962.1
POLR2K
MTERF1
BTF3L4
EYA3
LINC01338
LINC01145
METAP2
HCG4P8
AC093292.1
AL139022.1
PTPRG
PSENEN
FBP1
RPL23AP39
RP1
RPL23AP15
CCDC114
ONECUT2
AC108136.1
HSP90AB2P
FOXJ2
EIF2AK4
SART3
OR7E117P
UGT1A2P
HSPA9P1
FAM110A
MIR6876
PLEKHA2
ISCA1P4
SPIRE2
MMP1
RPA4
FBXL13
AC011352.3
AC073913.2
DMBT1
RNY3P12
LINC00326
HASPIN

UBA52P7
TSC22D1
TBC1D3H
ZFPM2
AC009927.1
CCL20
TOP1MT
DPF2
AC098476.1
CAMK2G
AP005057.1
SOCS5P4
CDK2AP2
BOK
C1GALT1C1
TGM4
IFI35
PAN3
MRPL47
INPP5A
AL118558.2
FDPS
MICALL2
CST2
AC034206.1
MIR1278
CAVIN4
RNU6-752P
IFIT5
SMIM3
AC117498.3
AC009093.3
MUC12
GAPDHP26
TFCP2
AL359075.1
LINC01220
MT-TW
RN7SL154P
NRG3
AC007106.1
MRPS30
WDR45B
AC087276.2

ATXN7L1
MED15
AP003062.1
FAM72D
PPIAL4A
LINC01844
AC100839.1
ANKUB1
ZDHHC6
AL034349.1
GAPDHP72
AC002525.1
RF00156
AC136475.10
MRPL13
AC092112.1
SPC24
PCMTD1
TUG1
ACTG1P19
TRMT1L
AC139720.1
TMC6
ANXA7
AJ271736.1
PCDHGA8
AC115618.2
ADNP2
RN7SL15P
ZYX
IQCB2P
MIR6730
RCC2P1
SMAD1
COMMD7
RN7SL263P
AL450322.2
MATR3
SNAPC3
AC027313.1
AL451164.2
AL442125.2
CHAF1A
CASP1P1

FHDC1
AC140479.1
RNA5SP197
AL008638.3
KCNMB2-AS1
FBXW11P1
SULT1C2
FAM168A
NR2F1-AS1
NBPF15
MPPE1
TFB2M
AC084757.1
TM4SF20
SNX18P10
UBE2D2
AC244033.1
RN7SL541P
AP2M1
FBXL19
AL353593.2
AL031726.1
MIR4645
FAM221A
XKR5
AC104389.4
GPR132
CHEK2
AL359880.1
AC008456.1
PDCL2
MMP3
GIMAP2
PUF60
HMGCS2
PRELID3BP4
NCOA4
HECW1
CBLL1
AC104819.1
SLC16A13
HMGB1P20
AC093281.2
AC135050.5

RN7SL204P
ZNF280C
KHDC4
AL512356.1
AC009132.1
NEBL
CCDC14
BLM
NCBP1
C9orf152
TMEM45A
C15orf62
WFDC11
AL445647.1
RBM45
APBB2
AC021678.1
NIP7P2
APCDD1L
LINC02416
RAB5C
ANKRD13C
AC026117.1
NTAN1P1
SNRPCP16
PALLD
RNU6-296P
NAT1
AL590139.1
AC005261.4
AC015849.5
USP3
ING1
AC093117.1
ROBO2P1
RF02132
AL358934.1
AC114814.2
MIR4536-2
NCOR1P2
AL078595.3
NDUFS5P1
ZNF134
STARD4

CDC123
PIGV
DGAT1
ODAPH
CEP152
AC027808.1
CTSB
HINT1P2
PHBP16
SMIM14
ANKRD12
CDC42EP3
OR7E102P
AC069200.1
EIF4A3
KAT6A
AC108479.2
RF01974
PCDHB10
SCARNA6
TREM1
AC027031.2
RPS27AP17
POLR2H
OR5E1P
TMEM179B
MDM2
AL391903.1
MTAP
MED30
TMBIM7P
SRSF3
DEGS1
AC099482.1
VPS36
PIGHP1
AC120042.1
LINC00858
LRRC3
RPL22P11
ACTR10
AC093844.1
ARL14EPL
MAP7D1

CDK14
ZNF738
AP000526.1
AC079466.1
Z83851.1
AC138028.2
AC008403.1
AL449363.1
Z98752.3
H2BFS
MRPS5
SNORD82
NT5C1B
AL929236.1
PHKA1
MIR6749
FAM192BP
ITPKA
AP001029.3
OR7E91P
SLAMF9
RANBP10
AL445531.1
HIST1H4E
MYSM1
RNU6ATAC12P
RNU6-586P
MTND4P28
AC024224.1
AC007274.3
LINC02472
AP001978.1
AL137190.1
AC006987.4
AC112498.1
RPL12P3
FNIP2
AEBP1
CEACAM18
MYOSLID
ZNF311
BRPF3
KIRREL1
RASAL2-AS1

RPL37P18
LRRK1
ARMCX5
PATE1
VCPKMT
AC013652.2
HMGB1P22
ZNF507
UBA52P8
AC078778.2
KRT18P7
MGAT5
PIN4P1
KRT84
SAMD13
OSTCP2
TMEM214
MRPS16P3
AP001453.2
FKBP10
CCDC70
ZNF7
APOBEC3C
AL354766.2
MNS1
USP8
MED13
AC040169.3
RPRD2
RNU6-1048P
LCMT1-AS2
DDX59
CCDC190
AC004835.1
FSTL1
FOXP1
COX6B1P6
AL133412.1
HNRNPA3P10
TET1
FAM86HP
AHCY
KRT18P42
SPACA4

ZNF283
AC010834.3
MIR6829
EIF3B
BRPF1
KCND2
AP000866.3
OMP
ARHGAP29
MYL12BP2
C11orf42
ZNF669
MSC-AS1
AL109933.2
AC012363.1
AP001486.3
AC108463.3
TFCP2L1
RAP1AP
AP003390.2
AC022858.1
AL390719.1
ZMYM6
BTN2A1
ARHGEF35
AC007599.2
ATG5
RPS10-NUDT3
AGO4
AC245100.5
ARHGEF9-IT1
AC024909.2
AC019080.5
GATA3
LINC02298
RPL17P11
MYC
EIF3E
RCOR2
OPN5
ALG10B
MAD2L1BP
AC019227.1
AC022150.2

DIS3L2
AC002401.4
SNORD6
AC007547.1
RPS26P49
FGF23
NCBP2-AS1
TFE3
FLJ16779
AC243972.2
RN7SL126P
TEC
ASS1P11
AC092669.1
AC069547.1
C1orf43
TUBBP2
AC010522.1
DNAJC22
NOP14
ZNF300
AL021920.2
AP000357.1
SNX5
SLC25A32
AC009803.3
AC024267.4
LLPHP2
ADAM12
LINC00904
NBAS
AC090502.1
BTNL10
TMEM51-AS1
HTRA3
AIMP1
LINC01561
PURB
OFD1P17
ITSN1
LETM2
AC243919.1
NRBP2
TPRKB

AMFR
FHOD3
UBE2N
TMEM248
MIR331
AC135586.1
CYP26A1
MYRF
DDX18P1
CCDC153
CARD19
LEMD3
ATG9B
CDC25B
ARPP19
AC044810.2
AC092115.1
TMEM183B
AC010328.3
MICC
HYPM
CYBRD1
AL033523.1
TINAG
C6orf141
MRGPRX3
AC008109.1
ATP6V0E1P1
RSRC2
USP46
DOCK6
MAP3K14-AS1
PLEKHM3
AL731563.2
AC004486.1
BRD7P2
MBD5
RPL36AP51
MEPE
TULP3
AP000949.1
AC048344.4
SLC24A2
LINC01556

LGALS9
AC007992.2
FEM1B
AC117383.1
GOLPH3
NBPF10
AF230666.1
DNAJC9
NUP153
SYF2
FHAD1
SYT8
AC007272.1
AL596247.1
LHX1-DT
GOLGA2
KRT8P33
BPGM
AC036222.1
MSN
JADE3
SLC44A5
NEPRO
UTP20
RNF114
RNF123
KDELR1
AC015802.1
CTDSP1
AC020915.1
CHSY3
RNU6-301P
RN7SL255P
TCF3
PPP2R2DP1
RNY4P17
RPS20P33
H2AFZ
RNU6-766P
AC073651.1
DDAH1
TXNIP
CAPNS1
AC239802.1

MTCO2P17
LINC01399
APOL2
AL772337.1
AC008700.1
AL080285.1
TWIST1
UBE2CP2
DHDH
AC009269.5
AL031717.1
CYCSP39
AC092821.2
MAGOH
AC009365.2
RXFP4
HOXB2
TMEM150B
ANKS1A
KLHL18
AC025154.2
HSPA5
CREB3L3
CSNK1G1
DTYMK
HNRNPH3
CLASP1
NID2
PSMC1P2
AC093895.1
AL445623.1
RBBP4P2
AC010642.1
NFKB1
LHX1
BEST2
PRPF38A
HMGN1P11
RPS3AP38
RAB23
AL121895.2
CANT1
AL596223.2
APCDD1L-DT

RNU6-1206P
TMCO1
ELAVL1
AC131971.1
SAP18P3
TENT2
RNU6-1257P
TMEM184B
HIST2H2AA4
RN7SL380P
GJC2
KRT9
AL583808.1
AC019155.3
GDI2
LINC01322
MKLN1-AS
SUCO
HMCN1
AL445123.1
HMGN2P22
AC022613.3
AL583722.4
WDR72
AL583832.1
CALD1
TBC1D12
KLHL12
MTND4LP14
CHRNA5
KRT89P
CXorf49B
GLB1L2
SPCS2
CHIC2
LINC02280
BNIP3L
GABRP
RNU5A-2P
LINC01908
AP001432.1
TMPRSS11D
NINJ2
PHRF1

PUS10
TNFRSF14-AS1
CDK8P2
WSB2
AC073323.1
TDGP1
DAZAP2
AC099313.1
PRRC2A
YBX1P1
DNAJC21
VPS72
ZNF165
LINC02121
AC016027.5
CLCA4
CWF19L2
ZNF616
C20orf85
MIR3122
TMEM65
MARS
MIR4316
MPV17
CHAMP1
KRT8P21
C19orf54
PRSS33
NFKB2
NPM1P39
AL772337.3
ZNF613
RAPGEF2
PTGER2
AC005237.2
AC007842.1
MYH15
CYCSP44
CHD9
SUMO2P4
AL590428.1
HNRNPUL2
HAUS6P1
KRT18P1

DNAH14
FANK1
ARMC8
UBXN2A
SNORD46
CCDC115
CIAO1
LINC00942
LNPEP
FAR1P1
TACC3
AL591178.1
AC002401.1
USP38
AC084117.1
CD177
AC080023.1
LINC02085
FAM189B
AL513327.1
AP006621.4
KRT8P1
MIR1255A
CCDC58
SNX8
HNRNPKP1
SPDYA
PRKCA
AC016746.1
SNHG4
MTCO2P15
RN7SL748P
NFKBIB
AC079776.1
ACTG1
VPS8
ANAPC10
EIF3FP3
ATP1A1
AC023934.1
AREL1
AC099520.1
TMEM200A
MIR4252

PPIAP11
LRRC37A9P
RAB14
VAMP7
PRIM1
AC020891.1
KRT4
PITRM1
TIA1
TMEM9B
MFSD2B
FABP6
GRK2
AC108865.1
PSRC1
COPS8
OR7E62P
EPN3
KIAA1671
PLA2G2F
LOXL3
LINC01348
AL604028.2
MX1
PLCXD1
FP671120.1
ZBED4
SHB
AL731571.1
ZNF861P
PRRX1
LRBA
AL645939.3
LINC00456
PDE11A
LINC01940
RNU6-1102P
MTATP6P29
MIR31
GCA
ZBTB2
RNU6ATAC16P
C4orf33
SLC8B1

CACNG6
RABIF
B4GALT2
MIR593
AL389895.1
SORL1
AC011611.3
VPS29
RPL17P50
MTX1
APOL5
AC105219.1
AC012531.4
NFATC3
WNT5B
ARL5A
MIR4421
MFHAS1
TLR3
PPIAP51
AD000813.1
SPATA5L1
SNORD19
ZC3H13
RPTN
AC096741.1
SLC26A8
BLOC1S4
AL078621.1
PNPT1P1
LEMD1-AS1
MIR7856
AMER1
OR4A47
CD70
KRTCAP3
SPARC
SETD6P1
FRG2HP
MIR3133
SLPI
ENKUR
BCL6
SERBP1P5

SNORA3C
FAM98A
RAB8B
ZNF385C
FLJ31356
FAM3D
GTF2IP7
RNU6-677P
AC002472.1
LYPD2
AL442647.1
AC098613.1
PPP1CA
TNFAIP8
POR
RBM4
AAGAB
RPS6P8
AFTPH
AL512504.1
FCHO1
NANP
TRA2B
FLNC-AS1
ZNF608
C6orf99
MIPEP
DLX5
SERPINB3
ZNF761
ADGRF2
UBE3B
KRT18P39
FAM83G
TPI1P3
SSR3
SMIM36
AL512288.2
FP236315.2
OR7E33P
DYNC2H1
SAR1A
CDYL2
UBE2V1P9

CLIC4P1
LINC00518
SFRP2
RNU6-308P
PSMB10
SNRPEP10
IYD
UTP14C
AC008781.3
PARGP1
SPAG9
AC009271.1
AC083801.2
SUPT20H
MIR4742
PTPN23
LINC01929
BX470102.1
RPL34P22
AC008982.1
PSD4
PPP2R2C
AC073964.1
EPB41
RNU6-276P
FERP1
TESMIN
MAGEA10-MAGEA5
RNU6-155P
AP1S1
ZNF229
RNF219
LINC02408
MIR570
RNA5SP82
AC106738.2
RNY1P7
PRODH
AC110760.1
ATAD5
UBE2V1P2
AC112482.1
FNDC3CP
AC106798.1

AC018804.1
AC007536.1
ZNF552
AC092868.1
Z99289.1
AC008687.5
SKA2P1
HMGB3P2
SPINK5
GAPDHP67
ZKSCAN5
CFAP20
SIAE
SPACA6P-AS
KIAA0040
AC092803.1
RNF10P1
B3GALT5-AS1
TCAF1P1
AC138409.2
PABPC1P2
AC107016.1
ADO
EEA1
GATA4
RSL24D1P6
PRDM1
AL591178.2
LINC01981
PCDHB13
SLC30A5
E2F3P2
AC005537.1
MED13L
CUL5
ENPP7P7
PIEZO2
AC087761.1
CALM2P3
LINC01605
SLC25A15
TTC6
RPS26P52
SH3BP1

IGFL2
BIRC6-AS2
AC068134.2
RPL29P19
ATXN2
NR1D2
RF00569
HSPA1B
AGPAT1
TIMMDC1
ADAMTS7
AL031714.1
RNU4-24P
AC108751.4
FAM133B
AC009108.4
ESPNP
AC087667.1
AC092892.1
RPL5P2
AC009075.2
ARHGAP8
GRID2IP
TRIM8
EAPP
ALG3
MIR5588
AL031736.2
C11orf91
AP1M2
SNX13
AC093904.2
AC073585.1
RNU6-510P
TAS2R60
AC121757.1
AL353593.3
MIR4286
TUBGCP4
RGS16
TMEM158
LINC01941
CENPW
AC116347.1

VPS13D
RIC8A
APELA
HOXC6
AL158801.2
RPS6P7
RANBP2
PHKA1-AS1
RPL39L
SNX18
AC008622.2
OFCC1
QRICH1
LGALS3BP
CASK-AS1
AC004808.1
GHITM
AC040904.1
TRPV5
APEX2
SRSF1
AZI2
USP2-AS1
AKT2
SERBP1P1
CMIP
HHAT
LINC01592
NUS1
ASS1
MFSD2A
MRPS18A
BIRC7
MIR6728
RN7SL473P
DERL1
MFSD10
AC138123.2
AL132656.3
VN2R9P
RNF26
NRBP1
RNU6-356P
ZNF195

HIST1H2AJ
RN7SL44P
AC018641.1
ADPRHL2
RNA5SP124
AC007529.1
AC023078.7
UST
AC098592.1
KIF13A
RNU6-525P
SETD2
SLC38A6
TIMM23
AGR3
AC023421.2
FER1L6-AS1
ASS1P10
LINC02428
DUOXA2
DCAF5
RPL29P28
RNA5SP134
GOLM1
AL161935.2
HGS
ATP6V1D
COX6B1P2
LAMB2
CARD14
TPP2
AC110619.1
BUD31
NIFKP9
LRP1-AS
AC023449.2
AL035448.1
AC008443.3
NETO2
RPS2P32
RN7SL684P
AC112187.2
AC009302.1
AC010333.2

VRK3
LINC01096
CARNMT1
MRPL14
CLTC
ATP1B1P1
PSORS1C2
AC107072.2
TMSB4XP8
HAPLN3
AC008906.1
PKMP4
ZNF214
MTND4P15
AC024940.1
IQSEC2
PMS2P1
MUC5AC
TMX2
LDHAP1
SLC14A1
SPSB1
LINC01915
NUMA1
LGR6
SEPHS2
AL365356.1
XK
AC025030.1
CTSS
M6PR
TARS2
RNA5SP505
ACER3
HIST1H4PS1
MMP2
LINC00524
AP001020.2
RPUSD4
HEATR5B
UBE2Q2P2
FAM122C
MAP2K3
SMC1A

RNU6-45P
AC010542.1
AP000317.2
HLA-K
CACYBP
AC004160.1
AC005162.2
HOXC4
RRS1
PIGG
SNORD121A
KLK5
AC016877.2
HTR7
MAT2A
LINC01722
IFNAR1
HMGB3P17
SUCLG2P2
MIR6745
CWC27
ITSN2
LAMP3
MMD
MTREX
LINC00337
DBT
ZMYM4
NHS-AS1
NPM1P27
MIR193BHG
ANP32A
CICP12
DSN1
ISPD
AC004817.3
LINC02474
MTCL1
AC087645.2
HS3ST5
AL356124.1
CRYZL2P
HSBP1
MEF2D

MIR2117HG
RAB1C
RPS7P9
SWAP70
VTI1B
NUTM2A-AS1
HMGCR
AP3B1
AC132872.4
NPM1P32
IARS
MIR5009
MIR3978
AC083806.2
KRT127P
USP42
CBX1P2
ALG1L5P
RGS17P1
AC073592.10
AC083843.2
FER
RELB
IFITM9P
PPM1N
SFT2D1
AC011998.3
AC004691.1
AC005741.1
JKAMP
AC020658.6
PDPK1
CFAP45
AC079203.2
MCM3
AC108174.1
BCL3
GPR180
PLCD1
CHP2
GLMP
ZNF565
CCNT1
AC022164.1

SERPINB8
AC093166.1
ZNF670
FER1L6-AS2
VDAC1P10
RNU1-30P
ANXA4
MTND2P26
AC005041.2
SNRPA1
MYB
MSH3
IL20RB-AS1
HIST1H1E
AC097347.1
AC134312.5
AC245748.3
SH3BGRL2
AC124319.1
GBF1
MIR621
AC007673.1
AC066613.2
AC011193.2
CABLES2
RN7SL68P
AC011442.1
MYO16-AS1
DUS4L
AC022395.1
PPHLN1
UBAC2
AC099667.1
MXRA8
YY2
HOXC11
WDR82
AC010595.1
AATF
KANSL1L
AC007342.5
RANP2
SNORD67
AC080128.1

GPR153
RN7SL130P
TMSB4XP3
SERP1
METTL4
USP28
NOA1
ADM2
KRT8P3
HAUS6
POU5F1
SECISBP2L
CHPT1
AC000123.3
AC099063.2
UCN2
AC012613.1
RNF121
RIPK4
CEBPB
LINC01224
GAPDHP28
DNMT1
STX4
LINC01123
AC004870.3
AC073130.2
FGF11
TRADD
AC141586.4
BMP8B
RNF135
TLR9
LYSMD3
BCOR
AC079601.2
AL354984.3
BUB1B-PAK6
HMGA1P3
POLR1B
LINCR-0001
AC108463.2
C15orf39
ZFR

B4GALNT3
TMEM30A
POT1-AS1
LINC01301
RNF145
DTX2P1
AL450306.1
C2orf27A
RN7SKP223
AP001208.2
AC007992.1
AL391058.1
GDPD2
KRT14
SEC14L2
RN7SL834P
ZNF322
AC122710.2
GLMN
INPP1
NREP
ATP10A
SON
AL049840.1
GPR176
ALG1L6P
AC062037.2
VDAC1P6
AC106782.1
HOXB13
KIAA0391
NDUFA5P4
AL132656.4
PACERR
ZNF749
THEG
LYN
PSMC1P1
HSD17B6
MYO7B
LINC02189
AKAP13
AC084859.1
MTMR1

AC027176.1
AL355102.5
OSBPL9
SNORA16B
PAQR8
ACVR2A
LACC1
LINC01385
ZIC5
CORO1B
AL031777.1
DPP8
RNU6-73P
CLDN18
PLCE1-AS2
RN7SL589P
SNRPG
HECTD3
AC114811.2
AP2A1
ST5
Z97198.1
ZNF292
RNF10
AKAP11
SEC62
AL031670.1
PITRM1-AS1
AC113423.2
MAGEA8
YRDC
SVOPL
AL121657.1
THUMPD2
NKX3-1
ITFG1
KRT18P16
PCGF5
RPIA
MTCO3P29
AJ239321.1
AC133528.1
AC060765.1
ELK1

AL031708.1
XRCC2
RNASEH2A
AKR1E2
ADGRA3
FOXF2
DTX2P1-UPK3BP1-PMS2P11
FUT6
C16orf74
PDPR
CEP57
AC091179.2
SLC23A3
CDK11B
TMCO3
AC005020.1
AC011257.1
LAP3
ANO9
NOC2L
AP000842.3
CHORDC1
HSPE1P26
AC005479.1
PWWP2A
PABPC1P10
B3GALNT2
ATF6
AGL
AC010325.1
RARB
RAB35
PPM1B
METTL7B
AL139260.2
C5orf34
LUM
AC007906.1
AC006058.1
FAM198B
MOB3B
AC092611.3
ADAM28

MIR4427
THUMPD1
CACNB1
ALG13-AS1
NAP1L4
AC004461.1
AC007998.4
AC233280.2
AC009948.4
TMSB4X
EIF4E
AC005255.1
PLCH1
AL109983.1
BX322234.1
WIF1
PTAR1
AL590627.1
ICMT
SCYL3
TICAM1
MTAPP2
STAT2
RN7SKP90
PEX2
AC006001.1
SLC6A9
SUSD1
Z98257.1
AL451142.1
AC099518.2
MIR205HG
MCUR1
USP7
RNPS1P1
AC015712.1
ELK4
AC073529.1
DTWD2
FGFR1OP2
SYNJ2
IFT52
UMPS
SETDB1

AC039056.2
AL353150.1
ARHGAP1
CTPS1
AL512605.1
KMT2A
TBRG4
AC121154.1
AC234782.4
FANCE
CFDP1
LCN1P1
AC244154.1
SNTN
AL583810.3
CCT8
PSME2
MAB21L3
BDP1
AC110588.1
NDRG1
AC022819.1
HMG20A
AGTRAP
LINC02320
LINC01500
AP001350.1
EIF1AX-AS1
VNN1
AC018638.8
GTF3C2-AS1
MTATP6P15
MYORG
RF00264
GMPR2
MIR6740
AC125611.3
PIWIL4
HSPE1P7
RNU6-957P
AC018714.1
AC239584.1
AC010261.1
PHACTR2P1

ZNF823
TARDBP
TRPS1
RASSF10
AC012594.1
BCLAF1P2
RNU6-438P
AC135279.1
AC068631.1
HSPE1P2
KRBOX1
NUDT15
AC103957.1
AC027801.3
RNU6ATAC38P
AC112721.2
LPIN3
DAW1
ATP11C
RNA5SP345
C1orf174
RNA5SP97
DBNDD1
AC104966.1
AC125494.1
AC007388.1
AC116424.1
MESTP1
MIR210HG
AC009093.4
SLC9A7
ST6GALNAC1
AL358913.1
RIMKLBP2
FRMD8P1
AC106037.2
PDAP1
UBR2
AC005828.2
AC007115.1
CNTNAP3
AC096751.1
RNA5SP75
MIR551A

AL592114.3
AC132803.1
AC092810.3
ZNF687
IFNGR1
LINC02000
PPP2R5D
CLPX
ADK
HCAR2
AL035661.1
CLOCK
NIPBL
LINC02551
RNA5SP450
CEP295
CCNJL
KRT18P17
MYPN
AC005104.2
AC005392.2
AC013394.1
STAC
MARF1
AC027228.2
CCDC88A
LINC01594
PSME2P2
SNORD13P1
SLC9A6
AL021578.1
DPY19L1P1
AL606490.2
LIF
COL4A1
SETD7
HIST3H2A
RPS6KA2-IT1
NME6
WARS
AL021807.1
SERPINE1
NUS1P3
AC115522.1

AC006262.2
POLK
MRPL37
SNX19
ARHGAP5-AS1
AL357033.3
CDHR4
PSORS1C3
RPS29P16
AC098848.1
AL136084.2
C3orf58
AL596223.1
RNU6-1255P
CNTF
LINC02560
AL035420.2
HMGN1P13
GMCL1
KDSR
ZBTB12BP
PRKAR2A
LGALS9B
FBN3
CYP4F11
C17orf53
LINC01012
GNE
POLE3
LYPLA1P2
EDIL3
HIBADH
RARSP1
AC009154.1
PDGFRB
CLDN6
SETSIP
DNAAF2
ELOVL7
MYO1D
FNDC11
AC010531.2
FAM45BP
AC098798.1

HEPHL1
PDP2
AC008870.5
F2R
SLC6A1
ZMAT2
PRKCD
GREM1
AC009084.1
ANKRD9
NLRP3P1
PSMB3P2
HMGN1P30
AL023284.2
AC078860.1
HIST2H2BD
ABRAXAS2
ARNT
KRT87P
LINC00525
HEXIM1
HMGN1P8
BMP1
AC079684.1
SIGLEC15
SEPT10P1
RBBP5
OR10H1
PRSS23
ABCB7
RUSC1
UBE2V1P1
CHTF8
GPR107
LINC00880
AC005336.1
BID
DDA1
GUCY1B2
UVRAG
RNU7-45P
AKR1B10
ANKRD40
AP003500.1

KCNA7
ANKRD26P4
BOLA3P2
FAM208A
EIF4E2
UBLCP1
AL133371.1
FGD5-AS1
PCNA
ZFPL1
LEF1
TOR1AIP2
RTN4R
RALY
TUBBP10
TATDN2P2
EIF2S3B
AC118345.1
AC011333.1
AC073150.1
AL031666.2
IL10RB
ALG1L2
HNRNPA3P3
DCLRE1C
PVT1
SLC4A7
AC017101.1
RBAK
IFITM10
BSDC1
SRSF9
POMK
ZNF567
PPIH
KCTD14
CKS1BP3
RN7SL452P
RASSF6
NR2C1
LINC01691
AC136475.3
MICA
SMG7-AS1

AL132671.1
CLCN1
CCNT2
LINC01792
SETP22
AC112484.2
KRT8P39
LINC02122
PROSER2
CEP170P1
SSH1
MIR572
MIR5188
CENPH
AC004870.2
AC145285.4
MN1
MOGAT3
KCTD21
LINC02253
RPEP6
AP003123.1
RNU6-783P
RNU6-1307P
RN7SL703P
RN7SL379P
SNORD74B
MIR6078
AC093722.1
MTND6P2
OR4A41P
WWTR1
SUMO1P4
RNU6-1098P
RNU6-100P
EPHB2
CHSY1
TOMM70
ZNF792
EMP2
GSS
ATP5PBP7
NDUFAB1P1
LTBP2

TMEM237
CIR1
HIGD1AP1
HNRNPA3P6
AL035413.2
KRT81
PALM2-AKAP2
OR7E136P
AL033384.2
TNPO1
RTP2
SF3B4
SSPN
AL035411.1
CLIP2
LINC01764
NOD2
MIA-RAB4B
HSF2BP
AC099782.1
RMDN3
AL353583.1
DNM1P17
RIPOR1
G6PD
UTP11
MIR6810
KRT6C
HS3ST3A1
HIST1H2AM
AC091212.1
AL590240.3
LINC02300
TNFAIP1
AC023043.2
RPS27AP10
KMT5B
AC092919.2
HPS5
AP001453.3
ZNF160
PRDX3P1
BTBD1
HIST1H3E

MMP7
CMTM4
AC114402.1
CTBS
SBF2-AS1
OR51F5P
VDAC1P5
EBLN2
PXDN
AL133399.1
L2HGDH
ARSH
AC084026.3
TASP1
POLG
USP25
CLEC16A
LAMTOR3P2
UBBP5
AC098828.1
MIR595
CCDC82
SPATA20P1
LINC01524
SDCBPP1
CCAT2
DUSP4
RBM12B-AS1
LY6D
MIR4435-2
UBL4A
SLC5A6
MGAT4EP
AC007098.2
CENPO
TMEM171
RNA5SP37
AC243772.2
MTRF1LP2
DGAT2
SQOR
AL121790.1
AC004080.2
RNU6-927P

MYO3B
AL589743.4
NAPRT
ZDHHC18
AC106037.1
AOC1
TMEM200B
RAD50
PRTFDC1
TMEM38A
ANKRD40CL
AP000919.1
CXCL10
CDH17
ADAMTS2
AL078644.1
VSTM2L
UHRF1BP1
NOP10
AC078883.2
NPTX1
PDPN
SFMBT1
NCCRP1
TVP23B
YPEL5
AC117422.1
HIPK3
RNA5SP156
ADRA1B
BICDL2
MIR4258
VHL
ZBTB44
ZNF697
PPP2R5A
PIP5KL1
AL118506.1
TMEM185A
FANCF
AC068647.2
KRT8P18
RPL7AP2
AC090525.2

L34079.3
FLNA
CDK8
AC007991.2
ITGA5
KDM1B
AC239804.2
EIF3H
AC093525.4
MIR1254-1
URB2
CREBZF
CASP14
CNEP1R1
MEST
AC239803.1
LGALS17A
WWP2
IL32
SAP30L-AS1
CALHM3
LINC01967
AC068790.9
DUOXA1
SH3PXD2B
TMEM181
NAMPTP1
SNORD53B
PSMA7
TM6SF2
BLVRB
HNRNPABP1
LINC01356
AC013652.1
AP005432.1
CFAP300
MTND5P1
AC105020.1
MMGT1
AC112236.1
CMBL
LANCL1
DFFA
INO80

AL109954.2
AL158827.2
MSH6
PLA2G3
AC010551.1
NOTCH2NLA
OR1Q1
FEZF1
TMEM167A
RNU6-876P
NVL
KLHL6-AS1
PGM1
APP
AC244517.10
AC130456.7
S100A11P1
FAM198B-AS1
CCDC91
CFLAR-AS1
GATAD1
UBE2D3P2
RPL23AP55
NUDT4
KIR3DL3
MFAP3
CLDND1
AC078819.1
USP15
KCNE3
SLC29A3
POMP
CHD4
HSP90AB3P
AL513485.1
IREB2
PCSK9
RPS29P12
HCG4B
AKAP1
LARP1B
PAPOLA
ZSCAN32
CYP2W1

ATF7IP
C4orf19
CCT3
RNU2-16P
TRIM47
GPRC5D
SLC25A37
AP000879.2
AC245100.2
STX19
CEP104
INTS7
LINC01186
ZNF184
ICAM5
MIR1293
PLBD1-AS1
TP53BP1
AC138938.1
ANKRD18A
EME1
GNG5
AC007639.1
EIF4EBP2
AC093909.1
CD276
PNRC2P1
AC103681.1
KPNA2
BECN1
AC137579.1
LINC00165
NPBWR1
TRAIP
TRIM11
RAB18
AL022238.2
KRR1
GALNT12
KALRN
PLA2G4A
AC009486.1
ZNF239
NID1

NSDHL
LINC01907
H2AFY
BRD7
CLTB
KRT8P5
AC025539.1
ZNF410
UGGT2
KCTD3
ABCC11
BAZ1B
PELO
RNPS1
SPAG17
TUBGCP3
DSP
AC011747.1
MSL1
TWF2
AC008781.2
MRPL9
HDAC1P1
HSPA1A
RASSF9
LINC02262
TRIQK
PUDP
NUDT19P5
KRT8P41
ACTBP7
AL391121.1
PSMD5
CDC25A
LRFN4
MSANTD3-TMEFF1
AC010378.1
RNA5SP462
AC024592.2
MYO1C
Z98884.2
LINC00592
AC092757.2
AP003355.1

AC108010.1
MIR548AA1
TRIP6
MCRIP2P1
ADORA2B
MOGS
TRPV3
CTR9
HMGB1P11
ACTRT3
AP005136.2
EIF4EP3
SNRPCP19
HPRT1
AC007342.4
EMSY
LINC00222
PTK7
SRRM2
AC007996.1
LINC00482
C7orf69
RNU7-46P
AC067956.1
VIPAS39
TC2N
SOCS5P3
HSPA4
PHETA1
AC007382.1
AC004846.1
AC245884.3
SKP2
VPS26C
PNPLA8
ZBTB33
KRTAP4-17P
CD200R1L
YBX3
AC089983.1
TONSL-AS1
SPECC1
CTNNBIP1
PRPF18

AC092070.2
DIO2
HNRNPA3P12
AP000873.3
C8orf33
AC013410.1
DUOX2
PCDHGA10
SUV39H2
PRKD2
MIR6753
DHCR7
AC010976.1
AC099314.1
PCDHGC3
AL772284.1
HIST1H2BG
LINC01910
PTCSC3
GORAB
CXCL5
VPS50
AC110373.1
AC027119.1
EMP3
AL355076.1
RPS3AP53
CAMK2D
SHISA2
AK3P5
PEX11B
ZNF750
SLC39A2
POT1
AP001207.3
FMO9P
DCTN1
AC021192.1
CSNK2A3
AC090023.1
STX17
BLCAP
MARVELD1
MAPK8

MUC3A
TXNDC12
GNAQP1
CDCA3
ECE1
KCCAT333
LRP8
CKS1BP7
HMGB3P31
AL121895.1
UCK2
KRIT1
ZNF251
ANKRD17
PHLDA3
HOXA-AS3
NEK11
RN7SL12P
CREBBP
AP000560.1
AC092104.1
RHOA-IT1
ACTG1P20
PRR12
AC004987.2
PPP4R3A
ANKRD18CP
OSTC
SEMG1
AC108861.1
AGMAT
EYA2
ASPHD2
TPRG1L
KATNBL1P6
GGNBP2
BAALC-AS1
PTBP1P
CCT7P1
MME
AC090954.1
KLHL2P1
AC092338.2
POLR1A

U2AF2
FBN1
LDAH
FRS2
USP32
HMGN1P7
ZFP36L2
POFUT1
MED4-AS1
UGT1A3
MICB
LARS2
ZFAS1
ZMYND8
AC013714.1
LEO1
AC007215.1
PDE4C
AC010531.3
SEMA4C
AF127577.3
ACTBP11
DPH2
AC090114.3
MMS22L
AC138393.3
TRIP4
AC092168.2
AC096536.2
ZNF320
AC073107.1
URB1
ACKR4
CDC14B
SETP14
ERN2
DNAJB6
DSTNP2
AC093582.1
AC093865.1
LINC00964
RBMY2KP
AP001363.1
MTF2

ABCC13
CS
AL138789.1
RHOBTB1
AC024085.1
MEMO1
ACTBP2
MAP7
TMED10
AC012645.4
AL360014.1
FOXH1
DCP2
IFI44L
RDH13
MED8
MATN3
SP110
COPS7B
RANBP9
AL033384.1
PDCD5P2
AC092851.1
PA2G4
TNFAIP2
AC104134.1
AC132008.2
ATG7
ESM1
ZNF484
MARVELD2
AC138028.6
AC015923.1
AC016866.3
FAM111A-DT
WASF4P
DYNLL1
PPIL1
PAICSP4
ARID2
DHFR
AKIRIN1
PPP1R14BP3
USP33

AL512363.1
DACT1
SOX30
AC007279.1
CDC42P6
ANKRD1
PYGL
PLA2G4F
RNA5SP151
AF230666.2
RNF24
BTBD8
REG4
CDHR5
IFNWP19
GAPDHP65
AP001000.1
SPIN1
HYAL4
AC008649.2
SC5D
BOD1L1
ARL6IP5
AC108724.1
SLC37A1
AC022336.3
RNU6-704P
LINC02561
ODF2L
DDX52
ARL4A
VDAC1P1
PPIC
TMEM131
TMEM256P1
AL355102.2
YIPF6
ISG15
NFRKB
FKTN
LINC00343
IGF2R
AC004466.3
OR7E7P

RN7SL815P
YWHAQ
HOXA5
ANXA11
AC009262.1
CGGBP1
AC090709.1
ABCB9
HECA
MAP3K6
AP003068.2
PPP1R2P6
TMEM26
MORF4L1P1
MICE
RGS20
IGF2BP2-AS1
AC068299.1
MTND4P23
LRIG3
VAMP8
FXYD5
SDR42E1
RN7SL568P
TTI1
MIR633
PCSK7
AP001542.1
MIR4802
SMG5
LARS
LINC02432
TERF1
H2BFWT
AL034546.1
C7orf61
ATP8B3
TNFRSF1A
AC004923.4
AC138028.4
OGDH
AC068473.4
IBTK
NOG

PROM1
AC005726.2
AP000474.1
NAALADL2-AS3
AC091806.1
GASAL1
NRIP3
AC109460.2
LINC02411
PLA2G4D
KISS1
OR51C1P
U62317.1
SPG21
UBE3A
GJA9-MYCBP
ARL6IP1P2
MC5R
AP001434.1
AC012501.1
ODCP
SPPL2A
AC010768.2
ID1
DDIT4
RNU1-16P
CHD8
TGFBR2
ARL13B
GTDC1
AQP6
MEX3A
SYNCRIP
HSPD1P3
MDK
AP4E1
FAM91A3P
NONO
NDUFB3
TMCO4
AL136221.1
RPL7AP52
SNIP1
MTND5P28

CRYM-AS1
AC107959.3
SLC9A4
PPCS
PRM3
MIR5689HG
SUPT5H
RPL10P13
LINC02019
CXCL16
C6orf15
AC004551.1
HNRNPA1P73
NUP62
P3H2
RF00593
TYW1
MAST2
UPF2
ST8SIA6
AC091133.4
DDX58
TRIM56
MAML1
SLC22A20P
THAP9-AS1
MARS2
HENMT1
HMGN1P36
ELOC
DVL1
APOBEC1
HSF1
RC3H2
SULT1E1
GPR161
LYST-AS1
MOV10
GOPC
AC007533.1
PCDHGB1
RNU6-1175P
MFAP2
STK4

AC025183.2
LTN1
AL049747.1
TPM1
PGRMC1
SLC38A9
AC069499.1
SELENOT
MTND5P30
ZNF526
AL139407.1
AC012615.4
NECAP1P1
AP002761.1
SMIM31
CSTL1
RAB12
ZCCHC9
AC021106.3
MAPK1
MLLT10
LMCD1-AS1
EFNA1
HIST1H2BE
ISPD-AS1
DBNDD2
AL121753.1
TMEM106A
YWHAZP7
USP43
SPOCD1
CNPPD1
AC079804.1
AL365356.4
LINC02029
RBL2
PTPN2
AP001020.3
RNU6-702P
FAM111A
AC019072.1
STK17A
AC106782.2
AP000275.2

MBOAT7
UBTD2
TNFSF10
BRIX1
SLC2A12
SERPINB9
IGFL3
PHACTR3
AL645939.2
PLEKHG1
MAP3K1
MIR181A1HG
HMGB2
AP002414.1
AC011411.1
RPSAP13
FMNL2
AC108516.2
PCED1B
KDF1
MIR365A
AL365181.2
ATP6V1C1
PPP6R3
MAN2A1
NTN4
FAP
SVIL
AL079303.1
PDCD11
LINC02542
GNPTAB
GLO1
TM9SF2
AC113191.1
SMC3
AC079416.3
RNU7-48P
ILDR1
ARMC9
AC021127.1
RNA5SP219
CCRL2
MBIP

GTPBP1
PRB3
ICAM1
ARRDC3
TNFRSF10D
SYT12
HNRNPH2
OR2A7
AL035405.1
TBC1D3G
RPL17P10
BEND3P3
FAM192A
SUGCT
GALNT4
LRRC15
RNF13
RPL9P28
FEZF1-AS1
COA7
SAMMSON
MFGE8
AC007218.1
ZNF32-AS3
UGCG
HMGXB3
UTP23
ARHGEF16
AC005863.1
ERCC3
CDC23
SP6
EVI5
PLP2
C6orf222
AC093525.9
AL359922.2
SLC2A3
DNPEP
LIMS1-AS1
BMS1P7
SLC36A1
AC023794.5
SALL4P7

AL590006.1
PHF3
HNRNPAB
RNU6-668P
PFN1P4
CFLAR
CRYZ
OSTF1
SNX9
ZFHX3
ANOS1
DEDD
RNA5SP295
RN7SKP281
GNAQ
CRLF3
EEPD1
CUL2
RNU6-693P
GPR137B
AC007001.1
AC090673.1
MTPAP
AC007342.8
NKAPD1
HARS2
DUXAP8
CBLB
KREMEN2
ZP3
DHFRP1
ENOPH1
HEPH
PLEKHA6
AC004832.2
DUOX1
AC010533.1
AC006058.4
TLE3
AC007450.3
CP
ADH5
UAP1
AC112721.1

AC132192.1
MAPK1IP1L
CHD2
ATP6V1A
WISP1
AC135068.5
MIR3686
AC008695.1
RF02118
IMPACT
ERLIN2
ZNF528
CLIP4
RAB1B
FAM206A
PDIA3P1
CMAS
VRK1
C11orf44
MIR4470
AC008760.1
EIF4HP1
GTF3AP2
TOR1B
JPH1
ZMYND10-AS1
PLEKHF2
AC023818.1
AIFM2
AC126124.2
CD59
TMEM63A
FBXO22
FUBP3
USP34
PADI3
TRIM44
AP003119.3
SLC4A1AP
TSEN15
SMCO2
CDKN1A
SLC6A14
CRYZP1

THADA
FBXO3
OR7E1P
TRIM7
AC073610.3
AL663058.1
ANKRD18B
PPIAP57
PPIL4
CASC11
AL592156.1
WNK1
SP7
AC055811.4
CCP110
NBR1
RF00432
CATSPER2P1
SMPD4
WWC2
PSMD13
UBAP1
CDC45
ISM1
CCDC186
TP53I3
PACSIN3
LINC02051
AL645608.8
DBR1
AP001318.1
TNK2
OR7E128P
ATE1
CASP3
GANC
AC020916.2
MIR31HG
LAP3P2
ZNF766
LIPM
LINC00519
ZNF322P1
HNRNPA3P9

SEPT9
FAM83C
GRPEL2
ZCCHC4
LUZP2
AC007494.1
KIDINS220
TMEM54
RHPN1-AS1
AL121603.2
ARFGAP3
ALMS1
OPTC
SHOC2
VSIG2
CDK13
RPL7P14
TBX18
PTHLH
ECEL1P1
OR2W6P
AC036103.1
RNA5SP29
GGCT
RNF31
SMIM35
IFI44
EDC4
GNPAT
PPP1R14B
ACOT7
AC007899.1
RN7SKP266
DENND4C
VIL1
WISP3
CALB2
AC062020.1
AC108134.1
SNRPD1
RNF139
KRT16
TMEM92-AS1
WDR53

JAK1
CAMSAP1
AC092687.3
SLFN13
WASHC4
IL1RAPL2
AC015712.6
MTF1
HBEGF
MAP1LC3B
AC243960.5
NOX4
LINC01169
BCORL1
ZNF816-ZNF321P
SRSF7
KCP
RNU4-85P
BIK
NAMPT
AC009154.2
PPIA
SLC4A11
AC025576.2
PLA2G15
CARD18
PDLIM7
MIR3679
CA12
HAS2-AS1
EEF1E1P1
RNF11
RSU1
TRABD2A
TTF2
DNAJC14
AP000542.1
KRT18P33
FNDC1
MFN2
ARMT1
STK17B
RPN2
HUS1

ANXA5
FOXL2NB
SHMT2
TDRD7
ULBP3
PPP4C
KLHL23
CCDC148
MIR138-1
ATXN7L3
ATG12
AC027088.3
CEP192
SCDP1
ZNF649
KYAT3
CST1
AC124276.1
AC003006.1
MIA
PHKA1P1
PLCXD2-AS1
XPOTP1
ATP1B3-AS1
RASAL1
GSDMC
EIF3J
KLHL21
RNU6-282P
TMPRSS3
MFSD14A
AC068580.4
SRP72
PPP6R1
HHIP-AS1
AL355388.1
AL583722.3
MMP13
RAP1A
TAF1
PCDHGA9
PARD3
SPG11
MIR6870

AL663070.1
LIPK
ADAM15
AC092756.1
PHF20
AL591806.2
RN7SL192P
LINC02450
RNF4
TBILA
RNU1-91P
AL512656.1
AC124067.3
BRI3
IFNWP2
NABP1
AP000974.1
AL139147.1
TRUB1
ANXA10
PAQR3
IFI16
AC026471.6
MIR569
RNU4-23P
WDR36
RNU6-377P
ACER2
AL691447.2
KRT18P68
FAM149B1
APOBEC3B
HLA-F-AS1
FAM110C
RAF1
SNORA80E
ATXN1
PSME1
CDT1
NAA30
ZNF808
AP000870.1
ARID1A
DNAJB1

HLA-G
DDX47
ANKRD10-IT1
C16orf58
AL589745.1
LDHAL6B
EXOC5
TRPV4
LARP1P1
AC096721.1
MMP28
CYTH2
HTR5BP
SOCS5
RAPGEFL1
AC009163.1
MIS18A
CRYZL2P-SEC16B
SLCO3A1
NPM1
HARBI1
DENND6A
ZNF518A
AL354993.2
RBM44
MIR559
RAET1K
TP63
CDKN2A-DT
AC079070.1
TRIM10
KRT78
UNC93B1
TPI1
CGB5
GBE1
DQX1
CPT1A
LINC01270
IL15RA
RNA5SP123
MED6
ARF4P1
TMEM154

AC004923.1
EDN1
RTKN
AC011479.1
SMC5
AL450322.1
PDZD7
AC108727.1
TAF6
EEF1DP5
POLD4
AC091132.2
AC009237.9
DOCK1
MTCYBP21
CEP72
MIR608
CCZ1
SLBP
TMEM62
AEBP2
PTDSS1
SRD5A1
FYTTD1
AFG3L2
AC073957.3
GAPDHP44
SLFN12
CEMIP
PCNX4
POU2F1
FBXL18
XRRA1
CXCL11
MFSD13B
AC063976.2
FAHD2P1
YBX1
SKI
PATE2
DSC3
IRAK3
ADAP1
PLEKHH2

PHC2
BATF2
CHCHD3
AP000402.1
POLR2C
CYP2J2
FOXD2-AS1
LINC00511
ARHGAP26
TTC27
KDM7A
HIST3H2BB
NAA35
LITAF
AL138881.1
GINS2
KRT8P37
EBNA1BP2
PPP1R8
AL513123.1
TAF1D
LINC00944
AC107021.1
NPM1P26
TAS2R38
IGFL1
AC244230.1
MRPS6
WDR62
CDC42EP5
PTMAP8
MTND4P9
HIST2H2BC
PCMTD1P3
LIN28AP1
CPPED1
IER3
ITGBL1
PTPN20CP
ILF2
AC091057.4
KLHL7
AC095031.1
TMPRSS11E

AL356274.2
AC009041.4
YTHDF2P1
EFTUD2
ZNF695
STXBP3
ATAD2B
AC004817.4
TPI1P1
TBK1
HDAC7
GCKR
AC130456.3
AC007191.1
HILPDA
SLC7A1
RNU6ATAC18P
DDX46
FAM45A
CRYBG3
ATP8B1
MITD1
AC007014.2
AP005264.1
USO1
U52111.1
CSF2
AC087857.1
MIR1296
RNU6-808P
AL049697.1
KDM4A
AC027544.1
REL
BEST4
IL4R
CLCN3
GID8
AC008870.4
MAPRE1
AC067930.5
MLKL
OSBPL11
AP002478.1

IPPK
PI4K2B
MGAT3
AL731533.1
NDNF
VWA3B
SBK3
ANKLE2
PTPN11
AL035456.1
AC016877.3
PPP1R3D
RTF1
AL162431.3
CARD6
PLEKHG3
ZNF888
UGT1A12P
LY75-CD302
MIR6784
UMAD1
AP3M2
TIGD2
AC092964.1
USP18
AQP5
EPHB6
C12orf56
SLC6A8
HOMER1
E2F4
WDR76
EIF1AX
LGALS8
LGR5
ZFAND6
CLIC5
CDSN
MAFF
UBR1
TMTC3
ZNRF2
EGLN3-AS1
AC016735.1

NUTM2F
CEPT1
SPRY2
LCLAT1
AC128688.1
AC099342.1
AXIN1
TNFAIP6
PIBF1
XXYLT1-AS1
VPS9D1-AS1
UBE2B
ELMO3
ZNF277
FARSB
SORD2P
PLS1-AS1
AL359955.1
UQCRC2
LARP1
IRF2
BIRC3
HIF1A-AS2
AP001107.1
DTX2
CTAGE12P
CREB3L1
RPL12P44
Z93242.1
MTND6P21
AC010983.1
AC087241.4
AL583722.1
DNAAF3
ZNF365
RNPEP
GLI2
RFC5
AP003472.1
HIST1H2BF
AC090971.2
TNIP1
GDA
ECM1

MIR616
MRPL44
INTS6
AC104447.1
LRRIQ4
TMEM51
CDCA7
AP000696.1
POLR2B
TM9SF1
OACYLP
AC092368.3
AC018695.4
KANK4
AC009238.2
BMI1
COL6A3
API5
CLNS1A
HSP90B1
CGB8
SUPT16HP1
HIVEP2
ODR4
PPP4R3B
PQLC3
DRAM1
HIP1
HYOU1
AL035420.1
MSMO1
BACE2
AFDN
ALG8
SLC45A4
E2F6P4
PCNP
ORC1
GPR78
MTA3
ZNF827
HIST1H3B
ANKRD61
ZNF117

SENP1
AC004080.6
NRIP1
GDPD3
AL162258.1
AL121790.2
VWA2
EEF1DP3
B3GALT5
MIR222HG
AL356390.1
PITPNB
KAZN-AS1
ATP2C2-AS1
TMEM8A
MGST1
C3orf67
HIST1H4K
LZIC
MIR579
CCDC90B
FIRRE
IGSF9B
PARP4P2
TRAP1
RNU1-134P
SRPX2
APEX1
IL2RG
ZG16B
SPAST
ZSWIM4
AC079062.1
DAP3
KRT8P49
CALML4
CTSA
CENPQ
ZBTB12
FAM102B
KLHDC7B
SLC7A5
PKP1
PVR

GABRE
BNIP3P7
CDKN2A
USP9X
SOX21
ABHD5
CEP350
AC093908.1
ZNF680
AC093732.1
AMZ1
C14orf119
CAPZB
DNAJB14
AL442125.1
AC005632.1
VDAC1P11
STK35
KLF10
AC137834.1
AC092115.3
AL359555.1
EFL1
EIF1AXP1
GTF2F2
RNF6
AC087385.1
AK3P3
STEAP3-AS1
RPS5P2
CCNE1
DNAJC2
EPDR1
LINC02246
AC008967.1
NPTN
GET4
CPNE3
OCLM
AC006460.1
TBC1D15
CRIPT
TMPRSS2
AC108729.1

AC011472.5
AC090164.1
CHMP5
USP47
ZNF710
AC051619.2
AC233968.1
BBIP1P1
PITPNM1
NAF1
LNCSRLR
CHM
OGFRP1
USP31
ACACA
AL365436.2
HNRNPA1P45
LINC02454
MECR
OSBPL5
HSP90AA2P
SLC5A12
WFDC10B
AC091805.1
ZNF562
ERVMER34-1
SAMD12-AS1
RBM8A
FAM136A
GLRX2
LINC01801
AC022034.1
AC008687.6
ZEB1-AS1
AC006483.1
LINC01843
C2orf69
PIK3C2G
PPARD
XYLT1
NAGS
SLC41A2
TRMT61B
CYCS

MTND4P2
CCDC85C
MTHFD1P1
CDC42BPG
AL512274.1
CYP4F3
NOC3L
SLC52A2
AL360270.2
COTL1
CD274
LINC01593
AC091959.2
SDAD1
SCP2
PARP12
SALRNA1
CYP4A22-AS1
PARVA
ZNF341
AL031848.1
PBK
CEBPZOS
RB1
LINC02100
SEC24D
MTM1
BEAN1-AS1
IPO8P1
RNF103
CACNB3
RFFL
CCAR1
IL1A
RN7SL566P
CXADR
CTAGE15
KIAA1549L
STAU2
GPATCH1
EXOC4
MTA2
UFM1
HOXB-AS3

KRT18P38
MRPS22
SLC12A4
SP2-AS1
TOX3
FAM20B
AC105219.3
RN7SKP21
STN1
CCNJ
CCT4
ARPC3
AC022405.1
CLMN
AL136115.1
CDCA7L
TCEAL9
RN7SL431P
SMARCC2
ZNF611
PANCR
AL136116.2
AC131532.1
EEF2K
HK2P1
STRN4
DNAJA4
AP001610.2
EDNRA
TIAM2
SDHC
CHST11
ZNF341-AS1
AC005288.1
KIAA1191
NUDCD3
TRRAP
AKAP14
PAK4
S100A5
TMEM68
PPA1
NT5C3A
OR7E116P

PFDN4
PMS2
RPS15AP10
AATBC
CALR
IFITM1
LARP4B
MAPK15
PDC
EMP1
AC138150.2
HIST1H2BN
TANC2
CASP2
ANO7L1
LPIN1
AC108359.1
CDHR2
CTSZ
MEX3D
HOXC10
AC008114.1
ELOCP19
HP1BP3
AC004080.3
ADTRP
PDIA3
TRHDE-AS1
COL1A1
SDHD
COG4
MARCKSL1P1
HHLA2
GNA13
TRIM14
ZBTB34
GIPC1
ABHD11
E2F1
EPYC
KRT8P10
AC233699.1
AC022211.1
DDX3X

MED12
CSDE1
KBTBD12
MORC3
AC008740.1
FLII
EIF4EP2
AC099518.1
SRGAP2D
ZNF609
AC005828.6
AC007556.1
CDK16
AL139352.1
AC002367.1
RMC1
SLC9B2
BAG5
DLEU7-AS1
ASH1L
KATNBL1
BAZ2B
UBA52P6
TGFBI
CDK2AP1
SNRPGP2
MED20
MR1
TMEM67
NAPEPLD
AL034550.1
ARL2BP
CLIC4
AC099518.4
LPIN2
ZBTB38
EZH2
YTHDF1
UBC
BTN2A3P
RAD23A
AL390729.2
CLCF1
GLCE

MRPS31P1
CHAC2
SMC6
ZNF137P
RBM15
ACSF2
SIGLEC6
LRR1
CDC5L
TMEM161B
POSTN
TSTA3
RTN3
DNM1L
SUMO4
PCDHGB8P
AC021106.1
EAF1
TMEM184C
ITGB5-AS1
GIGYF2
AC011483.1
AC009495.1
AC113386.1
CCDC51
RNU6-1191P
HMGB3P14
ARHGEF2
IRF1
SPCS3
AL139246.3
MORF4L2
TRAPPC10
LURAP1L
AL606834.1
LINC02562
TONSL
MPHOSPH10
AC009803.2
TRPC4AP
NOTCH2
HIST1H3J
SRGAP2C
HFE

UGT1A9
DLG3
MUC20
ANKRD36B
SEC31A
TTC31
KYNU
SEMA3A
AL136295.5
PDZD11
AL591806.1
AC022126.1
CEP135
AC023906.5
NKIRAS2
AL121987.1
ORMDL2
AC124067.4
MRPL30
LEXM
ERRFI1
KRT17
PTAFR
RBM12B
NUP155
NRDC
DDX50P1
SMURF2
SCCPDH
PRKAB2
GTSE1
PYCR1
MLX
HOXA11-AS
IL23A
AC090791.1
AC026120.1
FOXJ3
GAPDHP71
SNRPEP2
VPS4B
PNKD
TIMM17A
EP300

AC114763.1
MTFR2
SFT2D2
C11orf86
CYCSP45
IFFO2
CTNNBL1
B2M
HIST1H2APS3
MITF
IBSP
WDR3
TATDN1
AL591806.3
PHACTR4
NIPAL3
AL512413.1
OSGIN1
LINC01719
MIR4767
DDX11
HMGB1
ASAP1
INSYN2B
METTL11B
HSPE1
LINC00705
DNAJB3
PPP1R3G
WASF2
AC069360.1
ARL14
PLXNA3
RNASEL
RNA5SP28
AC002066.1
ZSWIM6
NCR3LG1
KIF16B
BCL2L12
AC092718.5
HIST1H1C
PXYLP1
SNORD117

PPP2R2A
AURKB
PAPSS1
CR383656.1
ISY1-RAB43
AL133243.2
AC021218.1
PRAG1
RNF128
HIST3H3
HIST1H2AD
AC010271.1
HOXC-AS2
RHOT1P1
MBTPS1
SEC24B
MEGF6
MORC4
AC025252.2
DDB1
SINHCAFP1
DNA2
AC130689.1
PRR5L
ZNF114-AS1
AC011676.5
RCN2
DNAAF5
HIST1H2BM
ADAMTSL4
HIST2H2BE
PPME1
ACP1
AC004009.3
RPS6KC1
CMTM7
B3GALNT1
TCAIM
GLG1
AC021087.1
LINC02535
RPN1
AC009994.1
IHH

AC114812.1
PTPN1
ASB7
AC100854.1
ARNTL2-AS1
SHISA5
MED28P3
ADCY7
AC009093.1
AC008155.1
C5orf15
AC117409.1
MICU1
AAMP
SINHCAFP2
ZFC3H1
PAX9
HIST1H2BO
PTER
C5orf66
NCOA4P3
C15orf48
TM7SF3
CSTP2
IFNGR2
AC108449.1
RNF214
ZBED6
SETD3
SEL1L3
NUP35
NFATC2IP
CHD1
TRAFD1
SOCS4
NUP133
EFCAB1
SPTLC1P2
ERAP1
BCL2L2-PABPN1
AL021068.1
XXYLT1
AKAP4
AC022165.1

OTUD1
GGPS1
EFNA2
RN7SL809P
GPR1
TK2
HSD17B2
ADAM19
COL8A2
AL033381.1
AC100861.1
ARID4B
AL606534.1
TNRC18
ABCB11
RBM43
SHANK2
AADACP1
RAB31
VPS13B
LINC00920
C2CD3
SUSD6
CD47
TCERG1
STYXL1
NACC1
AC104024.2
TSPAN8
ATMIN
GATAD2A
PDE6D
SYPL1P1
PAFAH2
SPINT4
AC004009.1
RAB2A
NECTIN3
CEP170B
ANAPC10P1
EIF4G3
ZXDB
EHBP1L1
PHKB

LYPLA1P3
SRD5A3-AS1
TIMM23B
UBE4A
NSUN3
PTBP1
SLC44A2
RASSF3
RAB43
BATF
AC015971.1
HIST1H2BL
SF3B2
SNRNP200
AL354813.1
CCT2
RIOX2
MEF2A
FAS
ASCC1
BBX
FNIP1
FOXC1
BMP2
VSTM5
AC009226.1
SPATA5
STYX
MIR4435-2HG
NSMCE2
SLC29A2
AL445183.3
AC004009.2
KRT18P10
ERMP1
AC068946.1
RN7SL382P
LINP1
SRCAP
FPGT
FANCD2
ZNF480
LINC01615
PLAGL2

FAM160A2
AL136531.3
SUMF1
RF02141
EHD4
THUMPD3
ARF1
EHHADH
VDAC2
LINC02188
LSAMP-AS1
AC090695.1
PUS7
AP000688.2
ASCC3
AC026356.2
SNAI2
AQR
MBNL3
PPP1R18
PLK3
APAF1
PRKCZ
MKRN2OS
SLC22A13
AC090181.3
PACS1
SLC9A2
SLC50A1
DDX1
MAP4
SINHCAFP3
CAPN8
SYNE2
HIST1H3D
HNRNPUL1
GLYR1
LMTK2
MAFK
ITPRIP
AMOTL2
TP53I11
NTSR1
AC011773.3

ETNK1
GARS
TRIO
METTL8
THOC3
PODXL
RNF111
AL359745.1
HNMT
EPHA1
UGT8
LARP4
SCUBE3
MACF1
TPSP2
RAD23B
GCNT2
MYO18A
DDOST
UNC93B4
S100A4
PPM1G
NUP205
AL451164.3
PERM1
VGLL3
LHFPL2
NTF4
NOP58
HBP1
SLAMF6P1
AKR1C7P
C1orf105
AL133230.1
PSAPL1
TMX1
L3MBTL3
BTBD10P1
KRCC1
TMED5
PGAM1P8
AC092718.3
FOXD2
AL360007.1

ARAP3
GSTCD
ETV4
PSMD10
DDR1
AC013268.3
AC020900.1
TAOK1
AC022211.3
PLEKHG2
SHKBP1
ULBP2
DCP1A
TXN
AC073410.1
COL3A1
NCAPD2
AC009133.1
MON1B
IQCE
PDXDC1
C1orf74
SMCHD1
RNU4-53P
DVL3
PABPC3
ILK
MIR199A1
MRGBP
CYP51A1
SERPINH1
SCAF11
AL133370.2
NORAD
NSD3
RELL1
PBRM1
SNX27
ZNF720
SOS1
MGME1
EXOSC10
AC103923.1
SH3BP2

ZNF765
AC004917.1
RPS6KA3
SBNO2
IPMK
SBDS
CPD
FXR1
ABHD14A-ACY1
DNAH2
CUX1
AL133330.1
DRD1
KDM5A
OSBP
UBQLN1
PNRC2
NSUN2
ARHGEF5
AC004817.1
RPAP3
DCTN5
SLC17A9
LINC01376
AC005753.1
PARP4P1
RBBP4
SLC22A18
AL356273.1
STPG1
PCAT7
NPM1P5
SFTA2
UBXN10
NUP58
HOXA-AS2
PORCN
AC243967.2
UGT1A7
HERC3
IFIT3
INTS4
TRMT5
ECEL1P2

PSMD8
AC008147.2
KMT2E
CDR2L
HNRNPA1P3
DDIAS
BIRC5
KRT18P19
ATG14
TRNT1
PDZK1IP1
TM2D1
C10orf55
ECD
ARSJ
TMSB10
AC004596.1
MARVELD3
DPYD
KCTD9
EIF1AD
BUB3
AF124730.1
CCNE2
TRIM15
C4BPB
CFAP97
RPL37P1
AL365356.5
SLFN5
KCTD11
TCF19
AP001271.1
TDG
CLDN7
LFNG
HOXB-AS2
AC138305.1
LGALS4
MMP11
TSNAX-DISC1
ZNF532
SDCBP
NCSTN

KRT18P11
UGP2
AL356310.1
SOX21-AS1
ENAH
B4GALT4
HSPA14
FBXO38
ENTHD1
GRB7
ELF3-AS1
GDAP2
BAG3
ETFA
SNW1
NOMO2
LRRC6
STX3
OSBPL1A
TUBA1B
EPS8L3
SOX13
TNFSF15
GCSH
CCDC15
TSPAN3
DSCC1
MCM7
AGR2
SLC37A3
ADNP
MED1
CSGALNACT2
FER1L6
ACSL4
AC073569.2
CAMSAP2
FUT4
GALNT6
GSTP1
AC096720.1
B3GLCT
LINC01656
IPO7P2

YBX1P10
ESCO2
HSPD1P4
AC010328.1
BRIP1
MREG
AC021078.1
MBNL2
CCDC9B
KIF13B
AC010761.4
RASA3
SETD5
AP002373.1
AL353807.3
AC123905.1
MCL1
SPIRE1
AC083899.1
EPSTI1
PLEKHM1
IRX5
TRIM33
ZDHHC20-IT1
MBTPS2
AL445435.1
PI3
SEMA4G
AC108860.1
CEMIP2
TGFB2-AS1
AL590004.3
CTDSPL
HIST1H1T
TRMT11
AL035458.1
FIGNL1
DHRS9
DKC1
GALM
AL357033.2
MTX2
AL136115.2
MGAT3-AS1

COL1A2
LBX2-AS1
HOXA9
ATRX
AC011753.4
NRAV
TK1
AC078899.1
VGLL1
CMTM3
ZC3H11A
CNOT9
DEFB131B
LRP1
KDELR3
ETHE1
PCDHGC5
PLOD1
DNAJB11
AL109614.1
BRCC3P1
NIPA1
PUM2
MTBP
PSMB9
AP001033.2
LINC01269
CTPS2
PHF14
AC008687.2
CXCL8
LINC01633
HSPD1P1
ARL4AP4
AC106886.2
MIR3153
SET
RFC3
LINC01748
GCOM1
RAB9A
PSCA
C5orf24
WNT7A

AC040174.1
TNFSF4
LPGAT1
AC114812.2
MROH9
LAPTM4A
PPP1R2
MIR4263
FNBP1L
IFNAR2
AC104109.4
HCCS
UGT1A1
AC026401.3
PLPP2
ABHD2
AC127496.2
LINC02519
LINC00605
SNORD89
NUBPL
FOXC2
TKT
AL158201.1
SLC15A4
EI24
SCN5A
HS3ST1
DIRC2
NECTIN2
SRGAP2
PSMC2
RNF19A
CSTB
CYP2C18
PPP2CA
TALDO1
FRYL
RAB11A
AC091173.1
IFIT2
C1D
HPGD
EMC1

SMOX
TUBBP1
PREP
TRNP1
PKMYT1
RN7SL36P
LPCAT4
AL357558.1
AC098934.3
KCNA10
GPR157
DERA
CTSV
TMEM183A
AC015983.1
RBM27
AP005233.2
GPATCH8
GOLIM4
PIM1
LTBP1
MYCBP
SMC2
ALAS1
TFAM
REPS2
GOLPH3L
ARID4A
MED21
IL18
CHP1
DHDDS
PDS5A
KRT15
STK38
PGAM1P7
DHX8
SNORD12B
FAM83A
TNNC2
AC026462.1
GCC2-AS1
PAPPA
HIVEP1

FAF2
PTGFRN
ZFYVE21
PHF13
ARPC1B
TOMM20
CRABP2
ATF2
LOX
S100A6
ANKRD42
MAP2K1
STAG1
SLC34A1
SERTAD3
CUL4B
EHD2
UTP14A
PRAL
SLC44A4
CPSF6
ZMYM2
KDM3B
SLC38A7
HSPG2
MYH16
CCNI2
AC130456.2
PIK3C2B
AC008687.3
RNU6-863P
BTF3L4P2
RFC4
HEATR5A
VKORC1L1
HIF1A
GALNS
RBMS1P1
AC011611.6
MKLN1
CSNK2A1
AF127577.4
CPEB2
DUTP1

TRAF4
HSPH1
AC019117.1
ANKRD28
AC010735.2
DCTPP1
RGS6
CNOT2
TMEM267
TNKS2
GCN1
MRPL49
XPO5
TYMS
ZNF384
C5orf51
USP53
TROAP
AC025575.1
CD58
COL8A1
GRTP1
SYTL5
EFHD2
ARL4AP5
UBE2W
ARHGAP18
EIF3I
YTHDF3
ELF3
BMS1
AKT1
AC114812.4
CUL4A
AP000619.3
CATSPER1
TMEM231P1
RRAGC
SEPT7
SMS
CDC27
MAGI3
YKT6
DST

RAB5B
DCAF1
SUPT16H
RNF213
BCL2L14
CD164
DNTTIP2
LINC01473
CHST6
LINC00939
AP000695.3
SLC45A3
PPP1R12A
LY6G6C
OSBPL8
OCIAD2
UBE2L6
SLC25A40
CAPZA2
UGT1A13P
HDAC9
CANX
MKRN2
CCDC43
GK5
LINC02323
RBM47
MTOR
MTCH2
DDX10
COP1
RAB8A
KRTAP4-1
TUBA4A
PPRC1
AC011242.1
CYP2B6
HUWE1
PLPP4
FOXD1
CDV3
AC022973.4
AC093520.1
HIGD1A

TMA16
ETV7
NPAT
MCM6
PNPLA3
AC110760.3
DMBX1
HDLBP
ATF6B
THRAP3
LINC01614
RNU6-767P
RP1L1
MIR561
RHOQP1
CTDSP2
ANKRD11
AC004980.2
AC022784.5
MLPH
NUTF2
CARS-AS1
MAP3K5
ZNF12
UBE3C
AC023271.1
PICSAR
SPTAN1
AREG
IMMT
AC011476.3
PRSS12
PRDX1
CCZ1B
IL20RB
AC025423.1
RHBDF2
CASP6
MRE11
COPS2
CYP4F12
WDR55
CLIC3
PRSS51

NMU
NEK6
ALPP
DPAGT1
PLEKHG5
KRT6A
AC091931.1
GPS2P2
AC090833.1
LINC01137
NRP2
RAI14
MTIF2
COPS5
HCG15
KRT13
TMEM135
AL662791.1
CIAPIN1
ZNF702P
EIF4A1P12
PCDHB9
FBXO36
DNAJC11
DBF4
CENPL
FRK
RNU6-652P
GJB6
AC008687.4
ETF1
SUZ12
ABRACL
DHRS3
UBXN7
MRPL15
AC131888.1
MTHFD1L
PCDHB14
AC138392.1
ARHGEF34P
SHH
GPR35
KPNA3

CASC8
NTM
EDAR
CASP7
AC096536.1
LINC02057
TLCD2
TWF1P1
EPS8L2
MYO5B
AL139039.1
PCSK5
DUSP5P1
CAMKK2
ALDH3B2
NUP62CL
SPINT1
RICTOR
SRGAP1
AC011294.1
FAM222B
MB
ZNRF1
ATP7A
NEK4
RNU7-159P
GSDMB
TJP3
SLC10A3
KLHL20
FKBP14
AC105446.1
ACOT4
PLEKHA3
USP37
RAET1E
AC073283.1
AC025580.2
ABHD11-AS1
NSD2
CENPU
CNOT8
OPLAH
TGOLN2

THRB
SULF1
SALL4
TGFB2
HIST2H4A
AC097637.1
HOXB5
FAM83E
DENR
ARHGEF19
RYBP
INO80D
NAE1
HPDL
FAM129B
PTMAP4
DNAJA3
EXOC8
YIPF4
TMEM79
AC068594.1
METTL5
SMARCE1P1
SLC10A7
PON2
RN7SL706P
LINC01213
FER1L4
MISP
TCF12
ADGRG1
SH3D19
GANAB
AC004808.2
RAN
GTF2B
PPP1R14D
C11orf58
AC090505.2
MORC2
UPK2
PADI1
NCOA2
NEMP1

TRIB2
MYADM
WNT7B
KIAA1211
PTPRE
SNRPGP15
OSBPL2
TSN
C12orf49
AC244153.1
PROSER1
CDK18
MARCKSL1
NCOA3
GTPBP10
RNF44
AC100821.1
POLB
AC239798.4
CAD
LNX2
XPNPEP1
AC243960.2
AC007336.1
LRRCC1
APOL6
AC005550.2
RNF7
PRDM8
AC068580.1
ATP10B
ALPK1
AP000695.2
AC007285.2
LTBR
FBLIM1
AL590652.1
DUSP5
ZBED2
LINC01705
MIR6835
BTBD7
CGB7
LINC00630

PLEKHA5
ALDH18A1
TGM2
PSMB8
GPX8
RNU6-403P
AC139722.1
ZNF274
JOSD1
HSP90AB1
SLC22A18AS
ACBD3-AS1
USP24
AC005476.2
STK24P1
RF02140
TOB2
RB1CC1
MIGA1
RF00405
KLK10
CD44-AS1
AC130456.4
HOXA11
VCAN
FAM214B
KLHL30
RPS12P26
EML2
WASHC5
DCLRE1B
KLK7
AC140479.2
HNRNPA2B1
TMEM173
LRRC31
ZFAND1
ATP1B1
IDE
ATF1
ABCA13
CASC4
BX640514.2
GINS4

ACOT9
GAPDH
AC108058.1
S100A2
OSTCP1
SERTAD4
KIF26B
RSAD2
SLC25A13
CBL
CHMP4B
PCDHGB2
CDC42P3
DSG3
KIF2A
COL22A1
FAM49B
RUNX1
UBXN2B
ADAT1
CAPN5
LINC01776
PICART1
TMBIM6
COL5A1
FAM118B
LINC01842
LINC01873
EIF4H
CDH11
LPCAT3
UNC5B
TAGLN2P1
MEMO1P1
DNM2
RDH10
ZBTB1
BLOC1S2P1
TMED7-TICAM2
ESF1
AC115284.1
SLC35E1
DHX36
ATF7

FLRT3
G3BP2
CDC42BPB
ZNF587
BRCA2
NUCKS1
BCAS1
MOSMO
SH3BGRL3
UBE2A
WNT2
AC023024.1
FRMD4B
ZBTB11
AC027290.2
ZC3HAV1L
HOXB8
ZC3H12A
AP000904.1
CAND1
HERPUD2
IER5
WNT10A
PDK1
SPOCK1
LATS2
SPAG5
AC023024.2
BAG4
FOXA1
SRP9
AC073333.1
RAB25
CDKL5
TIPRL
KCTD1
ATXN2L
AC105935.1
PLAC8
HOXA1
KLHL24
PARG
FGFRL1
DCAF17

AC055811.1
C16orf70
HMGB1P6
CTHRC1
PAIP1
ZSCAN4
UBQLN4
MFSD6
AL353693.1
EDEM3
FAM208B
TRAPPC6B
TAF5L
TGS1
SNX33
MOCOS
AC107021.2
SCD
SPINT2
TNFRSF11B
TNFSF9
SLC39A11
MPZL2
TOMM34
AC008406.3
UBE2D3
CHRAC1
FAM210A
CEP170
HIST2H2BF
NDUFS1
ADAMTS6
GNL3L
SLC12A6
COLGALT1
CCDC34
AC073551.1
HSPD1P5
PSMD11
AK2
SPRY4
COL5A2
RAB5IF
HPCAL1

ORC4
GON4L
AL451042.1
HMGN4
TICRR
DYNC1I2P1
UBA1
CYP2T3P
EPCAM
RER1
PPIAP22
IL7
AC004990.1
SYNPR-AS1
TAF12
AC007327.2
CMPK1
HSD17B12
HMGCS1
ZCCHC14
AC091057.1
IVNS1ABP
NAV2
RBM12
AC012613.2
LRRC37A15P
NKILA
MPRIP-AS1
RN7SKP296
SIAH1
ZNF148
EFNA4
ZBTB45P1
RFLNA
BBOX1-AS1
NBN
TAB2
ARPC5
TRIM6
B4GALT1
NF1
ABO
SLC35C1
EMBP1

IPO9
TMEM189
ACY1
DHX38
IST1
HOXB6
SLC38A2
LINC02584
COPG1
TSPAN15
PTK2
GCNT1P3
C11orf80
FUT2
TEAD1
HIPK1
TMEM40
AC073052.1
SIKE1
IGFBP3
FZD7
DGKA
ANXA8L1
RBP3
KPNB1
ANTXR1
PROSER3
MIR135B
ABHD4
LSG1
AL365181.3
PTTG1
CARD11
SLC9A1
IGF2BP1
AFF4
RASA2
AC083841.2
AXL
TLK1
NPFFR1
AC016722.2
NECTIN1
AGRN

CABLES1
TMEM139
LRRN1
MYO19
LMO4
TRIP13
BHLHE41
CAPRIN2
LINC00887
ARHGEF18
IRAK1
EIF2B2
TMLHE
DENND1B
AC026470.2
AL157786.1
AC142381.3
NECAP2
PRXL2B
ATXN1L
UBA2
TFDP1
FIP1L1
PSMC4
CST6
TMEM127
AL603832.3
TWSG1
FADD
KCNK6
SULT2B1
HES2
IMPDH1P5
AC107308.1
PLOD3
AC114812.3
EFCAB14-AS1
CNKSR1
WFDC3
STIP1
MIR7973-1
TLN2
RAC1P2
GBP1

EIPR1
SLC16A4
LIN7C
FAM220A
PRDM10
KMT5A
FRMD8
TBC1D2
FOSL2
UBFD1
OTX1
ZNF367
MIS18BP1
C8orf74
AC093110.1
ARHGEF12
GOLT1B
NKAIN3
HSP90AA1
FAM84B
TTC9
RMI2
TBCCD1
DHX32
HAVCR1
PCDHGB9P
RAB21
EARS2
ERGIC1
MANBA
MIR4284
CPSF3
LBR
IMPAD1
ALG11
LINC02595
HES1
PMEPA1
RTP4
BCO1
DDI2
LPAR5
H3F3A
DPP9

UGT1A6
AC090673.2
CRYBG1
PCLAF
CSE1L
PNO1
AC092611.2
AL355312.3
RUVBL1
DDX19A
PRPS2
USP1
SEPT8
AKR7A2P1
TM4SF1-AS1
EMC2
SPEN
MYO5C
NR1I2
AL138689.1
AC005261.5
CDC42EP2
KRT19P1
CTDSPL2
INTS8
CDKN3
ZNF654
MASTL
AC007283.2
OPTN
ZNF805
MAIP1
UNG
SLC20A2
AARS
CCSER1
DNAJC10
TSPAN14
AC025575.2
BST2
SOX9
ITGA11
S100A14
RIPK3

DEPDC1B
AC106712.1
RTKN2
GPC4
STAG2
LAMC1
SLC39A4
SULT1B1
RABGAP1L
MX2
EGLN3
RNU6-1161P
AP005230.1
AC093724.1
MECOM
LRRC40
FAM72A
HRASLS2
RPS12P2
WFDC13
ALS2CL
MSX2
RNA5SP323
PKD1L2
GNL3LP1
FAM83D
ADD3
AC009237.15
PARP3
NXT2
BZW1P2
FXYD3
SRSF4
MPZL1
HSPE1P5
SAMD12
AC023632.6
PDGFC
RPRD1B
C16orf46
ANXA8
AC245041.2
ZNF701
TNFSF11

LRRC57
RIT1
LMNA
EMC8
AC092881.1
RCE1
SOWAHB
AC004920.1
LOXL2
CES2
TSPAN6
MCM8
SNRNP27
CTCF
DUSP7
SH3KBP1
GOLGA4
NOL10
TMEM44
LINC01655
LINC02081
ZNF644
MIR4795
SIX1
AP000759.1
TRIM31-AS1
HMGB1P5
RNU6-90P
EIF2S2P4
CELF1
POLD3
AC012531.1
LSM14A
ACTN4P1
FMR1
CMPK2
GALC
CCDC68
GRHL3
USP48
AC027544.2
AL591135.1
ARFGEF2
FBXO11

PTPRK
SATB2-AS1
KLHL28
STAT6
ZNF623
MCFD2
AMBRA1
PML
ADAMTSL5
POC1A
CDCA2
RRN3
LYPD5
SLC6A20
SSH3
PKMP1
PHF20L1
XPOT
AL450992.1
THOC2
SH3D21
LDHAP7
PTGES3P3
DHCR24
NUP54
ZBTB41
C3orf38
RARRES3
XAF1
KIAA0556
NEDD4
FAM86LP
GPR87
RPL13P5
GALNT2
CNBP
CD44
ZBTB7B
TPD52L2
AL391280.1
AC093616.1
DEK
POLE2
CCNF

CLDN23
RPL23AP77
ERI1
PSD3
FGD4
WASL
PSME3
BCL2L15
TPR
ALDH3B1
AC018629.1
BNIP2
LINC01133
JPT1
MIR3131
CHD7
HOXA10-AS
SPDEF
AC124947.1
RHOA
MYNN
NAA25
AC004837.2
PPP1R11
NIFK
RNF225
AL031009.1
SELENOF
SIAH2
GTF2E1
VEGFA
LINC01819
SLC35D1
AL139039.3
ETV6
CKLF
PRELID2
STT3A
CHMP1A
LYPLA2
JMJD1C
RAB38
KDELC2
FUT3

RARS
SELENOI
CCDC120
C2orf49
KRT8
TTLL4
ACBD7
AC022149.1
CYP2S1
SEMA7A
CUL3
B3GNT7
VCAN-AS1
COL12A1
AC078883.1
ANXA2P3
STAMBPL1
ACBD5
WNT5A-AS1
SH2D4A
HAUS2
STK31
WNT5A
ZMYM5
YTHDF2
SLC16A3
CRNDE
ABCA12
CORO1C
SLC7A7
SUCLG2
CKAP2
AP003555.1
IMPA1
AL445933.1
SLC12A2
ANKRD20A5P
UGGT1
RAB7A
ZNF597
PLEKHA1
TRIB1
LIMK1
TFAP2C

EFR3A
GRAMD2A
MED17
SRI
TMPO
GBP4
ZNF600
AC011742.3
LYPD3
ZNF440
FAM72B
PHC3
MFN1
PHLDA2
F3
CXorf38
FAR2
SH3BP4
VAPA
PIMREG
VPS37B
TRAK1
TAF4
PTMAP5
AC009237.14
HLA-V
KLK6
VTI1A
OTUD4
TMEM30B
NMUR2
ARMC1
TCF20
RNF40
TRPM4
PPP1CB
FANCB
SLC17A5
ANKRD27
AL451042.2
TEP1
GMFB
GRAMD2B
UBE2H

	AC135050.2
	CABP4
	CD151
	MCMBP
	TNFRSF10B
	EFNA5
	RALGAPB
	XRN2
	EPS8L1
	UBE2T
	DOCK5
	CTBP2
	MYBL2
	GLRX3
	BIRC6
	CASP4
	SEC24C
	STEAP3
	PARP9
	RAD21
	LDHAP4
	SEH1L
	AL513314.1
	TNPO3
	OR7E110P
	AHCTF1
	EFCAB14
	EPHX4
	AC108681.1
	ELK3
	ARHGAP23
	VPS37C
	ACTB
	NGEF
	SLC7A11
	PIK3C2A
	CPSF2
	FTSJ1
	SEPT11
	PTPRH
	GNB1
	CYFIP1
	USP6NL
	KPNA1

RAB3GAP2
UBA3
SLC35B2
TBC1D23
RALGPS2
TAOK3
GTPBP2
CDC42BPA
HSD17B11
GNL3
MAPK3
AC132186.1
ZNF770
PGAM1
FANCI
UBE2I
KLK8
PLSCR1
HAT1
GTF2I
ZNF260
ALDOA
AL445305.1
ZFP64
ATP1B3
SAPCD2
RECQL
SMILR
GCNT3
RF01210
GYS1
SIM2
KRT8P32
NME7
AC113404.3
CREB1
SQSTM1
RPS6KA1
DDX27
PPP4R2
LAD1
SQLE
FEZ2
ANKRD22

SNAPC1
GOLGB1
RREB1
RNMT
CARM1
EPS15P1
PANK3
MNAT1
NR1D1
ERI2
SEC23IP
C1orf198
RAD54B
CLSTN1
AL359851.1
PTPN9
HNRNPK
NCBP2
CNN2
CARMIL1
VILL
PPP1R15A
ABCC3
ZNF860
ARIH1
EIF3A
TFRC
ARL4C
AC244517.7
BDNF
COL11A1
AC009065.5
THBS2
GTF2H1
CD55
ZFPM2-AS1
CIT
AL136531.2
CEP85
AL049872.1
AL161431.1
SLC22A3
ADAMTS12
RBMXL1

UBAP2L
LPP
AGMO
RTCA
PRR13
RPGRIP1L
PSMB2
TAGLN2
LRIF1
UBE2Z
KNSTRN
WAPL
ITPKC
RHOD
CDK7
BTNL8
STT3B
EVPL
TIPARP
TRAF6
COL7A1
YWHABP2
SPRY4-AS1
EIF3M
SASS6
MVP
CHMP1B
ADAR
SSRP1
PLEKHB2
LINC02038
XRCC4
CTSE
SLCO4A1
HOXB3
PRKG1-AS1
RRP15
DOCK9
EIF6
PRKRA
TTC22
DPH6
PITX1
NYNRIN

ENDOD1
UGDH
DLEU7
HNF4G
ZNF114
RAB11FIP5
PTTG1IP
LACTB
PFKP
TBX6
SLC16A5
SUMO3
HECTD1
ANKMY2
RBMS1
AC010735.1
CLIP1
TOR4A
RAPH1
TARS
ALG10
SLC38A1
ADGRF1
CYB5R2
ANP32E
CKS2
TMED2
STRN3
TNFRSF10A-AS1
ASXL2
COG5
ORC6
ST3GAL1
ENTPD7
LINC01572
WDR12
AC254633.1
VIRMA
CRIM1
HNRNPL
TUBB
RNVU1-1
ADGRE5
MUC16

KRT18
SRRM1
SUDS3
HIST1H2AG
PIP5K1A
PDIA4
AC068580.3
ZNF841
EHD1
ACTBL2
EDEM1
LINC02163
AC009237.8
TBX15
ZNF207
IL17RE
SF3B6
DIP2B
DLST
AP1G1
GPN1
AL360270.1
TRIM16
C16orf72
TM4SF1
ZC3H18
AL162632.3
NFXL1
PRSS21
ZFP36L1
PLAU
NEMF
SLC6A6
EXT2
ACAP2
TTC9C
AF201337.1
SIPA1L1
FAM199X
RN7SKP16
NCAPG2
CASP10
CACUL1
IDH1

PPL
SYTL4
ABCE1
AP002761.4
PSMA5
OTUB2
FAM126B
TMF1
RAD51B
PIK3CA
CLINT1
CDK12
C8orf31
LRRC8B
MAPK13
CLDN16
MXRA5Y
SDC1
CBLC
LRRC42
DNASE1L1
USB1
ORC2
ZC2HC1A
PPP1R1C
HIST1H1PS1
NHEJ1
CCDC88C
TRIM31
VCPIP1
OVOL1-AS1
HS2ST1
SULF2
PSMA3
SCEL
CFL1
OAS2
LRCH1
PTMAP2
EIF5B
RHOC
PSMD2
BORA
DYNC1H1

HOTTIP
NECTIN4
OSER1
EIF4G2
AL928654.4
CEACAM6
MAL2-AS1
LMLN
SDCBP2
SHCBP1
AC114488.1
ZPR1
SRSF10
AC008555.1
METTL9
LINC02257
LRRC58
ZBTB7A
ABHD3
ARFIP1
SRGAP2B
AL121772.3
AIMP2
UBXN4
WTAPP1
ATR
UACA
MED14
CNIH1
HIST1H2BJ
TRIM21
REXO2
SMAD6
UBR5
PPTC7
GTPBP4
ANKRD36C
MIER1
UNC13D
HNRNPC
ELF1
TMC5
PNP
AL451139.1

ZDHHC13
PLEKHG6
RAB27B
NUS1P1
DPY30
AC112220.2
RAB3GAP1
CWC22
SGPP2
STAM2
TACSTD2
PARPBP
ZNF281
DCAF13
LEPROT
AL354714.2
YIPF5
SERINC5
OSMR
NFYA
MOSPD1
FAM135A
NBEAL1
EIF1P4
AC006971.1
PTPRU
KRT8P46
CNIH4
SRP54
PRSS8
NPEPPS
DCBLD1
AP003555.2
PIGA
AC002128.2
SUMO1P3
BLACAT1
POU2F3
ACOT11
C6orf223
KRT7-AS
EPRS
RETSAT
TMEM106B

SMIM15
MAD2L1
RSPH3
AC078820.1
AC073046.1
RAD51
ZNF468
PPIG
AFAP1L2
ARHGAP27
ERBB3
ST14
KPNA6
TOX4
FN1
MTPN
TNKS1BP1
PLXNA1
TPRG1LP1
TRIM34
PPP4R1L
GRIN2D
PIK3R4
VTA1
POC1B
MAP4K5
RNF169
HOXA3
PARN
NEMP2
SAP130
AC090578.2
SUMO1
AL121578.3
KIAA1841
AC243967.1
KIAA1551
TUBB3
EIF2S3
TM9SF3
TSKU
FZD5
VAMP3
VSIG10L

SMAGP
KLF4
AC092142.1
NLN
MELTF
LINC01232
KIF18B
DOCK7
STEAP2
SEPT2
AC009065.2
INHBA
DYRK2
SORD
HNRNPA3
H1F0
FEM1C
NCAPD3
ROCK1
CBWD2
EIF2A
SRD5A3
GTF2IRD1
TMEM170A
ZMPSTE24
MAT2B
CEBPG
DDX60L
MDFI
GOLGA5
PCYT1A
NOB1
AC022509.3
ATP2A2
TYW5
AP003031.1
COX6B2
IL13RA1
C5orf22
TRIM59
MYOM3
ASF1B
AC024941.2
ANAPC1

FAM120A
ELMSAN1
AL157838.1
ATAD2
LRRC59
PRRG4
LRPPRC
SPRTN
SGPL1
FAM114A1
LGR4
CPOX
LRP5
LINC01876
RNF223
DUSP10
LMNB2
RN7SL368P
CD9
CDKN2B
QSOX1
LINC00973
EXPH5
LDHAP3
AC008083.1
EXT1
SAE1
STAM
MYH9
LDHAP5
RSF1
NOLC1
GLUD1
SIX4
FASTKD2
MTMR11
RAG1
TTC7A
ALS2
CAP1P2
STS
GGCX
SKA3
TBC1D8B

CERS2
DAPP1
PKM
USP4
ZMYM1
USP39
MYEOV
DIS3
AFAP1-AS1
MZT1
CHEK1
HIST1H4I
PDZD8
PRKDC
NAT10
ATP2C2
ESCO1
DPM1
SLC25A5P5
PLK4
SHROOM3
LINC00857
SAMD9L
CDC42SE1
TMED7
MCM4
SH3RF2
AUNIP
BAZ2A
MAP3K2
RPS6KA4
CCDC6
LRRFIP1P1
NPR3
ARL8B
FKBP9
VDR
PLAT
EIF2AK1
DSC2
AC120498.4
PRRC1
RBMS2
ACSL3

ACVR1
TPBG
BEAN1
MCM2
AC020891.2
AC245041.1
CDS1
AC078883.3
GNL2
DGKH
SCNN1A
EIF3C
RNU6-26P
TMEM144
SNTB2
NUP160
UBE2E1
CSNK2A2
FOSL1
FAM177A1
KIF5B
ATIC
FUBP1
QSER1
SH2D3A
ABLIM3
LEMD1
TBL1XR1-AS1
ARFGEF1
ENTPD5
TAF2
RUNX2
NAALADL2
ZDHHC20P4
NFE2L2
TMEM165
C15orf41
FRMD6
YARS2
LIMA1
MACC1-AS1
IKZF2
KDM1A
XRN1

AP3S1
WDR75
PABPC1
UBR4
GTF2A1
CDC6
BMP4
PROM2
AC093162.2
PPIAP45
NCOA6
ERGIC2
SNX7
NOL4L
SFR1
CAV2
SENP2
MCM10
GSK3B
AC007255.1
LRP10
TXNDC9
OSGIN2
PLEKHA8
CD3EAP
ADAM8
CMTR2
AC007537.1
CEACAM1
AC002524.1
CSNK1G3
INF2
WDR1
AC036176.3
TOB1
PTP4A2
PCBP1
FCHO2
GNGT1
BPNT1
AGO2
WIPF2
ENC1
TM9SF4

CHMP3
NQO1
CCNG2
AL023775.1
COL10A1
SESTD1
STPG4
FOXN2
BIRC2
CAPG
CALM2
ZBTB45P2
RAB11FIP1
AP000439.2
CASC19
SAMD4B
PTGES3
UBE2D1
TFPI
RF01233
DOK4
GTF3C2
MANCR
MMP14
DNMBP-AS1
TAP2
ATP11A
MYL12B
PPAT
ADM
MTHFD2
EXO1
PKP4
CCND1
SMG7
UBN1
SHC1
HNRNPCP2
LINC01559
MXRA5
ERCC6L
GTF3C3
AP000695.1
YEATS2

AP2B1
GNPNAT1
ATP2B4
CNOT6
C1orf116
VSIG10
LONRF3
FUT8
TOPBP1
OIP5
ERV3-1
PDP1
EFNB1
SAV1
ISG20L2
AC005034.3
CALU
ARMC10
SEC23A
AC020891.3
TMEM50A
METTL15P1
RLIM
SERBP1
KTN1
BRWD3
CNOT11
IL1RN
CD82
SBF2
MFSD14B
PARP14
PTPN14
TUT7
KIAA1217
CHMP4C
PPP2R5E
GALE
ZNF816
AC068580.2
FBXO45
STAT1
RNU6-1266P
ALG1L

POLQ
MCU
NUFIP2
APH1A
ZSCAN20
XIAP
FLNB-AS1
AC141586.1
GALNT7
NUP107
RRM1
RNF19B
RN7SKP97
CLIC1
CA13
PTPRR
TMEM33
NDC80
AC010148.1
MIB1
ZWILCH
AC046143.1
KIFC1
AP000619.1
RBMS2P1
COMMD2
TMPPE
MSLN
TMEM39A
DAG1
TXNRD1
PMAIP1
HAS3
AL606490.8
F2RL1
RELA
NEIL3
S100A11
HMGXB4
MAGT1
SF3B3
ZNF845
AC005993.1
PSMA1

RAD54L
TMEM63B
SERINC2
HNRNPU
SEMA4B
CORO2A
LSR
NRBF2
FCF1
AC026356.1
LUZP1
HELZ2
SRMS
CYP27C1
NPAS2
DCBLD2
FAM160A1
RSRC1
PTGES3P1
COPA
GRHL1
CENPA
SCYL2
VGLL4
OR7E14P
YME1L1
TNNT1
GFPT1
VANGL1
DHX9
PPP1R15B
SLC52A3
NCAPH
CLSPN
SF3B1
ESRP2
MYO6
GNG12
PAK1
DNAJA2
NUP37
TCEA1
SATB2
PLOD2

LRRC8A
DTX4
SNX12
RARG
EHBP1
METTL15
7-Mar
YOD1
PTGS2
KDELR2
RTN4
GJB2
ANO6
PSMD10P2
HSPD1
PSME4
NUP93
CD46
DNMBP
CDC42
TP73
MRPL3
NIF3L1
VDAC1P2
GBP2
TMEM45B
SP140L
ANXA1
FOXM1
EIF2S2
HTR1D
EHF
CEBPZ
ERBB2
RPSAP52
C5orf30
AMMECR1L
SH3PXD2A-AS1
MIPOL1
SNAP23
IGSF3
ARPC1A
SEC24A
FA2H

LY6E
NUF2
PAICS
GPBP1L1
ZNF460
MOSPD2
PSMC1
OTUD7B
RRAS2
ICK
BICD1
SRC
S100P
KARS
MAP3K13
LONP2
CDR2
HIST1H2AC
NCAPG
CDC25C
ACTN1
CDCA4
PNPLA1
AP1AR
DYNC1LI2
PPP1R13L
XPR1
JUP
ESPL1
ADIPOR1
TRIP10
MARK2
QTRT2
POGK
FAM111B
SLC30A7
MUC1
PLIN3
GDE1
TWISTNB
UBASH3B
KRT8P35
IGSF9
ZNHIT6

DPY19L4
LINC00346
SLC1A5
DIAPH2-AS1
STX6
KRT19
TRAM1
G3BP1
COPB2
AC022150.4
UCHL5
RFWD3
5-Mar
CREG2
ARHGAP12
RBBP8
TMEM92
PIEZO1
SLC7A6OS
CCNB2
PPFIA1
CEACAM5
PAFAH1B2
ENO1
ATL3
SDE2
ITGB5
GFM1
LINC02086
TMEM159
ANXA2P1
SGO1
CDCA5
CCT6A
CENPK
MPZL3
RSPRY1
IWS1
RAB43P1
LIMS1
HKDC1
DLG1
SMG1
RNF141

CCT5
HHIP
DESI2
TPM4
HOXB7
PLS3
ARPC2
SYTL2
APOL1
SLC39A1
CAMK2N1
AC004943.2
AP003555.3
CERS6
PLBD1
HDAC1
HOXA6
ERLIN1
MRPL19
PTGES
S100A16
PRR11
FRRS1
USP54
ZWINT
DPP3
AC021876.1
UCA1
ARF4
RASA1
NAPG
IFI27
CCNB1
SEMA3C
GBP6
ERBIN
MSL3P1
RAP2B
RASEF
PKP3
INTS13
MRPS35
RIPK2
OCLN

CKS1B
KRT7
XDH
CBFB
OASL
MTDH
USP14
LY6E-DT
PCSK6
DYNLT3
RNF39
SH3TC2
RLF
SRFBP1
KDM2A
ATP13A3
KLF7
SERINC3
KHNYN
TMCC1
GINS1
EXOC1
TNFRSF12A
ANXA2
TAP1
OLA1
RCC2
AL590723.1
TRIM29
UBE2K
PRR13P5
PRSS22
CPNE8
GNAI3
MPHOSPH6
GALK2
TANC1
GREB1L
BROX
F11R
LDLR
BMPR2
EMB
UBE2C

STEAP1
FAM83H
DNTTIP1
RRAS
DARS
CDYL
SLC20A1
SPAG1
FAR1
TSPAN5
TMEM182
KHDRBS1
YARS
PNPT1
LNPK
PRKAA1
WWP1
KIF15
TWF1
TCEA1P2
AC112777.1
UBE2V1
TRAF3IP1
AL133370.1
KRT80
P4HA1
UBE2E3
HK1
WEE1
BOLA2-SMG1P6
GART
HNRNPR
OGFOD1
KNOP1
TROVE2
USP10
MBOAT1
RN7SKP116
TRIM5
EML4
RND3
ELOVL6
APLP2
OTUD6B

NAA15
AC013410.2
IFIH1
TMEM43
MICAL2
MAPK14
PKP2
MTX1P1
XYLB
RP2
HMGA1P1
TUFT1
DPY19L1
EIF2S1
RNF2
DR1
MAML2
SPTBN2
AL606489.1
LPCAT2
SMARCC1
OPA1
GPR39
SLC44A1
DARS2
RCC1
SLMAP
PICALM
SFXN3
SH3GLB1
ABHD17C
NT5E
GSPT1
GULP1
NEDD1
RAD51AP1
SPIN4
AL590666.2
GBP3
GMPS
SERTAD2
NFAT5
RAD18
ROCK2

CNOT1
MRPS10
MUC17
MFSD9
TTK
AL049555.1
LINC02014
KDM3A
RBM41
FSCN1
SLAIN2
ITGB1
RHBDF1
GALNT10
ZNF488
CLDN4
OXR1
ATP5MC1P6
NUDCD1
KIF2C
CDC20
PSMC6
ITGB8
TINAGL1
ABCC1
MROH6
UHMK1
DIAPH3
E2F8
SLC13A5
WDR44
E2F3
WDR26
C1GALT1
LGALS3
FANCA
LRRFIP1
KBTBD2
B3GNT3
SGO2
TTYH3
CSNK1A1
BHLHE40
FERMT1

PLEKHN1
REST
CCNA2
PLCB3
RAP2C
SLC39A10
NUP98
EPHA4
PSMD14
TNFRSF10A
XRCC5
PMM2
GNA15
OLA1P1
ESYT2
YWHAB
KPNA7
VRK2
C6orf132
C3orf52
UXS1
HIST1H3H
PAQR4
SP100
MIR196A1
S100A10
ABI1
LINC02041
KLF6
KNTC1
C6orf106
MKI67
AC003965.2
MIR4653
CLDN12
DLG5
PRR15
AC007785.1
CGAS
PRMT3
FAM91A1
C19orf33
SH3RF1
HOXB9

PPP1R3B
NIPA2
ANKRD50
PATJ
ARAP2
ESPN
SYPL1
ACSL5
TANK
ATAD1
DTX3L
NUMB
U2SURP
DLAT
SOX4
LSM12
NCEH1
HNRNPLL
MPP5
AZIN1
PLCD3
NDC1
JPT2
ELOVL1
TFAP2A
N4BP1
UTP4
NUSAP1
CLDN1
FARP2
PRC1
FHL2
CKLF-CMTM1
TMEM41B
LINC02577
TNIK
PLK1
PDHX
DUSP6
LANCL3
HOXB-AS4
IQGAP3
RAP1B
MGLL

COQ10B
IRF6
PYGB
CDH1
TMTC2
TJP1
VDAC1
ITGB4
OAS1
MYO1E
ARL6IP1
CAPZA1
RRM2
XKR9
EXOC6B
ZDHHC9
SMAD3
PUM1
TMEM105
PELI1
HPS3
NAA50
IQANK1
WDHD1
HIST1H2BC
DDX60
LSM12P1
LRRC8E
PTPRF
RYK
RNA5SP18
CMTM1
MTHFD1
NPC1
STK39
HIST1H2BK
DTL
GTF2H3
AC026877.1
ZFP91
SPRED1
HTATIP2
VASP
TFG

AC007663.2
CELSR1
TJP2
HELLS
PLAUR
DNAH3
PRIM2
RHBDD1
KRT18P28
RNASEH1
AC009237.3
ARCN1
TSPAN1
CARS
YY1
BCL2L1
RNU7-143P
UTP25
DNAJC13
PHLDA1
MINDY2
AURKA
VPS26A
BZW2
PLEC
EPB41L1
DSG2
CYB5B
ZNF267
DEPDC1
MST1R
P2RY2
TMEM87B
ITPRID2
PPFIBP1
YWHAZP3
GRHL2
TAF1B
PDIA6
PPP3R1
PXN
DDX21
GPATCH2
LYPLA1

C1orf112
YWHAZP6
ZW10
ITGAV
ITCH
GAN
ADAM17
ZNF525
MTMR2
SDC4
KIAA1522
METAP1
PTMA
RHBDL2
SPTBN1
DCUN1D1
AC099850.3
WDR43
PRRC2C
YWHAG
TSG101
RNF149
AGFG1
TRERF1
IQGAP1
IGFL2-AS1
JAG1
NDFIP2
MYL12A
SKIL
PGK1
STAMBP
PDCD6IP
TMEM41A
BFAR
TTLL5
AHNAK
TRAF7
AC078923.1
MGAT4B
CGN
ZDHHC3
SPTLC1
HDGF

CSTF2
EIF5A2
TEAD3
HERC4
TRIP12
ZNF28
TCF7L2
AC007128.1
TGIF1
ANXA3
PANX1
MOB4
IGFL1P1
EZR
SLC16A1
SMNDC1
COL6A4P1
AC124947.2
MYD88
FAT1
LIN9
ANTXR2
MSH2
RAB1A
HACD2
MYH14
AFAP1
SGMS2
KITLG
GCC2
YAP1
CASK
CTTN
C16orf87
CDK1
MOB1A
FRMD5
BCAR3
CENPE
RBM7
MELK
GORASP2
SFN
TMEM185B

NUDT21
VCL
CENPN
CIP2A
TP53BP2
TMEM87A
TSC22D2
KCMF1
HOXA13
PLAG1
GPSM2
MUC4
STYK1
MTFR1
B4GALT5
RAC1
FBXO28
NCL
ZNFX1
PIAS3
DYNC1I2
EREG
HIST1H4H
PLA2R1
KLF5
AK4
BARD1
SEPT10
GPRC5A
PRICKLE3
JRKL
ITPRIPL2
ETV3
RIF1
DHX15
HCAR1
EIF2AK2
GJB5
TMPRSS4
LCOR
TET3
FMN1
KCNN4
FOXQ1

AC008440.3
ITGB6
CDK2
PDLIM5
SPICE1
ESRP1
STIL
RNF168
RASAL2
AK4P1
EPS8
BAIAP2L1
RALBP1
CTNNB1
ARHGAP11A
KCNK1
PRMT5
AC103702.2
GSKIP
SLC35F2
NT5C2
SDR16C5
BRCC3
LY75
SP3
HRH1
ADAM10
CDA
ABALON
CBX3
IARS2
SPDL1
DKK1
RHOF
KNL1
TNS4
NCK1
FBXO34
AIDA
MAP4K4
PTK6
RAB22A
PLS1
CAP1

ARHGAP21
CENPI
ANXA2P2
SLC30A6
POF1B
CCNK
RACGAP1
STK26
DUSP11
ZDHHC7
SLC35F5
PIP4K2C
SUN1
HMMR
SLC35A2
OPN3
SLC5A3
MAPK6
EPHB4
PHF6
KIF20B
EGFR
UHRF1
HNRNPF
PGM2
XPO1
RMND5A
FAM3C
TES
TPM3
PLA2G16
BUB1B
DDX18
SPTLC2
BAK1
PERP
KIF4A
TMBIM1
PALB2
MMADHC
AL137782.1
FAM3C2
NHS
CCNYL1

BCL9L
LMNB1
ANKIB1
CDCA8
RALA
MBOAT2
ATL2
SSB
STK24
HIST1H2AI
CTNNA1
AL627402.1
PSMD1
CTTNBP2NL
DIAPH2
LACTB2
KIF11
ZNF185
COPB1
STK38L
SPOPL
CKAP5
FZD6
FNDC3B
LAMB3
AC063952.1
CASP8
PCDH7
MAL2
SP1
KIF23
CDK6
CAPN1
BAZ1A
ZNF143
TAF1A
B3GNT2
PPARG
INAVA
EIF4G1
SLC2A10
OAS3
KIF20A
CHML

INCENP
MIR4713HG
RNU2-27P
BZW1
OSBPL10
AC007128.2
ANO1
ABTB2
WDFY1
ARL5B
SOWAHC
MXD1
CDC73
COL17A1
G2E3
FOXL1
RCOR1
ACTR3
ACBD3
LDHA
E2F7
TBL1XR1
CKAP2L
CEP55
CMTM6
PTBP3
HEATR1
UGT1A10
PLEK2
BLZF1
PPP3CA
ATP11B
INSIG2
DIAPH1
SMC4
NMI
HIST1H2BD
NIPAL1
AMMECR1
LMO7
RAB5A
ATG16L1
FAM83B
YWHAZP2

TPX2
KPNA4
NCKAP1
BCL10
CAPN2
PARP4
SLC25A43
ARHGAP5
ELF4
SPRED2
OVOL1
ARF6
STK3
PAK2
KLF3
TMEM241
ACTR2
ITGA3
ZC3H15
VEZT
NIP7
SNX6
HOXA10
LAMC2
HJURP
IRAK2
AC106900.2
TMEM123
LINC00941
GALNT3
AMIGO2
NMD3
PCDH1
TNFRSF21
EPPK1
ALMS1-IT1
ZNF146
PSMA6
ARHGAP42
MAP4K3
YWHAZP4
CENPF
RAB6A
BUB1

PLEKHA7
SPATS2L
AJUBA
CRYBG2
ASPM
RHPN2
PKN2
BACH1
PDCD10
TMC7
PATL1
SLC35A3
NRAS
LIPH
AHR
PGM2L1
TUBA1C
LRRC1
UBA6
KCTD5
HK2
AVL9
SLK
CAST
IPO7
UEVLD
PSEN1
ADGRG6
PAWR
CHMP2B
SMURF1
PSMD7
NEK2
AP1S3
IL1RAP
CD109
NET1
SLC30A1
MALL
PPP4R1
OSBPL3
PEX13
EPHA2
ARNTL2

HMGA1
NDE1
PRRG1
OXSR1
YWHAZP5
REEP3
STAU1
LASP1
TOP1
NEK7
SAMD9
AC015660.1
SKAP2
TOP2A
DLGAP5
ZDHHC5
RAB10
STRN
IRS1
ARHGAP32
ZNF217
RALB
ARL6IP6
RPE
INPP4B
ITPR3
CTNND1
RIOK3
YES1
TAX1BP1
VPS35
PARD6B
SINHCAF
ADSS
PRKCI
SRPK1
KIF18A
ATP2C1
TGFA
ACTL6A
ASPH
ERO1A
HMGA2
NAB1

CAB39
ADAM9
EFNB2
GALNT5
GJB3
YWHAZ
LAMA3
PRPF40A
TMOD3
BTBD10
FLNB
B3GNT5
ZDHHC20
PIK3CB
CNNM4
MET
KDM5B
SPTY2D1
GPD2
ANLN
CDCP1
AHNAK2
ADGRF4
KRAS
KIF14
AGPS
GJB4
PRELID3B
ITGA6
ASAP2
IGF2BP2
CAPRIN1
MACC1
CD2AP
SERPINB5
MYOF
SLC2A1
CDH3
ITGA2
ECT2
PTPN12
NFE2L3
ACTN4
SLC25A24

	TLDC1	
	FGD6	
	SAT2	Negative
	C22orf39	
	CYB5D2	
	ZSCAN16-AS1	
	GKAP1	
	R3HCC1	
	AC012146.1	
	AC023509.4	
	AC026979.2	
	AC025181.2	
	CIRBP	
	FMC1	
	EGFL7	
	ABHD14A	
	SEC11C	
	AC068473.5	
	SNHG9	
	PRADC1	
	GNG7	
	SMIM27	
	SMDT1	
	LYRM9	
	AC008443.4	
	MRM3	
	GAMT	
	CLU	
	ATP5F1A	
	FGF14-AS2	
	SLC2A11	
	PITPNA-AS1	
	AL353743.1	
	KLHL22	
	AP002360.1	
	AL121601.1	
	SMARCD3	
	AC104825.1	
	AC068338.2	
	FAM69B	
	CXXC1	
	TMEM88	
	AC027644.3	
	AF274858.1	

AC007114.1
SSR4
AP004609.3
ELAC1
AP000757.1
ACAT1
ILF3-DT
ZNF236-DT
TSPAN7
SYNGR1
BEX5
AC100810.1
CLEC3B
AL691432.2
HEIH
DIRAS1
FKBP11
AC133552.5
RAB11B-AS1
USHBP1
ATOH8
RPL3
EXOC3-AS1
INPP5K
EPHX2
CERS4
RIC3
FLJ38576
NDUFAF8
BEX2
GADD45G
RWDD2A
AC068338.3
AL118558.4
THAP7
OTUD7A
AC139530.1
AL121929.2
AL162377.1
PPP6R2
NAGLU
RSAD1
ARIH2OS
AC233723.2

LY6G5C
GTSE1-DT
RAMP2
KCNK3
ANKRD54
AC004825.2
AC142472.1
AC036176.1
BEND5
ECHDC3
PHPT1
BEX1
RASIP1
AC006449.6
LINC00909
BEX4
GALNT16
GS1-124K5.4
CDIP1
TSPAN33
MRPL54
HMGN3
DNAJC28
RNF167
AF131215.7
AC104986.2
AC091271.1
FXYD2
AC027575.2
CNNM3-DT
UBE2D4
ZNF540
AC090617.5
AC026471.2
AC024075.2
SNHG19
AP001160.3
NT5C3B
MROH8
CDC37L1-DT
FUNDC2
DGCR6L
MED9
OLFM1

SLC25A5-AS1
ZNF775
AC156455.1
TRIM52-AS1
AC095057.3
AC069120.1
AL035701.1
HDHD5-AS1
ZSWIM7
AC072061.1
AP000892.2
CDPF1
NUDT18
APLP1
RPARP-AS1
KDM8
AC006942.1
SAYSD1
SIL1
KLKB1
FLJ37453
NPM2
AC106795.2
RASSF8-AS1
MSI1
ZNF793-AS1
QRICH2
NAA38
TMEM121B
CHST10
SPRN
LINC01023
COPRS
AC000068.2
POMT1
DCTN3
SNTG2
RNASEH2C
AC087071.1
NRL
UCK1
CFAP410
AC009560.1
CBX7

NENF
AL513165.1
PTPRS
C1orf56
SLC25A11
HLF
RANGRF
AL118558.3
SCAMP1-AS1
CAMK2N2
RAMP3
USP20
SNAP25
TIMM22
AL355472.1
FAM110D
BRWD1-AS2
CLDN5
ELP5
COX4I2
INTS10
RAB26
TBKBP1
ZNF582
AL136304.1
PDZD4
SHPK
TXNL4A
COQ8A
ATP6V0E2
FAM167B
ENHO
TCEAL2
DPP7
VAMP2
VASH1-AS1
AC074032.1
AL135925.1
TMEM198
PELP1
KANK3
AC124319.3
MIR3682
COQ10A

AC000068.1
AC129507.2
LINC01431
GSTA4
TLE2
NT5M
SOX18
RPS20P22
CERK
COA3
GPR162
YTHDF3-AS1
AC127024.5
USP2
SLC16A11
NPR1
DNAJC30
NEURL1
MAPK8IP1
AC120049.1
RN7SL832P
AC073508.3
RGN
AC002470.1
AL354920.1
GRIK5
RBP7
DANCR
LINC02447
CAHM
AC104113.1
FBXO25
MTERF2
PMM1
ACACB
SLC29A4
PTP4A3
AC138207.3
ATP1B2
GSTZ1
AC079174.2
LRP3
RPL17
DUSP26

OGDHL
LIPE-AS1
AC079848.1
C6orf226
CACNB2
ENAM
RPL3P4
RTN4RL1
ZNF554
AC127024.6
HEXDC
AC026304.1
AC116614.1
ST6GALNAC6
NAP1L2
CCM2L
NAT8L
ZNF667-AS1
AC084036.1
VTN
AC009118.3
FBF1
BX649632.1
C15orf61
ZNF710-AS1
SOCS2-AS1
CCDC106
CD320
RCAN2
NEIL2
AC040169.1
AL442663.3
SH2D3C
AL022328.4
AC098484.2
FAM184A
ATP6V0E2-AS1
FGFBP3
AC007541.1
AC010531.6
RASD1
AC100793.4
ABCA5
AC002059.1

AC093591.2
SNHG25
AL023806.1
AC139768.1
CYFIP2
FBXO10
NRIP2
MOCS2
SMIM4
LINC00476
AL662844.4
MOCS1
AL049796.1
MED14OS
AC064807.2
STK33
SDK1
BORCS6
SLC6A16
C3orf62
PKIG
RPH3AL
ZNF835
SYP
AC022916.1
SLC26A11
ECSCR
RNU4-47P
PRKN
AC092119.3
SURF1
CAMTA1-DT
KCNJ2-AS1
WSCD1
AP000757.2
NCAM1
AC099778.1
SLC22A17
KCNJ11
CHADL
AL080250.1
RUNDC3A
CTNND2
AC008966.1

DTNA
AC025162.2
TBC1D13
SGF29
TXNL1
TMED6
AC135507.1
ATP2A3
NKX2-2
LINC00957
AC034236.2
GRASP
AF186192.1
CAMKK1
AC105020.5
AC009126.1
KIAA1324
AC006511.3
C2CD4B
SNPH
STX8
AC020910.4
AC087752.3
CFD
DPH1
KIRREL2
CACNA2D2
NDUFV3
TCEAL3
SEC61B
AC048382.5
AC025449.1
ZNF667
AL596244.1
NPHS1
KRBA1
AC015726.1
CCDC188
ZNF491
NME5
ZNF467
SLC16A12
MED11
AL645728.1

C18orf21
FXYD6
CCDC178
SCOC-AS1
AL117332.1
EPM2A
GNAZ
CRIP3
SCN1B
AC020765.2
AL158163.2
FAM107A
SERPINA6
DRC3
REEP2
AL358472.2
CASP17P
SGSM2
LINC02001
HPD
LINC01750
AL035071.1
AC016907.2
DERL2
NDUFB2-AS1
LINC00242
AL163051.1
NUCB2
MAPK11
AC069224.1
AC132938.1
C16orf96
AC091965.4
NMNAT3
PPP1R1A
ZFP2
AC009570.1
PNPLA7
RASL10B
DHRS4L1
SRSF12
ECSIT
AC007448.3
AC012640.2

SOCS2
PCSK1N
KATNAL2
FAM104B
AC087521.3
AL137784.1
FXYD1
AP000704.1
SOX17
DPM3
MAGEH1
MAN1B1-DT
C1QL1
TAL1
ZNF771
LINC01715
LINC02106
AC006033.1
ACKR1
FAM117A
AC026367.1
ZNF32
MBD3
METTL7A
AL161729.4
AC064801.1
PDCD4-AS1
KLB
C1QTNF4
SPTB
Z97653.1
PARD6A
AC034243.1
LMO3
FAM229B
TTLL11
KCNH6
IPO5P1
DIRAS3
RUNDC3B
ZNF580
NRG2
AL355877.1
AC083880.1

LINGO4
TSPOAP1
AP002360.3
PACSIN1
NANOS1
ERP29
DNAJC27-AS1
C16orf89
G6PC3
ZNF582-AS1
MIR497HG
AL117335.1
PHYHIPL
NOVA1
TMEM44-AS1
AL109947.1
MT-TC
MIR7-3HG
SCML2
GPX3
TMEM72
AC254562.3
AL590326.1
C1QTNF9
RAB39B
CH17-340M24.3
OGFOD3
AC129507.3
MTUS2
AL451085.2
LINC00092
ZNF671
AL121655.1
LRRC27
AL109811.3
HMGN2P15
Z97989.1
AC106047.1
AMZ2P1
GCAT
MTURN
LINC01952
ACTR3B
EMID1

AC092535.1
HSPC324
ZBED3
AL359513.1
AF186192.2
GPRIN3
RTCA-AS1
AC130343.2
AL354892.2
BRSK2
TCEAL5
CELF3
AL121832.2
AC026150.3
SCGN
AC005076.1
ANKRD16
USP51
TDRP
LINC01315
AC127070.1
SH3BGR
AC073857.1
CHKB-DT
SDSL
MAPK10
CDO1
HOGA1
AL133325.3
ACSL6
CCDC28A
AC084033.3
LINC01985
SELENOO
SNHG8
AL121583.1
AC008438.1
AL021368.3
AC105020.6
LONRF2
AC092535.3
ZNF781
TMEM59L
SMIM10L1

LINC01353
NRGN
RAB3C
EDA
SCN3B
AC046185.2
SLC25A42
PAN3-AS1
AF127577.5
SLC43A2
HIGD1B
PAXBP1-AS1
AL080317.2
PEMT
UQCR11
AC006369.1
MAGEE1
PSPN
AMER3
AC068620.2
ARHGEF15
YJU2
AC062017.1
FAM218A
ACTL6B
SPAG7
TRAM1L1
AL359504.2
AF111169.3
TMCC2
B4GAT1
AL133520.1
MPZ
PYY2
SMPD1
AC006538.1
INKA1
AC093503.1
INA
RGS11
QDPR
MCRIP1
NAP1L5
AC011008.2

DUSP15
AC126283.1
AC015917.2
RAD51C
PXN-AS1
AC124016.2
AL022341.1
KMT2E-AS1
PACRG
LRRC36
ATP2B1-AS1
ELMO1
CAND2
POLR2J4
CDH22
AC092814.1
ZNF596
AC097639.1
MRPL40
IL3RA
ALKBH7
DHRS12
PEBP4
AC112184.1
HRC
AC026979.3
APOH
ATP6V1G2
PCP4
U47924.2
ANGEL1
IFT20
AC015961.2
AQP7
SEMA6C
AC110609.1
AL603910.1
RTN1
ANKRD53
AC016876.1
GAS2
JADE1
AC087500.1
ROBO4

LOH12CR2
TECTA
AC018413.1
AC009403.1
AC021205.3
FAM155B
RN7SL521P
TENT5C
AMIGO1
ZFP3
TRO
KIF1A
TSSK1A
PKNOX2
C10orf143
NFASC
KLHDC9
LINC01089
TMOD1
AC005332.3
BORCS8
AC073316.3
VWA5B2
AC087500.2
AC100803.3
INSM1
TSPYL2
LMO2
IPO9-AS1
ASB16
AC128687.2
APBB1
AL022068.1
AC002511.2
ATPAF2
PAK3
FBXL16
MXD4
ZNF559
GNRH2
AL022329.1
UNC79
HFM1
UBXN8

CCKBR
AC099791.2
U91328.1
AC092666.2
AL021707.1
LHFPL4
C3orf18
ZNF688
AL031775.1
SLC25A27
AL162274.2
UCHL1
KLF15
DCT
FBLL1
C18orf32
AC012615.1
ERO1B
ST7-AS1
AC015977.1
C21orf58
SERPINI1
ARSG
CES4A
ZMAT1
POLR3H
SARDH
AC108673.3
AP001972.1
SYT3
AC005696.4
CALY
YBEY
ZNF542P
AC005790.1
ZNF843
AC012254.5
LCN6
PPP1R3E
CELF4
TRMO
AC068880.4
LRPAP1
DMAC1

INAFM2
SLC25A34
SVOP
FGF12
AL355480.3
AP003721.4
AC023830.3
SERPINF2
AOX1
AL080317.1
DSCAML1
KIF26A
DPP6
EID2B
MESP1
AC005498.3
EFCC1
ST8SIA3
SLC25A4
AC008610.1
AC012306.2
B9D1
LRFN1
LINC00683
PHF24
NDUFS7
AC099522.2
SPECC1L
ABCC8
KCNB1
AC067747.1
GCDH
EMCN
AC004540.2
PIPOX
FAM110B
AC009812.1
SLC8A3
AC007686.3
SESN1
VLDLR-AS1
CACNA1H
AC008035.1
AC096540.1

CCDC159
SUGT1P4-STRA6LP
HCG14
AL391069.3
PAK5
AC027271.1
SLC2A8
TRAF3IP2-AS1
DKFZp779M0652
GPS2
MYRIP
PQLC1
KCNJ5
LINC01481
WNK3
QTRT1
SNAP25-AS1
PFDN5
FAM222A
HPCA
PELI3
AP001267.3
AL358781.2
AC107952.2
ZNF232
FITM1
LRRC4B
SLC1A2
C12orf57
PPP2R2B
PRDX4
NEXMIF
ZNF658
MPP2
MPP1
AC018926.3
C19orf25
RPLP1P6
ZNF204P
IGLL3P
RN7SL336P
FDX2
ZNF414
GABRB3

ITFG2-AS1
OAZ2
CHMP6
PTGDR2
PLAC9
LINC01003
GJA4
CHRNB2
ABHD8
SNRPN
SNAP91
BNIP1
PSMG3-AS1
AL157996.1
NUDT9
AC064807.4
IFFO1
AC026367.3
PCBP3
HCN2
AL442128.2
ASPSCR1
OLFM2
CORT
SSTR3
CDK5R2
MIR583HG
Z95115.1
SHC2
SERP2
CNKSR2
AC008808.2
AL353708.1
PEBP1
RND2
AC012511.1
AC008629.1
VIPR2
C9orf147
PAICSP1
TAGLN3
RPL6
AC025175.1
ANKRD7

INKA2
RLN2
CTTNBP2
DCXR
AC138207.5
ATP5F1D
AL391807.1
PTGES3P2
RGS9
ADGRL1
AL355297.4
AL136295.6
ESS2
KIZ
MAPK8IP2
TMEM179
ETV2
SLC8A2
LINC00271
FMN2
GC
AC099568.2
IZUMO4
ST6GALNAC4
VEGFD
AC004895.1
GRIA2
PNMA8A
CCL14
ZNF594
AC005899.7
HAAO
AC051619.5
SALL2
NRTN
TMEM63C
AL133343.2
AC005920.3
SERGEF
DSCR9
ADGRB3
AC116913.1
AL162231.1
GNAO1

PNMA8B
SLC48A1
CRTC1
GRK4
CACNA1A
FITM2
AC087392.5
CHST9
SSBP4
SCG3
MBLAC1
RIPPLY2
GPRASP1
ELP2
IL6R-AS1
PNMA3
CLGN
THA1P
AC007066.2
AP000255.1
AL031666.1
NKX6-1
AC080013.5
AP001972.5
AL138762.1
GSTM2
LIFR-AS1
NDUFA6-DT
ZGLP1
ZNF778
TNS2
SLC38A10
FBXO9
AL158071.2
AC026691.1
ADCY5
ZDHHC15
PSTK
ADARB2
GYPC
DUS3L
AC093249.6
SOBP
ZNF18

SPTBN4
CPB2-AS1
AL158163.1
AL157813.1
KCNMB2
AC007220.1
FFAR3
KCNA5
KIF12
GPR150
BSNDP2
AC009113.1
AL031665.2
CA4
AC012313.2
AL161729.3
SCAMP5
COPS3
FAR2P2
TMEM175
ZNF428
AC003035.1
SYT4
AP001627.1
XKR7
AC023632.2
NCALD
BCL11A
PTPRN
RAMP2-AS1
AL035563.1
LTC4S
AC009506.1
FGF17
AL158063.1
METTL16
CTNS
SLC7A2
ARVCF
SOD3
GHRL
ZNF674-AS1
MLYCD
AC005498.2

LINC01144
BSN
TMEM255B
AL390067.1
DLGAP3
AC096733.2
SLC25A18
AC011445.2
SDF2L1
TMEM132C
CRAT
AP3B2
HRAT5
C3orf33
AC007666.1
AC009088.1
ITGAE
AL451085.1
AC008403.3
AP005131.7
PGBD5
PRRT1
SH3GL2
FYB2
AL583856.2
LINC02202
URB1-AS1
AC005837.1
AC004696.1
BCAM
AC005785.1
AP003392.6
AC008115.4
RNU6-8
SAFB2
BRF1
AC022098.4
AC008543.1
AC005229.4
SPATA41
SEMA3G
LINC01942
ELAVL4
AC026801.2

AC104985.2
SLC39A3
EVL
RPL17P36
NIFK-AS1
AC002398.2
TRMU
ZNF853
CYP51A1-AS1
AL137002.1
ZBED3-AS1
CYP4F32P
AL049612.1
C19orf73
FAM180B
AC026150.2
GCK
AC138207.8
FAM167A
ARMC5
BLMH
NSG2
AC107375.1
MDP1
BTBD6
LINC02361
RGMB-AS1
AC010501.1
C1orf127
CCDC92B
UNC80
FXN
ZNF483
CXXC4
ARX
LRMDA
LCMT1-AS1
LINC01531
ZSCAN18
AC007663.3
AP001486.2
AC233976.1
RNF212
AC016582.3

AC063948.1
ICA1L
AL355803.1
CYP46A1
HIRIP3
SEPSECS-AS1
CPLX2
AL136531.1
AC011511.1
MHENCR
AC009163.6
F10
AL022332.1
SCD5
AC005393.1
CLEC14A
CASKIN2
AC093642.2
TACR1
AP001412.1
POMC
SHANK3
AC007950.2
AC016924.1
HMGCLL1
A1BG-AS1
AL138966.2
AC010999.2
MMRN1
ASB14
AQP4
AC005034.4
SMAD4
AC007292.1
AC009309.1
AC010226.1
MBOAT4
CNIH2
POLI
TDRD10
H1FX-AS1
KCNJ8
AC019131.2
THSD1

AL596442.2
ATRNL1
AC037459.2
NTRK2
AC097359.1
AC091825.1
RIMBP2
SIRT4
SPART-AS1
AL390208.1
FO393419.3
CASTOR3
AL358072.1
MNX1-AS2
RASA4
AL139353.1
AC005224.1
DHRS4L2
GABARAP
AL645940.1
AL135744.1
PHACTR1
PKMP3
NIPSNAP3B
NR0B1
GIMAP1
PSD
AC002511.1
WDR83
AL512625.1
AC125494.2
HDHD2
RPL34
LRRC2
CDADC1
AC044849.1
AC008494.1
OXLD1
KIAA0319
AC079089.1
IL11RA
AC091153.3
MINCR
PLPPR1

FAM189A1
SURF2
NDUFB2
AP002360.2
LINC00900
AC002451.1
MEG9
CPEB1
FAM228B
SRRM3
FRRS1L
XPA
LINC00484
Z69706.1
ZC3H10
AC097461.1
AC093635.1
MIF4GD
FBXL15
RFX6
SV2B
AC012603.1
AC012158.1
RADIL
RFXAP
NRCAM
BCDIN3D-AS1
ZNF503-AS2
AC016876.3
AC137767.1
PRKAG2-AS1
SKIDA1
SCG2
RPL21
ARRDC1-AS1
AC034198.2
ZNF821
AC108134.4
DPY19L2P4
IQSEC3
AC103736.1
KIF5A
VLDLR
C16orf86

GDAP1L1
SRP14-AS1
KCTD2
ROM1
AC074044.1
AL353719.1
RPSAP9
AL023803.1
AL157935.2
GNG11
AC027682.6
UCN3
NUAK2
OAZ1
ZCCHC18
LINC01534
EFR3B
AC011933.3
PPM1E
CDK9
AC015802.5
PWAR6
FKBP2
MTMR7
AC005225.4
C17orf107
AC023421.1
SLC12A5-AS1
ECI2
PANO1
CRMP1
ITGA7
POU6F1
TOR2A
TUNAR
MT-TL2
ARHGEF26
AP005205.2
WIPF3
TMEM254-AS1
AL008582.1
AC015819.1
AC022413.1
RTL5

WDR83OS
AC027279.1
LINC01816
NDUFA11
NOL4
C9orf43
SNHG7
INSYN1
BX649601.1
AL133353.1
DDT
C5orf38
SNRNP25
NUPR2
XKR4
TSTD3
BEX3
NIM1K
C19orf81
SGMS1-AS1
KIF6
SEZ6
ZNF433-AS1
LINC02044
C9orf139
AC011477.1
TTLL7
COMTD1
AL596202.1
KCNK17
LINC00602
AC083798.2
AC106795.5
LINC01484
CXorf57
ALKBH5
RASSF1-AS1
ESRRG
PAH
CTSF
GRIA3
NRSN2
AL391834.2
ISCU

SNHG20
KCNH2
EEF1DP4
ANK2
AF131216.4
AL355377.2
RBM26-AS1
COQ4
C6orf163
DICER1-AS1
CFAP70
AL391261.4
AC007495.1
METRN
ACSM6
EBF4
HS6ST3
PDCL3P4
TPST2
MYT1
SFTPD
LRRN3
ABHD17A
CD81
A2M-AS1
CDKN2AIPNL
AC005329.1
NRXN1
ZNF602P
PLVAP
AC104333.3
AC021242.3
AC079331.2
AC092437.1
NUTM2B
ASTN1
AL139274.2
LINC01529
AC133552.2
AL139286.2
ST18
RPL17P34
TRMT9B
SGSM1

RBM11
AC018521.5
BAALC
LINC01238
OR7E47P
PNMA8C
VGF
AL118516.1
PIK3CD-AS2
PDCD4
TPGS1
TMEM170B
SSTR2
LINC02147
NDUFA13
CTB-178M22.2
RAB3A
AC069209.1
AL008723.3
AC078925.4
ZNF10
GOLGA7B
AC115223.1
AZIN1-AS1
ARHGDIG
GPLD1
RPS27P25
PRRT3
PRPH
HINT2
FBXO15
AC015909.2
LINC01424
FAR2P3
NECAB2
ZSCAN1
ALG14
NUDT2
SUZ12P1
AC091152.4
LINC01963
TNRC6C
AC053527.1
CADPS

AC012640.5
AC005911.1
AL031658.2
ASMT
MRPL34
NPIPP1
RNY3P8
PDK4
AC120498.10
A1BG
SEZ6L
AL121672.2
AC026367.2
FEV
MIR548AA2
CBFA2T3
CAMK2B
SRR
AC009974.1
CLEC1A
HSD17B14
ADM5
AC002347.1
SNCA
RPL9P29
LINC01014
DBH-AS1
AC018638.6
SORCS1
AC121761.2
KRT222
LY6H
RWDD1
AP002748.3
AC103746.1
AL356740.1
MTDHP3
EML6
TIMM13
RNASEK-C17orf49
DDX25
AC012360.2
SEC31B
ANKS1B

AC013472.3
PRKAR2B
AL161421.1
CRELD2
UNK
AC136628.3
FAM135B
AC131159.1
STX17-AS1
LINC00334
AC092634.3
SNTA1
TMEM121
AC027682.4
MMAB
UNC13A
AC092535.2
DSCAM
SF3B5
MCOLN1
TEX38
ZNF181
AC138965.3
DCLK2
AL136040.1
AL161452.1
AC006012.1
PIK3IP1-AS1
AC024575.1
AC134043.2
PCSK2
AC005962.1
CCR10
CAMTA2
AC016820.1
GOLGA8VP
WDR17
AC119403.1
CERS1
LINC00663
LINC00987
AC087501.4
CD99L2
FLJ31104

TAF6L
AQP1
CCDC110
AC004540.1
NUDT9P1
AC114271.1
INTS6-AS1
ADRA2C
AL161658.1
AF131215.6
RABEP1
F11
LINC02193
RPAIN
BNIP3
SLC6A17
AC010491.1
NLRP1
AL358852.1
AL512408.1
MAPK4
ASPDH
AC011290.1
OXER1
CTXND1
RXRG
STKLD1
AC078846.1
AC079385.3
AC140481.1
SORBS2
SMAD5-AS1
ERMARD
TMPRSS6
AC068888.1
RPS19BP1
ARHGEF7-AS2
HSPB6
RPS18P9
ZNF286B
MAP6
AP002990.1
GNB3
HCST

AC079414.3
SNHG14
AC007687.1
RPL23AP67
AC103858.2
TMEM132D
ADGRF3
AC008759.3
MIR126
DYNLL2
UBAP1L
PDE2A
REM2
AC105277.1
SCN3A
AJM1
TMEM178B
ANAPC2
GPC5-AS1
BTBD17
AC022400.7
KIF5C
CFL1P6
AC010997.3
GNG2
SFRP1
ZDHHC24
UBXN6
DNAJC4
AC114781.2
AL122008.3
NRROS
RNF180
TOMM7
AC087294.1
FLJ37035
AC138356.2
AL355001.2
ATP5PF
LINC01586
AC016705.2
DYRK4
AC064836.3
AC022893.2

STXBP5L
DPH7
AL845472.2
DNAH9
P2RX1
KLF2P2
AL592424.1
GLS2
FZD4
CCDC62
MED16
SLC17A1
AC079140.3
GTF2IRD2
SPA17P1
AC007938.1
AL450326.2
CDH10
S100A1
LINC00870
FN3K
SNCB
ELOVL4
DUX4L27
LINC02600
NGFR
WRAP53
4-Mar
AC016355.1
AF186192.3
AL023881.1
PRPSAP2
PGM5P4
AC005670.1
PALD1
LINC01016
SEPT5
AC005696.1
JPH3
DOCK9-DT
AC096734.2
FNDC5
RBP5
AL357079.1

ZEB2-AS1
CYB561D1
SUSD4
HDAC5
AC008608.2
ABI3
TMEM145
MTMR3
PGM5P4-AS1
OSGEPL1-AS1
MAMLD1
PCSK1
STAT4
VN2R17P
SLC5A4
TUBD1
LINC01927
DBIL5P
FAM120C
AC016575.1
TPTE2P1
RNF130
CD36
NEURL4
ITIH4
AC007029.1
AC004846.2
AC005695.3
TAT-AS1
S100B
AP001528.3
SYT6
AC021755.3
CPEB1-AS1
AC124242.1
ZNF709
PSMB6
AC092134.1
IRF2BP1
RAB9B
MIR600HG
DPH5
CPT1C
ARNT2

AGBL4
IRX2
IRAK1BP1
MT-ND3
AC008121.2
AL671710.1
SHBG
AL161911.1
ZNF625-ZNF20
LYL1
ZNF433
AC020892.2
PIGBOS1
AC008280.3
TBX2-AS1
FRAT1
REPIN1
AC012409.1
AC117489.1
C12orf60
ING5
SCRN2
AC073655.2
MRLN
CHGB
KCNN3
AC010864.1
ZNF358
AP001462.1
NOVA2
AC009690.1
WFIKKN1
AC092802.1
AL034550.2
TMEM150C
AC007485.2
ASB16-AS1
NDUFB8
SDHCP4
AC002467.1
AC116407.1
ENTPD3-AS1
RPS28
ZNF213-AS1

PPP5D1
STPG3-AS1
NOP53
RPGRIP1
C17orf51
SETMAR
AC105339.1
HYI-AS1
CARMIL3
MRPS11
AC068620.1
MYH3
MIR670
SPACA9
MAN1C1
DPH6-DT
AP000254.1
LINC01146
MAST3
SMIM8
ZDHHC22
AL161938.1
NAXD
ZNF271P
AC020558.2
RTN4IP1
CACNA2D3
CACNA1B
HEXIM2
AC018647.1
AC084824.5
ART3
ADGRG5
LINC00643
KCND3
AC244035.1
AC112219.2
SCARF1
AP5S1
AC013356.1
REM1
AC106820.3
NPDC1
AC010175.1

CDNF
AL365194.1
AL136368.1
ENO1-AS1
LINC02148
GDF9
SETBP1
AC125257.1
DENND2A
CBLN1
AC093510.2
AC010478.1
SCG5
CELF5
CORO2B
SHISAL2B
MIR339
AC012645.1
CRADD
AL355472.4
AC106795.3
ZNF98
B4GALNT4
CCDC13-AS1
AL356488.3
CCDC13
TMEM86B
HAGH
GPRASP2
AL008729.1
CUTA
ATCAY
AL158823.1
TMEM151A
AL022328.1
PCDHB4
AC106886.4
SEPT1
SLC46A1
AL031963.3
AP001062.1
AL133551.1
AC139495.1
PCAT19

AC079766.1
PRR22
ZNF71
ADCY1
FTOP1
AC107464.3
MAPT
SLIT1
RPL7A
GPR142
FAM186A
CLEC9A
SNAI3-AS1
AL162171.1
AC025871.2
AC233723.1
SLC12A5
CA11
GARNL3
AL139125.2
RAPGEF4
CCT6B
AL731566.2
AC010969.1
RPL5P34
HDAC10
AC008758.4
MRPS26
AC020558.1
LINC02389
KSR1
AL590999.1
AC067838.1
RPL21P89
WASF3
SMIM10L2A
AC079630.1
SLC7A14
BIVM
ADD2
AC104211.1
UNC119
AC079322.1
TP53I13

MIS18A-AS1
MATK
TXN2
RIIAD1
MDH1B
CST3
RABGEF1
PRAF2
AL137230.1
AL031186.1
AL138767.3
AC012510.1
CCNA1
KCNA2
MRO
MAP3K15
TMEM151B
AL390783.1
AL359258.3
PPM1K
AC092720.2
AC034229.4
Z93241.1
SLC6A4
AL445250.1
PIAS2
AC079305.1
TUBGCP6
MCOLN3
TTYH1
AC104109.2
LINC02559
AC126177.7
SUCLA2-AS1
AC007192.1
NEUROD1
RBMS3-AS3
SERTM1
WNT4
KCNMA1
HHATL-AS1
CHGA
AC084357.2
AC135050.6

NKAPL
AC008669.1
PHF21B
AVPR1B
LINC01829
AC005154.2
AC105942.1
DTX3
AC245140.1
FAM163A
SSPO
AC022417.1
AC025271.4
GZMM
SNAI3
AMPH
AD000864.1
AC112236.2
SPPL2B
SCRT1
PART1
SGSH
KIF19
HIRA
NAP1L6
SPX
CHST12
SMC2-AS1
AC021752.1
AC244669.2
AC127070.2
NUDT7
CYP2C8
BBS5
AC025048.2
AC015961.1
AL391261.2
AC008736.1
AC007000.1
SERPINA10
ALKAL2
MSRA
AC006238.1
AP000802.1

TCF15
CTXN2
AC005775.1
RN7SL268P
ZNF429
AC092574.1
AC010323.1
AC010980.1
RPL26
GAD2
AC132872.1
AJ011932.1
OSBPL6
EFCAB6
SEMA6A-AS1
ZNF814
MAP6D1
JAKMIP1
GOLGA8N
PDE1C
AC100812.1
TMEM256
SNX22
PLCG1-AS1
LINC02101
MT-CYB
FLT4
UBE2Q2L
AC091849.2
PCDHB1
RANBP3
LRRTM2
DNAJC3-DT
AMHR2
MPC1
SARM1
AC093278.2
TUBB4A
ZNF503-AS1
CRYBA2
FAM184B
AL596094.1
AP001437.1
STK24-AS1

RNU4ATAC
AL133299.1
AC012254.3
AC040896.1
MIATNB
AL158196.1
CCDC180
AC073911.3
AL158071.3
PDXP
ARRB2
LMF2
AQP4-AS1
AC013468.1
KCNH3
UQCRB
CLUL1
Z69890.1
TOLLIP-AS1
ZNF426-DT
AC008808.1
ALDH5A1
DMAP1
AC010331.1
TUB
AC006277.1
AL589765.1
ZNF396
MIR320D1
AC104985.1
LINC01574
GUSBP1
TNR
TMEM131L
APOO
AC009185.1
CADM1
COX6CP13
SLC14A2-AS1
AL121899.1
MKS1
AL359853.1
ZNF682
TSNAXIP1

EXTL3-AS1
PBX3
ANKHD1-EIF4EBP3
AC092117.1
LINC01128
TCEANC
ULK2
AC011477.7
CCDC157
CADM3
LINC01465
SELP
AL604028.1
KCNJ16
WNK2
LINC01976
RN7SL73P
ASPA
SH3GL3
ADCK1
AC022415.1
AL162419.1
CAMK1
AP005671.1
AL035250.1
LINC01664
AL353708.3
F7
AC004947.2
HSD11B1L
DLG2
TFEB
GEMIN7-AS1
LINC00339
SPATA45
GJC3
CABP7
CTNNA2
BRINP2
AC009630.1
PINLYP
SNHG21
PRR34-AS1
LINC01695

AC116003.3
KIAA1328
HIPK1-AS1
AC090948.1
AL158055.1
AC011825.4
F11-AS1
AOX3P
AC012254.1
ADGRD1-AS1
ARHGEF26-AS1
AL138960.1
LGI3
SHISA7
MADCAM1
MTATP6P1
AC027807.2
NDUFB7
JAZF1-AS1
LINC00261
PLPP5
AL031432.3
PPIEL
FAM227B
TUSC1
HOMER2
AC104117.3
AC090114.2
AL358933.1
AC009407.1
CYP4V2
AC073343.1
CECR2
PROX1
AC004221.1
RPL5
SMIM32
CPA5
RASA4DP
MAST1
CFP
AL139130.1
TBC1D24
AC006116.8

GPR75
AC139713.2
GGA2
PAX6
BSN-DT
LHX6
C7orf26
ALB
AC016590.3
AC012085.2
RFPL3S
SDHAF1
SETDB2
EIF1
AL136295.7
CISH
KCNJ6
AL136038.5
PLD6
NEU1
ST3GAL5-AS1
CXorf36
PEX5L
AL355297.3
TPPP
FLT3
AC063943.1
AP001636.3
MKKS
C2orf40
TBXA2R
ZNF76
AC115284.2
AC243830.2
LINC01779
CATSPERE
ATP1A3
OCM
ACADL
ASXL3
AF279873.3
AC126177.6
GLIDR
AC024257.5

GALT
AC231981.1
HDHD5
MAPK12
AC116337.3
TCEA2
AC107031.1
AL034374.1
ZNF862
PTPRN2
IGFN1
FSTL4
GDF11
FAM200B
RN7SL771P
CCL21
AC120498.8
AC100771.2
ZACN
AL513534.1
RALYL
CATIP
MBL1P
GRB14
AC012629.2
AC245297.1
DRAIC
CCDC141
AC006254.1
FAM181B
RAI2
AC124045.1
PIGP
C21orf62-AS1
AC004471.2
GPR146
PRR26
AC093206.1
AC026464.1
PCDH10
EID3
AL022328.2
AL590787.1
ERVK9-11

FFAR1
NRSN1
KL
AC139795.3
TOGARAM2
U62317.3
PICK1
AC064799.1
POU6F2-AS1
SCNN1D
AL080317.3
C2orf73
PARVB
ZNF157
RPS3A
NEBL-AS1
AL021328.1
AL357146.1
AC024580.2
SLCO5A1
SYT5
AC026748.1
EPHX1
IRS3P
ZNF397
ZNF763
CBX6
ZNF79
MTHFD2L
ZADH2
PLCXD2
CPE
TIMM21
CNPY3
NLGN1
AC090241.3
AL359764.1
AC110285.2
TENM1
TVP23C
RET
AC104794.4
P2RY11
CXorf58

PAXIP1-AS1
HDDC2
LINC01374
POU6F2
RPS6
FOXP2
AC099521.1
RSPH9
DNAJC18
GRM4
OTOA
ZNF583
SHE
GPR135
C1QTNF2
KHDRBS2
GNMT
S100Z
RASL10A
C8orf86
AL031847.1
GVQW3
RGS22
FGL1
ROGDI
MIR153-1
MRPL57
CETP
UBAC2-AS1
WDFY3-AS2
AL135905.1
AP000345.2
AC022107.1
LRRC10B
AC013474.1
AL022476.1
CXCL12
CLCNKA
COPS9
ZER1
TMEM74
AC015802.3
AC106820.4
CARTPT

SLC25A30-AS1
KCNAB1
AL020996.1
AC066612.1
OXCT1-AS1
AC024361.1
GLTPD2
LRRC4
KARSP2
LINC01354
AC005332.1
AC008752.2
LINC01063
NCOA5
AC006116.6
ATP1A1-AS1
TMEM8B
SBK2
GNAL
LINC01152
LINC02586
ZNF625
LRRC37A5P
COL26A1
LYSMD2
SLC12A1
AL022069.1
VPS33B-DT
AC104123.1
CNBD2
JPH4
TMEM61
AL161729.1
KCNB2
MLXIPL
HPF1
GTF2H5
SLC43A1
Z93930.3
AC005696.3
AL160286.2
AC012617.1
ITPR1-DT
TMEM196

FAM57B
SEPHS1P1
RPL23AP49
ADAMTSL2
CRHBP
AC013460.1
AP003559.1
AL391883.1
CKMT2
AC145422.1
HCG2040054
PLD4
LNP1
RGMB
DZIP1
AL117336.3
AC068870.2
JAM2
MAST4-AS1
LINC00644
ST3GAL3
SCML4
AL592494.3
RPS15AP38
AL132655.2
RPL17P43
ADH1B
AL442663.4
THAP7-AS1
AL356218.1
AC004263.1
AL009178.2
AC135178.5
LINC02593
AC008124.1
FICD
MEG8
NDUFA2
AC009283.1
MYOZ1
BX679664.3
FGF14
ZC3HC1
CD34

AC006116.10
AC147651.1
AL353743.3
RXRA
AC073896.2
AC016590.1
KCNJ6-AS1
AC006077.2
AC021755.2
ASB4
ACBD4
C7
PPP4R4
AC055758.2
NPPA
CTBP2P6
AC016885.3
AP000894.4
FTCD
ADGRA1
PCED1A
DYNC1I1
ADRB2
CHST8
C5
CCDC28B
AC114546.1
UTF1
AC025682.1
ZSCAN2
NATD1
CYYR1
AC087742.1
AC012508.2
AC092296.2
ISCA1
TTC41P
AC107871.2
ELOVL2
PIN1
8-Mar
LRP2BP
WASH6P
ZNF837

RNU1-132P
EFNB3
DPY19L2
PGBD1
SYT7
PAUPAR
AL354950.1
ACSM5
AC007221.1
MUM1
MPP6
AC073896.3
AP000347.2
SLC4A8
AC020928.2
AC099684.1
AC226101.1
AL136162.1
SLC14A2
FAHD2B
MARK1
MBD1
AC005682.1
AP001626.1
AC005730.2
CEBPB-AS1
LRRC37A3
SEMA6A
LINC02285
PLPP1
LINC00167
AC087752.4
PMF1
AC010359.1
ZNF660
AC023509.3
KCNK16
AL137784.3
TRMT10B
AC069213.1
LINC01018
U2AF1L4
AC009084.2
AL844908.2

DNAJC9-AS1
AC010809.1
CADM3-AS1
AGTR1
CCNT2-AS1
ZIM2-AS1
SEPT7P2
PAIP2B
PLA2G6
AC004687.1
ADPRM
P2RX5-TAX1BP3
GIMAP7
AC091390.4
AL162412.1
PRKAR2A-AS1
GAL3ST3
TMEM220
AL035427.1
MIR762HG
AC015726.2
FAM53B
LINC00612
SOWAHA
SYNGR4
KCTD16
AC005355.1
AC011239.2
LIN7B
AC007563.1
AL031595.3
C6orf203
ZNF727
AC109454.2
PGM5
AL589765.7
AC090061.1
TCP11X1
AC087457.1
AC010809.2
AC007786.1
PABPC1P7
OXGR1
AC055758.1

REX1BD
C9orf66
GPR148
USP6
JAKMIP2
FAM83C-AS1
AL160270.1
AL136366.1
SNAI1P1
EBF1
RBM22P2
AC073573.1
PRR18
ST20-AS1
AC004877.1
AC124319.2
EXOC3L1
UBOX5
SNTG1
ACTG1P17
ZNF22
PCDHB5
TEKT3
AC090425.1
HS6ST1
MEG3
AC106886.1
MTUS2-AS1
AC112187.3
KAT14
AP001094.1
NUTM2HP
AL021937.3
VPS37D
AC012313.5
AC010469.1
HBA2
ASIC3
CYCSP8
AP003486.1
GJD2
ADGRV1
DZIP3
PDE8B

SCX
SLC1A4
MIR670HG
KLLN
CFAP53
RPL38
C11orf71
F8
ARHGAP24
KCNJ3
MPDU1
FOCAD-AS1
AC112204.3
SLC38A3
UCHL1-AS1
ZNF767P
ADCY4
C22orf42
APOBEC2
AC055822.1
TTC23L
PI16
PTGDS
AL512604.3
AC087623.3
AC104836.1
THEM4
GSTA7P
AC106886.3
NAP1L3
RFX3-AS1
AC018638.7
SLITRK2
KCNC1
VWA8-AS1
AC123912.2
AC022098.2
AL133325.2
MVB12B
AC083964.2
ZNF691
ZCWPW2
AF287957.1
AGBL5-IT1

AC010201.1
ARMCX2
ANAPC16
PAXX
SNHG10
AC069281.1
ZNF804B
AC116609.1
AC011477.2
BX284668.2
AL022337.1
AL161772.1
NAV2-AS2
SLC35F1
UOX
AC008972.1
LINC00908
AGT
LINC01311
ANKRD36BP2
SLC25A6
COPE
UBE2QL1
AC011444.1
LINC00535
EDF1
MIR548AN
ARL16
LINC02012
AL138688.2
SCARA5
AC005014.2
MESTIT1
ZNF732
AL356599.1
TRIM50
VWC2L
IER3IP1
AL136295.2
ANGPTL5
TIE1
PPT2
AC092691.1
GSG1

IDNK
MYL7
AC004803.1
RFESD
AC089999.2
DPM2
AL021878.2
CECR3
EIF3LP3
AC008467.1
AC008667.2
AC099343.3
GTF2IRD2B
FGB
AC107032.1
ZNF653
SARAF
PFKFB1
LINC01625
AL022069.2
C21orf62
UNQ6494
AC109322.1
RPL26P6
CUBN
AC137630.3
FRG1-DT
ACADVL
SNAPC4
ATP6V0A1
UROS
TBCC
AL135960.1
LINC00989
MGAT4C
PHBP20
LINC01160
AC026992.2
PLPPR2
RGS7
KRT8P43
AC131097.4
SV2A
DNASE1L3

FGFR1
SLC25A53
AC005759.1
ADGRG2
AC003102.1
AC010247.2
TRIM60P17
LINC00671
GRK1
AC024243.1
LINC01549
RNF157-AS1
AC021549.1
HAUS7
MT-CO2
TXNDC15
LCN10
AC112722.1
TSPAN11
LINC00844
LINC02427
ATPAF1
RHBDD3
MT1F
AC015908.3
IGFLR1
LINGO3
ZNF280B
TMEM220-AS1
USP27X
AL138831.2
MYCT1
CFC1
AP001160.2
TXNRD2
AC015656.1
AC104590.1
CRYBA1
AC027348.1
KLHDC3
AC007637.1
AL359198.1
RPL12P47
TNNT3

AC105074.1
CLYBL
IMMP1L
AL022324.2
HIGD2A
GPD1
CD300LG
KRT27
RASSF4
SCAND1
AC006994.2
AC063926.1
TCTA
APOM
AC124916.1
AC008494.3
GGTA1P
AGBL3
AL590705.1
AC126773.4
AC107027.1
CYP4F24P
PRCD
KCNN2
ELN-AS1
PPP2R3B
SWSAP1
LGI2
MYO16
AC120024.1
LETM1P2
DNAI2
AC131025.1
CXorf67
RNU6-760P
LGI4
AC016885.2
KSR2
RNF166
MAP1LC3A
AL133464.1
AL359091.4
AC242842.1
LINC01562

C2orf42
AL136980.1
AC073464.1
TAT
NPAS3
TSHR
GFRA2
RPL7P60
AC097376.2
TATDN3
C1orf122
FSD2
C19orf70
AP001646.3
KLRG1
GALNTL6
AC127024.3
DTNBP1
AC093458.2
AC010980.2
AC009065.4
LAMTOR5-AS1
AL157756.1
KIF9-AS1
FCN3
SATB1-AS1
DAPL1
CCL16
AC087623.1
AMDHD1
OSER1-DT
BX664727.3
MAP2K6
RPL21P134
LINC01284
PLPP7
ASCL2
PPP1R3F
AL355994.2
CCER2
CCDC69
AC104187.1
AC008543.5
SDHAP3

PISD
AL845472.1
AC004854.2
AL161719.1
AC114684.1
LINC02197
AL583785.1
LINC02352
AC087463.1
TSPOAP1-AS1
LINC01554
MCF2L
AL606970.1
PPCDC
FGF12-AS2
AC104051.2
RBM20
AC135983.4
AC098820.3
AC055764.2
IGSF22
AC018755.3
NAMA
AL035420.3
ACAP1
AC079610.1
AC011477.3
TMEM201
AC006512.1
AC104806.2
AC010326.1
ZDHHC11B
AC100847.1
TP53INP1
AC010719.1
AC004449.1
KLF2P4
IL17D
AC129507.1
AC127002.1
ACOX2
ZNF497
AC022079.2
BX322562.1

CLN8
AC026369.1
USE1
CRY2
ZBTB16
APBB3
INTS9
CADM4
AC026741.1
ZNF676
SYNE1
SLC9B1
AL513542.1
AC107983.1
POLRMT
AP003117.1
AL021707.6
SERPINE3
NME9
AL512652.1
AC012467.1
SEC14L5
LINC01370
AC104667.1
SFXN5
AC135178.1
CPNE9
CPQ
Z97832.2
HPN
AL035461.2
PCYT1B
AC090587.1
NDUFA7
AC011450.1
AL355102.4
LINC00868
AC048351.1
GLIPR1L2
TRAPPC1
C8A
LINC00315
AC135983.3
AL355432.1

FAM174B
AL022324.1
CYP39A1
NPY5R
EML5
AC009318.4
NDUFB11
LCNL1
FEM1A
KLHL32
AC001226.1
KCNG1
ZNF747
AC124319.4
CCDC3
C17orf100
EZH1
VENTX
Z82206.1
EFCAB8
RNF182
AC040918.1
AC010327.4
AL050341.2
AL133279.1
RTP5
ARC
KLHL10
PCDH17
MIR3671
DEFB124
AC121338.2
C14orf180
CCDC30
RPL10P11
TDRD9
ZBTB48
AC012360.1
BATF3
AC013400.1
SMTNL2
RAB11FIP3
PLIN5
SLC4A10

SLC38A4
AC026803.2
TMEM221
ANKRD65
RPL9P9
AC136632.1
DACH1
GRM6
AC140847.2
TMEM271
RNA5SP103
AP006222.1
FAM153C
AC239367.3
SELENOM
AP001363.2
AC022098.1
AC040169.2
AC097537.1
SLC45A2
AC091982.3
BCDIN3D
AC118553.1
MST1
B3GALT1
RRAGD
AL450384.2
FBXW4
MAT1A
AC020928.1
AC123768.3
WDR91
AC114341.1
RLN1
PRR34
AC015967.1
AC008013.2
SLC25A14
PATZ1
HBA1
SCN4B
AC010307.4
AC007349.1
FLG-AS1

AL096711.2
AL391684.1
B3GAT1
AL353622.2
AL009181.1
AC018845.3
RNU6-644P
AP000346.2
CHRDL1
AC091925.1
AC004477.3
U91328.3
AC073111.1
Z83847.1
LINC02495
AC097468.1
ECHDC2
GOLGA8M
AC108704.1
AC010136.1
PLA2G4C
GPR61
SYN1
PPP1R42
AC007938.3
AL731568.1
AES
HCFC1-AS1
MRM1
AL096816.1
ATOH7
ZNF568
RNU4-14P
COX7A1
AC005899.3
GOLGA8H
AC005332.5
MFSD6L
ATP1A2
AC243562.2
CHCHD10
GAPDHP76
PTPRT
AP003774.1

AC025262.2
AL592293.1
ATAD3C
AP001528.2
LINC01341
NRXN2
AF111167.2
LCN12
AC087289.2
CCDC151
CAVIN2
AC068522.1
LINC00316
AC091153.1
AL359317.2
LPL
DMGDH
TVP23A
AC087289.3
SEMA6D
TBC1D10C
ZNF460-AS1
MT-TS2
ADCY10P1
PCDHA3
LINC02289
AL162426.1
FAM201B
AC015813.5
RPSAP70
AC104785.1
AC133963.1
SNORA11
C3orf49
AL035696.3
TRIM9
AC067750.1
FHL1
AC010913.1
LINC02458
SELENOKP1
AP000812.1
AP001630.1
AC016773.1

ALKBH4
NAT16
LINC01634
AC004812.2
RASL11A
FLRT1
FAM20A
SCN8A
EPX
AC113554.1
LINC02308
AL590302.2
AC004148.1
GPM6B
AC015977.2
MIR3936HG
AC007780.1
SPATA7
ARL5C
HDDC3
NACAP8
ZNF831
SLC23A2
PCDHA1
AC084064.1
AC118757.1
AC145207.5
AL513548.3
AC073195.1
GABRG2
DDTL
AC134682.1
TSSK6
AC139749.1
AL691403.2
RPL6P27
PPIAP4
PCLO
LINC00412
LINC00907
SRCIN1
LINC00311
RPL21P28
AC004012.1

S1PR1
AC007881.3
LINC02572
RNU1-70P
SLC27A5
SIDT2
LINC01535
CERS3
AC051619.6
IL24
Z97055.2
NPW
BLOC1S1
AC018926.1
AC012313.8
HMGB3P32
MIR7-3
TMEM205
AC106779.1
AC063926.3
DMRTC1B
TBC1D29
TUSC8
AP001533.1
FP325317.1
LINC01132
AC068831.5
LRRTM4
SNORD104
HAO1
AKAIN1
REC8
AL583834.1
AL157788.1
NPAP1
AL731557.1
TMEM80
AC055876.4
Z97653.2
AC005697.1
AP002812.5
AC008641.1
NF2
CEP112

MIR135A1
TEKT2
OTOGL
ARHGAP15
C17orf97
VEGFB
PPM1D
AC092542.1
AC091167.4
AL450338.1
AL662795.2
ATXN10
CCDC57
GRAP2
AC113615.1
AC013733.2
RTBDN
AQP7P2
LINC00888
CLSTN3
AC135352.1
IL4
C12orf10
FO538757.1
CLIP3
EMC10
SMOC1
AC008764.6
TEPSIN
GOLGA8B
AC023355.1
AC005224.3
LINC02062
ACYP2
EXOG
STXBP5-AS1
CASP12
AC112250.2
RPL3P9
NDUFC2
CACNA1D
AC022211.2
AC011468.3
RYR3

SLC6A13
MRPL53
FGA
AL133216.2
CLDND2
CTSG
AC004148.2
ANGPTL1
AC104984.4
AL022313.4
AC009102.2
VPS51
AC120498.9
LRRC74A
AL359921.2
FAM71F1
RASAL3
LINC00368
AC027559.1
AC010632.1
FUT7
AC005332.6
NPRL2
CUTC
GIMAP8
LINC01993
CACNA1C-AS2
BOLA1
MT-ND1
GLUD1P2
CACNA1G-AS1
AGAP10P
FAM131C
SOAT2
AC091860.2
SAXO2
CTDP1
AL162574.1
CHRNA10
AC007638.2
RN7SL417P
GPR155
SLC37A4
AC092490.3

AP006248.3
MKNK1
SDHAF2
TMED11P
AC009061.2
ZHX3
AC141002.1
PTF1A
PDCD6IPP2
BX842568.4
HAUS1
ENDOG
FABP4
PNLDC1
RIMBP3C
AC104072.1
KLHDC1
IGHD
AC004023.1
TIGD3
AC010997.4
AC124657.1
RASGRP2
AC010624.1
PIAS4
AL137244.1
AP003110.1
MIR3936
SOCS1
AC120498.3
ELOF1
AC127024.2
LINC00847
RPL21P75
TECPR1
AC129492.5
AC104063.1
AC010624.2
WAC-AS1
AL451165.2
AC011444.3
UNC93B3
C12orf76
SENCR

NISCH
AL589739.1
P4HTM
LINC00864
LINC01637
AL645924.1
P2RY8
C22orf34
RPL10P19
RAB39A
AC068987.4
VKORC1
AC012313.6
PDZK1
AL591501.1
KNDC1
PM20D1
SLC5A9
AC092279.1
AL645998.1
TEX264
AC093462.1
MMP24
ZNF571-AS1
SAMD11
AC117457.1
AC079610.3
MFNG
BCRP3
AC107081.1
AC112243.1
HBZ
FAM124A
MBD3L1
AC138965.2
MTCO2P12
AL157366.1
SCGB2A1
AC093458.1
AP001596.1
AP001825.1
TDRG1
AC010890.1
AL035706.1

TUBA3FP
AC133540.1
SBSPON
LINC00563
CCDC175
ATXN7L2
RBM15-AS1
IGSF11
TRPM6
DKFZP434H168
EPOR
LINC01679
TDH
LINC01736
TCEAL6
ADAM29
LINC00951
MPV17L
LRRC8C-DT
RPS6KA6
AC034102.5
SNU13
AL139275.2
SMIM19
LAMA5-AS1
PALMD
CACNG2
AL731684.1
CCDC25
SMIM7
METTL23
MPPED2
AC009268.2
AC005593.1
AC010422.2
IFT27
SCRT2
SPINK9
AC107214.2
AC012513.1
IGFALS
TMEM108
RPS15
TTC28-AS1

PRLR
ALOX12-AS1
AL138690.1
FAM66C
EPB41L4A-AS1
CNTN5
FAM171A2
GAS1RR
NXF2B
DCX
LRRC73
FZD10-DT
TUT1
CXXC4-AS1
AC239585.2
DGCR6
AC104532.1
CNR1
AC099689.1
AC087343.1
RNU5B-4P
PHBP7
AC004597.1
SCN4A
FAM21FP
AL359918.2
FAM13C
KCNK12
COCH
AMACR
AC099684.2
PLGLB1
AL121782.1
AC009227.1
AL050331.1
AC005005.3
FAM209B
LINC00434
AL035587.1
RNU6-94P
AC093591.1
HRASLS
AC104561.1
AL008707.1

AL606760.1
GPS1
GMFG
NEURL1-AS1
AL355300.1
ZNF248
DHRS7B
B3GALT2
ARL2
DNLZ
FAM149A
TRPC4
AC024361.2
ADAMTS7P4
PLCXD3
AL161935.3
MYO3A
AL354809.1
ELP6
RIMS2
KLHL26
AC009041.2
MT-CO3
AL117339.4
C9orf163
RPL34-AS1
GPIHBP1
AC010536.3
NXPH4
AC078864.1
FAM95B1
LINC00158
AC023300.2
KLHL34
GRK3
AC005009.1
NANOGP1
TTC21A
AC012676.4
COX14
AF131216.3
AC103691.1
RTL9
CDS2

HCG17
AP002414.4
WDR5
TTC7B
MAG
C10orf62
PWAR1
AC062029.1
CYP1B1-AS1
LINC00515
AL353747.2
FAM229A
C17orf49
AL122010.1
KIAA1324L
MYBPH
ZMAT5
LINC00852
AL049838.1
AC016876.2
DDRGK1
CBY3
RDH12
MASP2
RELN
LINC01725
AC084116.1
KLHL7-DT
CTD-3080P12.3
NLK
MYL4
LINC01233
AL354824.1
SPEG
RYR2
AMY2B
PRAM1
CTRL
GLRB
LINC02388
STMN3
ITIH5
AC004882.1
FHIT

RPP25L
TCP11X2
CD79B
S1PR4
FBRSL1
LAGE3P1
AC035140.1
AP006289.1
AL157938.2
ZNF446
CCDC107
AP000866.4
AC008761.3
RPL9
RASA4B
AC005332.4
GRM1
PALM
ZNF493
CALCB
TMEM191C
CBR4
RPS9
SREBF2-AS1
CX3CR1
CCDC168
ZNF34
GFRA3
LINC00543
AC012213.4
GHR
AC123912.4
CMA1
AC083829.1
AMER2
AL022328.3
AL356489.1
CNRIP1
AC010883.1
AL157400.3
MRPL46
GOLGA8CP
RPL12P27
TMEM91

DPYSL5
DHRS4
PGM5-AS1
STPG3
AC090136.3
DDO
ADAM33
FSD1L
LINC01011
AL449403.1
TMEM203
AC104162.1
RNU7-171P
SMIM18
KRTAP12-1
AC079140.4
PDE1B
MORN4
TMEM11
TSPEAR
ZNF575
AC009159.3
MIR4740
LINC01544
CA10
ARMCX4
AC244517.5
AC046185.3
PRSS3P3
11-Mar
INCA1
URGCP-MRPS24
EFCAB12
AC005884.1
ZNF844
ZKSCAN2
CA8
AC004147.4
AC144652.1
CLSTN2-AS1
AC025754.2
AL390961.3
GOLGA8IP
AC009118.2

AC097641.1
CNPY2
LYRM2
ADAL
FAM47E
NCAM1-AS1
AL353803.2
TTC39C-AS1
LIMD1-AS1
MAGEL2
FREM1
ARMH1
RNU6-346P
SGTA
C15orf56
ADCYAP1
ECE2
AQP12B
DUSP28
AC011979.1
C5orf49
CD200
MTFR1L
KIRREL3
AC006504.1
TRBV7-1
ACR
AC243962.1
ZC2HC1C
COL23A1
AC125494.3
C6
RAPGEF4-AS1
AC004224.1
RNU6-460P
ZBTB46
HSPA1L
C2orf74
AC087482.1
PGPEP1
AC253536.6
C7orf50
LINC02150
AL109917.1

RAB24
PCBP1-AS1
AP003068.4
MICU3
CRYBB1
RHOXF1
MON1A
VWCE
TANGO2
DLG4
CD1D
PROZ
LDB2
AC007391.1
NUDT14
CASKIN1
CBLN4
EPB41L4A-DT
TOM1L2
AL513283.1
MIR4269
USP12-AS1
AC097641.2
AC068722.2
LINC02526
TUBG2
AC091304.2
MIR4458HG
MMP26
CLCNKB
TMEM161B-AS1
ATP8A2
AC004832.1
GRM7
RNU4-62P
FGGY
AC005586.1
AFF3
AC112204.1
AC027807.1
PWRN3
FLYWCH2
NSG1
Z97206.2

TOMM20L
SHLD1
SCRG1
AL512791.2
LUC7L
KBTBD6
AL049543.1
PYGM
AL121658.1
SPINK2
ENPP5
SYT14
CYSLTR2
AC055720.2
C1QBP
CLPP
AC097468.2
ESRRB
AL360295.1
AL603839.3
SCARNA20
RBPJL
AC124303.2
HAGLROS
EPOP
CACNA1G
GPSM1
TTPA
PEG3
MYEF2
LCMT2
WNT10B
MNX1
TTC25
TCEAL1
TRIM55
MSH5-SAPCD1
CYP4F25P
CACNA1C
GNG4
LINC01358
TMEM70
ID4
AC090241.2

AP002847.1
MYLK-AS1
AC107398.3
NDUFC1
TRY2P
TMX4
RN7SKP214
AC132872.2
KCNT2
ANKRD19P
AC144568.1
FAM19A4
TREML1
MYT1L
RNASEH2B
GLYCTK
AKAP6
AC005342.1
MTERF4
AL358216.1
PPFIA3
USP41
PSEN2
NDUFA3P4
AC022898.2
ALG9
AC010976.2
AC112693.1
AC137723.1
PRDX2
FER1L5
ERG
AC027281.1
RTL4
SLC7A8
VAT1L
ALLC
WASH7P
UBA52
FOXRED2
KIF17
AD000671.2
AC003973.3
LYVE1

KHK
SUPT4H1
SLCO4C1
FAXC
AC120498.1
AC015871.3
AC100803.1
SRGAP3
AC104971.1
NDST2
AC015982.1
WDR24
AC027307.3
AC091891.1
AL031055.1
GPHA2
C19orf18
CAMTA1-IT1
TPO
GPR119
C18orf65
NDUFB1
WDR13
AC024257.2
RBM6
AC055811.2
AC010240.3
AC005306.1
AC079210.1
LINC02532
SPATA1
MAP7D2
IQCN
H2AFVP1
MAP2K5
AC025048.4
C3orf22
AC119751.3
ERC2
FAM217A
GSTA2
GOLGA2P5
SPACA3
C10orf53

CLEC2L
AC005523.1
LINC00526
AL133342.1
GDF7
GPR17
C19orf24
IFT74-AS1
FAHD2A
RPL15
EIF3G
AL031658.1
RAB30-AS1
FAM43B
AC136604.3
AC010547.1
ADAMTS13
AC026333.4
HSPA4L
AC100814.1
AMBP
AC055717.3
C17orf75
FFAR2
AL590677.1
AC147067.2
AL109918.1
AC098591.1
AC011284.1
USP27X-AS1
SAMD3
ISYNA1
SLC17A4
AC079467.1
TMEM202-AS1
HID1-AS1
BTG2
AL391244.1
SIK3
AC005486.1
LINC01335
GOLGA8S
AC005856.1
RNA5SP111

RPL21P16
AC091181.1
AL136972.1
AF127936.2
AL672291.1
AC104232.1
LINC02217
AC063926.2
FBXW12
BCHE
FXR2
CFAP97D1
AC008915.2
AC123912.3
PHF7
LSM10
AL133419.1
AC012358.2
AL353803.3
LINC02251
CYB5A
KCNIP1
LRCOL1
TCTN1
FSD1
GABRB1
LINC02256
BCL6B
SENP3
CSGALNACT1
AL049775.1
MIR659
SEMA6B
NBEA
CELF6
C14orf178
AL590068.1
CCDC158
FAM27E3
AC004485.1
MRPL38
ANGPTL3
AL359710.1
AC006445.1

AC131159.2
AC107982.3
AC012184.3
PPP1R14A
GPAT2P2
ZNF266
DPT
Z92544.1
TRIM63
ASTN2
PARM1
LINC01624
AC016065.1
LMO1
NDUFAF4
AC011297.1
CACNA1I
NEIL1
SIVA1
AC069544.1
AP000997.3
AL592114.1
RPL7AP66
AL590426.2
AC005736.1
RPS23
IRX1
CLEC4G
AC008743.1
INKA2-AS1
TNKS2-AS1
AC131097.2
KCTD19
ROCR
TP53TG3D
OLIG1
AC145207.9
AKAP7
CR769776.2
LIPJ
CHL1-AS2
AC124312.4
MSX2P1
ZBED8

DCDC1
NKAPP1
10-Mar
PDILT
GPR75-ASB3
AC113403.1
AC016257.1
AC131235.4
CCDC181
LAMC3
AC124312.3
ZNF815P
AC117382.2
HMGA1P4
PARVG
CCDC127
NDUFV2-AS1
SDCBP2-AS1
NOL12
AC090152.1
TMA7
HAR1A
ZAP70
KANK1P1
AC110285.5
AL392089.1
AC092944.2
LINC01191
LYRM4
SFXN4
FKBP8
WNT9B
STXBP1
AP005131.4
AC117500.1
AC022296.3
AC012618.3
EDARADD
AC083843.3
MORF4L2-AS1
AL109945.1
AP000842.2
YES1P1
SUGP1

HELQ
AC135048.3
TTYH2
AP000926.1
LHFPL1
AC138356.1
AL049597.1
RNU6-1272P
RNU6-776P
ABHD10
BRD3OS
RNU6-703P
MATN4
LINC02139
LINC02273
NDST1-AS1
BX537318.1
SLITRK1
GPR27
AC012358.3
RBFA
KCNJ4
RPS26P21
ZFP82
AL354707.2
AC021188.1
ABCA3
AP005717.1
CHRDL2
RPL10P3
EMILIN3
ABCA10
UBL7
AC005197.1
AL137802.2
SIM1
AL356020.1
AC087741.1
AC097515.1
NOTCH4
AL132822.1
IGLCOR22-1
AC025165.5
CHRM5

AC068533.3
MT-TY
AC025211.1
CHCHD2P4
MIR578
RETREG3
ABHD1
RNU6-1189P
ADAD2
AL096870.2
NICN1
HMBOX1
ZBED5-AS1
AC008747.1
CUTALP
AL023581.2
AC005363.2
ARMCX1
AC091965.1
MAGED2
AC004656.1
ZNF135
NRN1
LINC01607
LIMD2
AC027117.2
CNTFR
AL133467.1
AC006116.1
CERKL
ZNF569
AC244090.2
GPR42
EBI3
MOAP1
SLC25A35
AL135786.2
RPSAP10
ABHD15
LINC00641
AL358472.1
TMEM74B
USF2
AC114728.1

LRRN2
SMAD9-IT1
AC023055.1
SAFB
LINC01754
TNFAIP8L2
AC011444.2
TEKT4
GPM6A
ZBTB49
MTND2P24
AC105402.1
TRBV7-4
GUCA1C
ELOCP28
AC239798.1
GLYCTK-AS1
NPAS4
ERP27
AC092118.2
KRT24
NPPC
FAM78A
SOCS2P2
AC005899.6
AL121768.1
GNPTG
RNU6-605P
BORCS7
LINC01783
AC109809.1
AC009779.2
AC053503.1
AP003419.3
AC008074.2
ZNF77
AL354877.1
ACOXL-AS1
PCCB
AL162311.1
AC034228.1
FAM186B
AC010624.4
CSMD3

SUOX
MIR663AHG
NRF1
LINC00305
GTPBP6
AC012313.3
AC013643.3
AC011468.5
UFSP1
ZCCHC12
SCAND2P
SOCS7
CTD-2297D10.2
MAGEE2
SLC26A4
PPM1F
SZT2-AS1
CCND2
PNMA6A
SCGB1C2
AC087741.2
GAB3
OR5K2
ADHFE1
AC110079.2
ISL1
RNU6-833P
ITM2A
ENTPD3
AL354696.2
GMPR
DLG5-AS1
LINC00235
ABCB1
RAP1GAP2
NPTX2
ANXA6
HCG20
AC015819.2
AC011899.1
PRIMA1
AP002765.1
RBFADN
JAKMIP3

HAP1
HNRNPA1P16
FRMPD1
AC010504.1
DM1-AS
TIMM44
AP006545.1
EEF1AKMT1
C17orf80
ZIK1
AL138831.1
AC018797.2
BVES-AS1
KCNC2
SNCA-AS1
CACNA1C-AS1
IL33
HRAT92
RN7SL121P
ELL2
AL157911.1
EPHA5-AS1
AC129492.4
AC093909.4
AC009955.4
KLHDC8A
AC074183.2
RASGRF1
AL357060.1
AL355990.2
DYM
CSRNP3
MAP2
ATP2B2
GPRC6A
RPS3AP44
ADAMTSL3
ATP5PO
ZNF541
POMGNT2
ZNF730
CNTN2
SLCO1C1
WDFY2

AC007671.1
SLC18A3
AL034397.3
OR5B2
SYPL2
AC007493.2
AL353795.2
CABP1
SPATA4
MRAP2
RPL12P25
AL449403.2
AC112503.2
FBLN7
LINC01785
FBXO16
LINC00574
SRGAP3-AS4
AC004754.1
CIRBP-AS1
NUP88
CHKB
AC087392.2
SEC14L6
STARD7-AS1
LINC00639
RPL17P22
AC037487.1
CPLX1
CROCC
RHOJ
LINC01786
AL022396.1
PRR5
AC090993.1
MTNR1B
CRYGEP
FLT3LG
ITFG2
LEFTY1
KCNQ2
SHISA8
ZNF536
LINC01412

AC107464.1
RPL7AP50
WDR7
RNU6-2
Z99774.1
TSEN54
MCF2L2
ZDHHC8P1
AL136141.1
LINC02015
MYMX
NAALAD2
ING3
DLK1
P2RX6
NUP210P3
TTLL10-AS1
HIST2H2AC
LINC01863
AC025811.1
AC099509.1
AC026469.1
RNU6-111P
RPL3P2
AL356512.1
AC007681.1
ZFR2
CUEDC2
PET100
FBXO44
ZBED9
DUS1L
AP000446.1
AC078880.3
SPAG6
AC015802.4
LINC00652
AL929410.2
RNF7P1
AL031056.1
TMEM266
AL359532.1
AP001790.1
ZNF786

AC109347.2
TTC39A-AS1
LINC01567
ABAT
SEC22B4P
ZNF132
AC106820.5
BDNF-AS
LINC01197
NEK8
RNU6-722P
OBSL1
POLN
LINC00240
AC079601.1
LINC01337
USP30
AC009686.2
SLC25A38
OR2H1
MMD2
AC090517.2
ESR1
DAO
CYB561D2
FOXN3-AS1
ZNF607
AC105137.2
TOB1-AS1
KRBA2
TAB1
RN7SL403P
AP001282.2
ALG5
AC005832.1
GIMAP5
CACNG5
RPL35
FAM189A2
RPL31P12
AL162713.1
RPL7P58
VPS16
HADHAP1

HERPUD1
PKD1P1
AC245452.1
DNAI1
DCAKD
RN7SL316P
ZSWIM2
AC007207.2
AL032819.2
AC113361.1
AC132942.1
AP000439.3
CDK2AP2P2
RNU6-1267P
AC022392.1
NEURL2
AC093677.2
RPL21P120
GLYATL1P4
ABCA8
GIMAP6
IGFBP2
CTDNEP1
OSR1
AC073389.3
AC012074.1
RN7SL693P
MT-ATP6
AC008759.2
AL355574.1
AC005180.1
DEXI
AC006063.1
NDN
AC243830.1
AC079779.2
HSF2
BTG1P1
AQP12A
DDX51
AF064858.1
Z99916.1
SORBS3
AC107890.1

AC092979.1
NDUFA8
MED31
AC012508.1
PWRN4
AC103952.1
PES1P2
AL589765.6
CREBL2
MT-ND6
MEIS1-AS2
VPS53
LINC02382
OR2T8
AC009119.2
MYLK2
AC132938.3
AL160313.1
LINC02082
SFRP5
ANKRD34C
IGLV2-28
AC087763.1
AP002812.1
MIR217HG
AC027020.2
AP2A2
AL157884.3
CKMT2-AS1
AC007993.2
IL21R-AS1
AC093772.1
MT-ND2
AC027097.1
AC007773.1
LINC01085
AC010615.2
CRYBA4
TSGA13
HAR1B
AC093311.1
AC117498.2
HCN4
NDUFAF1

CPO
UXT
PPP2R2D
GRAP
RPL21P90
RAB11B
AC008050.1
PCDHA2
GLUD1P3
AC004921.1
GAPDHP74
UBA6-AS1
AL034369.1
BCL2
AC012146.3
AC011899.3
AC069528.1
NDUFS8
HSD17B10
AL109741.1
ANKRD23
FRY-AS1
NKX6-2
AP003041.1
SGCA
ABHD14B
SEL1L2
N6AMT1
AC010627.1
GALNT17
AC021148.1
EIF3LP2
MAP3K7CL
ZNF684
CIDEB
ATP11AUN
VDAC2P3
CAPSL
KISS1R
FOXI2
MAMDC4
THAP3
MKRN7P
AL161621.1

ALOX12P1
ZNF385D
LINC00616
AFM
CNTN3
PHKA2-AS1
AC009108.3
LINC00982
ACTC1
GLYATL1
IGKV2-26
CATSPER3
HSD17B1P1
RN7SL526P
RPL3P7
AC023762.1
RPL3P12
NFIA-AS2
AC012414.5
CASTOR2
PTCRA
AC139100.1
IL18RAP
NUDT16L1
AC139100.2
AL122023.1
PIK3CD-AS1
RHBDL3
TMEM192
AC109454.4
C17orf58
AC021231.3
DGKE
RF00066
MMP24OS
AC061961.1
AC004069.1
AC112219.1
AGAP2
AP004247.2
AL355810.1
CFAP161
AC005410.2
GDAP1

DPF3
AC066612.2
STK32B
BRAFP1
PCARE
AC104596.1
UGDH-AS1
AL353135.1
AC092155.2
GRIK1-AS1
AL356740.2
ZNF382
CRB1
AC068313.1
AC040162.1
MIR342
HEPACAM
DGCR5
TSC22D1-AS1
AP003108.5
ADH6
AL158835.2
Z97987.1
SLC25A45
ALG2
C14orf132
AC126177.5
BOLA3-AS1
WDR25
AC008105.2
AC005009.2
ALKBH3
AC010422.1
CARMIL2
AC025165.4
SLC2A2
VAV3
AC092111.1
SCGB1D5P
AL356481.1
VASH1
GLRA3
TPTEP1
AL359962.2

AC105053.1
ANKLE1
CENPS
AC012513.3
DNAJC12
CRTAC1
AP003717.2
LINGO1
GNGT2
RXYLT1
AC120057.3
ZNF324B
AC104634.1
SLC35G6
ZNF517
NPTXR
AL354977.2
AC114498.1
LSINCT5
LRRC29
AC105383.1
AC007610.2
LINC02226
ZFX-AS1
TTC36
COLEC11
P2RX2
NXF1
RAB6B
AL450226.1
AL137779.1
ENKD1
ALDH3A2
AL158835.1
AL021396.1
TRIM67
NALCN-AS1
GGA1
SDR39U1
ZNF599
AC022098.3
AC093151.3
AC004805.1
AC024563.1

AMZ2
AL390816.2
AC093157.1
F2RL3
AP000229.1
FOXN4
SDAD1P1
RPL10P7
TUBG1P
AC010425.1
AC078909.2
LANCL2
CTSLP2
AC092171.4
RPL3P1
AC090246.1
DRICH1
AC012467.2
AC111182.1
GAGE10
MATN2
AL357054.4
SYT15
MMRN2
TMEM232
RN7SL386P
ELOA-AS1
AP001767.3
EPO
SNURF
RFNG
PROX2
AC239800.2
AL512303.1
PPP1R16B
SMAD9
AC011462.4
AC011899.2
FGF14-AS1
TRPC2
NXPE3
MZF1
RARRES2P1
SLIT3

AL353743.4
POPDC2
CDH5
GGTLC1
LILRA1
DYNLT3P1
LINGO2
DHH
LINC01933
INPP5J
AC253536.2
OR10G3
SPOUT1
AL513548.1
CLPSL2
ZCCHC3
SEC24B-AS1
SLC35F4
AC079140.2
AOC2
AL627171.1
AL451074.2
LINC00269
TMEM240
H1FNT
UROD
PLIN1
PRUNE2
MIPEPP3
CRYAB
AC005154.5
SERHL
KIAA0408
AP002001.1
AC005578.1
ZNF471
AC016642.1
AC090921.1
ZSCAN30
ZBTB11-AS1
AC093142.1
NMUR1
CRYBB2P1
AL049839.2

DCDC2
CTC1
Z94057.1
AC005046.1
HBB
SERF1B
SPECC1P1
CCDC65
AC011498.1
AL354811.1
SH3BP5-AS1
RASA4CP
AC245047.4
RSPH14
GULOP
DACH2
AL354694.1
SCGB1B2P
BANF2
AC009220.3
FAM122A
AC106028.5
ENPP2
SWI5
HSCB
AC024257.4
CAB39L
DEAF1
AP001267.2
AC010185.1
AP005131.3
CNMD
AF130417.1
BMPER
AC096887.2
GRIK3
AC125611.4
AL117344.1
PMPCA
AC141557.1
AC084876.2
EDNRB
SPATA24
ARRDC5

NRXN3
AP001625.1
WASH4P
GLYATL1P1
CLUH
AC079148.1
ZDHHC1
IPCEF1
HSPB8
ATP6V1E2
CHRNB3
TMEM249
ADAM7
AC093107.2
AL353616.2
TNXB
NOXRED1
CEACAM4
RPL21P93
ANO5
SSC4D
FAM215A
HPCAL4
AC123567.2
AP001636.1
AC007861.1
AC092296.1
NLGN4X
AC090772.4
AL356489.2
UPB1
BLOC1S5-TXNDC5
Z97192.3
DIS3L
SPDYE4
AC099681.3
AC013244.1
FAM162B
EEF1B2P6
RN7SL186P
AC024337.2
UBE2O
AC087855.1
AC016757.1

AL359399.1
FIGNL2
AC022146.2
WASH2P
NFATC1
KRTAP3-2
ARL15
AL117190.1
PITPNC1
PSTPIP1
NPEPL1
U73166.1
PCP2
SLC10A1
AC116563.1
AC073934.1
MYO15A
AC245884.9
AC108488.1
ZNF253
ASCL4
AC018521.6
AL133163.3
AC091946.2
AL078599.2
ARHGAP9
C10orf111
AC004839.1
KRTAP21-1
AC133552.3
AC012146.2
LSM7
RPL21P108
ATP6V0CP3
EEF1G
AL121932.1
WDR88
AL133320.1
GABBR1
AC008592.1
AC133963.2
AC092849.1
AL392023.1
MIR208B

AL353743.2
APOLD1
AL133371.2
WDR37
PWRN1
AC079741.1
AC084125.4
ADORA2A
SEPT7P9
RAB33A
AL445493.3
AC017015.2
ZPBP
CEP83-DT
EIF3J-DT
LINC01795
LINC01471
COMMD6
ADORA1
UGT2B4
TBL1X
LIAS
C19orf38
LINC01704
AC067930.3
AC004477.1
WHRN
RFPL2
NGF-AS1
CMTM5
TMEM89
AC021439.1
PHOSPHO1
GH1
TCAF2
SERPINI2
RPL9P7
NEXN-AS1
TRGC2
MYLPF
C8orf48
SEMA3F-AS1
MIR6859-2
AC008806.1

AC004836.1
AC011481.3
AL590556.1
MIRLET7D
ZKSCAN2-DT
NT5C1A
MAGI2
RPSAP31
HNF1A-AS1
DXO
AC016885.1
MRPL41
PDE9A
RPL35AP26
FDCSP
AC087273.2
ZNF737
AP001178.3
TRAPPC5
CTSLP1
C4orf50
CAPN6
TSPAN12
FASTK
AP002754.1
HCG25
TMEM129
AL139805.1
TTR
SLC25A36P1
AC107958.1
NTRK3
AL049830.1
WASHC1
AGER
GAPT
AL391497.1
PROP1
FIS1
AC009034.1
ZNF527
AC007349.4
GPR158-AS1
AL079301.1

ADAMTS8
CD22
ZNF618
AL357055.3
AC015911.7
ZNF420
C3orf14
AC051619.7
ARMC2
UQCRQ
GLIPR1L1
AC025423.3
CENPS-CORT
AC010326.2
LINC00379
HSPA12B
TCERG1L
AL162394.1
AL451069.3
CLN5
MYOC
GYPE
C1orf146
C4orf48
AL162385.1
PINX1
AC021915.2
RPL12P30
NMRK1
AC010336.5
ZFHX2
RPS28P7
AC040977.1
UQCC2
AP006248.2
AC073957.1
APLNR
AC005482.1
KLF14
AC006001.3
TRIM39
AC091045.1
AL512604.2
NKIRAS1

COLQ
GLP1R
LTB4R
AL008718.2
AL121834.1
MED12L
ARL6IP4
TSGA10IP
AL603840.1
RNU6-520P
KLF2P3
AC064807.1
NUBP2
AC016582.1
AL353803.1
PRODH2
SPSB4
AC243756.1
AC080188.1
PARD3-AS1
CBX8
AQP8
AC053503.5
SDR42E2
MACROD2-AS1
BACE1
B3GNT8
TUSC3
AL365361.1
KCNMA1-AS1
ACMSD
LINC01780
NSMCE3
AL590399.4
AC093278.1
AC018766.1
AL121758.1
LINC01482
AC006064.1
AC124016.1
LRIT3
AQP7P1
AC133041.1
AC006116.9

SIRT3
CORO7
TRPC7
AP000436.1
AC037441.1
CEACAM19
PNLIPRP2
TRIM3
AC120036.4
AC129492.6
LINC01237
TRDN
HYDIN
DPEP2
AC012313.10
CHST7
AL137009.1
RNVU1-6
AF106564.1
NXF5
AC011700.1
AL357500.1
AC138207.2
FAM166A
TACO1
MS4A4E
SMIM6
NEFM
GIPC3
HS3ST4
LINC00680
TTC19
AC109826.1
PPP3CB
LINC00211
PKNOX2-AS1
AC008892.1
ZNF556
TENM2
HABP4
JUND
LINC02128
B3GAT3
KRT8P28

AP001922.5
CCL19
MYL6B
LINC02325
AC010307.3
MSX1
FAAP100
AC068790.8
LIFR
AC005165.1
AC126768.2
TRIM73
AL136038.3
AC011816.2
ASGR1
AL353678.1
STAB2
AC019197.1
MRPL27
ACVRL1
AL357078.1
AC133919.2
SIGLEC11
BRICD5
EEF1A1P34
CYP4F62P
FGF14-IT1
AC069185.1
ARF4-AS1
AC104938.1
OMG
AC011481.1
MIR4290HG
TMEM199
RHOXF1P1
AP1B1
PIK3R2
TOX
ABCC9
NCAPH2
CEBPE
PRKACA
AC010894.5
GPER1

C6orf58
ARG1
ELP3
FBXL21
CSN1S1
MYH7
AC018648.1
AC007790.1
DOHH
AC145423.3
AC010336.2
AC004908.3
AL117192.1
LINC01105
Z84480.1
RTN4RL2
MT-TG
REG3G
RNF150
PDE6B
RN7SL310P
P2RX5
LMF1-AS1
TNFSF12
OR8T1P
LIMS2
HGD
ZNF287
MPND
ZSCAN26
FABP5P14
AC090515.1
RFX1
AC018467.1
COX7C
KRT10
AC005498.1
UQCR10
AC078960.1
ARHGAP44
AP000880.1
HNRNPA3P16
WRB
LINC02284

NACAD
NDUFV1
RCC1L
AL353746.1
EPB41L3
SLC22A31
RPS14
AC135178.2
PTENP1-AS
ARHGEF7-IT1
NFU1
AC005899.1
CCAR2
C15orf40
WFDC10A
FAM173A
PNLIPP1
GTF2IRD1P1
PRR36
AC011472.4
AL445307.1
IGLVIV-59
RNU5E-8P
ZNF442
AC090197.1
PRORSD1P
LINC01277
TTC9B
PTPN5
KANK1
FAM193B
NOL9
AC026254.2
TUBA8
IL12A-AS1
AC003084.1
AC092813.1
AC027601.2
AL138787.1
LINC00475
PPAN
PKD1
MAS1LP1
MIR4488

PVALEF
TRIR
XKR8
AC063979.2
ZZEF1
AC091180.1
GEMIN4
RPL21P11
TMC2
AC245060.6
AJAP1
NDUFS6
LINC01798
AC005703.4
PEAK3
TCAP
CELF2
HSPA8P11
S100A12
RNU4-39P
AL138752.2
FAM78B
SLC30A2
AL358074.1
LINC02084
ID2-AS1
IMPA1P1
2-Mar
AC067969.2
MDGA1
AC132192.2
PDCD2
GATB
PGBD4P3
AC078850.2
UNC5D
PODXL2
GPR4
IL13RA2
DOK3
AC020658.7
ARRDC4
CHRM4
ITIH1

AP003086.1
ERN1
FUNDC2P1
AC091167.5
HIST1H3A
CLDN34
MC3R
LINC02371
LINC01480
WDR18
AC084706.1
TEK
PIRT
TNFSF14
LINC01522
LINC01901
CHODL
CKB
AL450263.2
RPL39P38
PTGER4P2
RPSAP76
AC026336.1
HSP90AB6P
AC011389.1
FAM209A
NYAP1
LRFN3
AC090971.3
LPO
RNU6-892P
NDUFAF5
DMD
FREM2-AS1
DISP3
AC087683.1
ADAM11
NDUFA3
ZNF208
AHI1
AC138466.3
AL135785.1
AL672277.1
AC012442.2

MED28P8
AP000721.2
AC018845.1
RNU2-70P
ZNF663P
LRRC24
RUNDC3A-AS1
VSTM2B
LINC01004
MPC2
PLP1
AC018738.1
MRGPRF-AS1
LINC00266-1
AC131571.1
AC004947.1
SLC38A11
GPR88
RPL7AP34
AC106872.2
SCN7A
NXPH3
AC009245.1
CPEB3
UNC119B
RPL37P6
ARSE
THTPA
RPL24P8
FADS3
AL133343.1
AL031733.2
RAB40A
ADCK2
AC073508.1
AC243960.1
TERF2IP
AL592182.1
TMOD2
FEZF2
AP000221.1
VIT
FCMR
SNORD115-21

ACVR2B-AS1
CYP4F30P
ZNF793
CR381653.1
CCDC149
AC073896.5
AC110792.3
AC007656.1
C17orf98
AC022167.3
AL138976.2
AL031289.1
DPYS
TMEM268
AC009078.2
TRIM52
AL365184.1
CCDC130
AC018755.4
AL359541.1
RPL13AP25
AC020905.1
RPL19
AL158211.1
MRPS33
AC092335.1
AP002812.3
FAM216A
NLRP2B
ARHGEF9
AC137723.2
CLTRN
FIG4
CALN1
AC025580.3
TUSC2
AC079907.2
AC022726.1
CD79A
PHBP13
OLFM3
AC067945.3
AL157902.1
AC012557.1

AC073352.2
AL078601.2
ELAVL3
AL359233.1
GRIA4
CAPS2
RNU6-784P
RIMBP3
RPUSD2
CRHR2
FGF13
AP003465.1
CHID1
AC011840.2
FAM87A
AP000944.1
PIGL
NKX2-3
AC092171.5
SLA2
AP000350.5
KRTAP8-2P
ART4
FZD3
AC106872.4
SGCZ
GPR173
AC025284.1
CERS3-AS1
AL691515.1
SPATS1
CCNQ
OPRD1
AC073896.4
AC106820.2
ZNF790-AS1
Z98885.2
EIF4E1B
ARG2
TSPYL4
PRKCQ-AS1
AC007881.1
GALR1
AC015849.1

PCDH12
AC092803.2
AC139495.3
ZFAND4
TMPRSS12
KCTD6
BRINP1
KIAA1958
AC005899.2
CHD5
AL512625.2
SNORD113-3
AC025263.1
TTC34
PRR3
RPL5P10
AL731569.1
NDUFB6
HDGFL3
OAZ3
AC096558.1
LINC01251
TTLL1
PCDH8
HTR4
LINC00539
AL356740.3
SAP18P2
SNRNP35
MRPS7
CCDC183-AS1
AC132008.1
AF213884.3
AL024498.1
MORC2-AS1
TPT1
AC009145.4
IMMP2L
AL589987.1
ITGA8
CCDC154
AC002480.1
C10orf142
AC091564.5

DPP10
LINC01185
AC002546.1
TSGA10
STARD6
AL031598.1
CSDC2
AC046143.2
KC6
SHISAL2A
CCDC38
AC018992.1
TNFRSF4
MPL
RPL21P131
AL032821.1
AC021517.1
CBARP
LDOC1
AC004696.2
AC114808.2
KHDRBS2-OT
MYLKP1
BNIP3P27
SOX5
ZNF626
AP001554.1
PTN
ACTN3
MZT2B
AC002094.4
LRFN2
AL359076.1
CCDC184
KCND3-IT1
STAG3
ZNF672
DKFZP434A062
AC034231.1
AP001189.6
MS4A3
RABL6
AC024610.1
IAPP

LINC01619
AL365434.2
FCER2
CABCOCO1
ZNF492
AL445423.1
NAPB
AC092954.1
WNT6
ULK3
ZNF346
MAGED1
AL356488.1
AL139383.1
AC104117.5
AC090192.1
SRY
AC093673.2
AC051619.8
BNIP3P30
AC005355.2
AC105402.2
ENG
ARMC7
CCDC96
AC092384.3
AC144530.1
MTLN
AARD
IL13
ST6GALNAC3
AC119751.6
LDHBP1
AC092809.2
ERAS
GNAS-AS1
SEPT4
SLC17A7
GPANK1
TMEM160
AC008770.2
ATP5MC1
ARHGEF33
MIR3677

AL592429.1
AL080243.2
AC011603.1
AL137779.2
AL035250.2
DHPS
DNAJC6
LINC02399
FAM167A-AS1
SLC4A1
SLC22A7
MFAP1P1
ZNF589
AC233728.2
AC099850.1
AC135178.3
AC080013.3
AC104561.2
CD48
LINC00892
CCND2-AS1
LAT2
FLRT2
MANSC4
TBX1
LRFN5
AC034228.2
CRYGD
ZNRF4
AC068025.1
CDK20
MAP1B
ARHGAP4
AL591885.1
AC008969.1
AP005271.1
AC008906.2
AP002001.2
AC008758.6
ZNF436-AS1
AL136301.1
LAMTOR4
PTCH2
AC020594.1

NDRG2
AC136475.5
MTCO1P42
TEX53
TMEM132E
MRPS2
AF129075.2
AC022001.3
RNF175
STAB1
RRAGA
GLUD1P8
AL139294.1
FRZB
SST
CELP
AC012645.3
BTF3P14
LRP5L
AL353572.1
AC079015.1
AC022447.2
MAP4K1
TMEM52
AC002451.2
AL133245.1
LINC00552
C10orf71
AC009495.2
SMIM11A
AC138230.1
TMEM251
AC037487.3
AL162411.1
ATP5F1AP3
RPL7AP49
C9orf40
AC104758.1
GRPEL2-AS1
RNFT1-DT
AL392023.2
AC013275.1
HMGN1P5
STUM

TMEM225B
KRT33A
AC007390.1
AL079343.1
KCNA4
AC087477.5
C8B
XAB2
SYBU
ENO3
AL445070.1
EDN3
LIPC
CLVS1
AC011383.1
AC127459.2
MUC15
FAM205C
TCTE3
LINC01681
AC068987.3
AC020934.1
AF067845.1
DISP1
AL732314.6
AC098614.4
AL445433.1
ADRA2B
AC091948.1
SYNE1-AS1
AC092378.1
WEE2
AC129908.1
AL357054.2
ALMS1P1
ARHGAP19-SLIT1
AL162586.1
AC087045.2
AL591479.1
SLC9A9
PNLIPRP1
INMT
AL118508.2
AL035530.2

LEAP2
STPG2
AL590096.1
UCP2
AC097359.3
AC004982.1
COPS8P3
AADACL4
LINC02207
VMAC
POLL
CYP4F29P
AC018653.1
KLHL13
DNAJB5
APH1B
AC002480.2
KIAA0087
RNA5SP242
TMEM100
AL358473.1
AC124312.2
AC138649.2
LINC02557
AC037450.1
PMP2
LINC01975
AC110998.1
AC107214.1
ANKRD34C-AS1
AL451049.1
AL008628.1
LINC02054
AL158070.1
AQP3
AP001360.1
AP001505.1
COX6A2
AL450332.1
HHATL
AC244517.12
AC087164.1
CBY1
SLC30A8

DSCAM-IT1
AC105052.4
MIR1250
UBE2G2
SMARCA2
PTOV1
AC245100.8
RNGTTP1
AL121906.2
FOXD4
CLEC4F
AC025178.1
HDC
HCG9
SOX12
AC006329.2
DNAAF1
LRRD1
LINC02340
SCGB2B2
OR13C2
FAM20C
TRPC7-AS1
PECAM1
RPL23AP88
AC104793.1
HCP5B
CBR3-AS1
HYI
AGTR2
LINC02102
AL627230.7
NDUFA6
MIR541
RFX2
AL133406.3
RNA5SP248
ANO2
AC046195.1
LHCGR
SOX3
BRS3
AC135050.3
DNM1P47

LINC01899
ACSM5P1
EIF3KP1
LINC00582
MAFB
AC079779.3
AL031775.2
CTRC
AC093305.1
TAF5
AC136443.3
FGF9
AL390955.2
CNTNAP5
ADA2
LINC00533
SLC5A8
AC021321.1
TBL1Y
AC090796.1
LINC02158
ARMCX3-AS1
LINC00324
TSSC4
AC103809.1
AL391095.3
LPA
AC023830.2
SMPD2
AC027287.1
CLK4
LINC02482
PCDH19
SNORA2B
AC008758.2
PTP4A1P2
AC097478.2
ZNF30
AL096869.3
AC092590.1
AL008727.1
INSRR
RPSAP17
SAMM50

SLC35F3
AC008852.1
AC024060.1
METTL2B
CCDC40
AC105105.2
RF01970
HEXA
AC091180.2
SMIM9
THOP1
AC076966.1
AC009299.2
AC090844.2
TPH1
LINC02592
TULP3P1
TIAM1
AC104653.1
USP11
AC063977.2
Z93930.2
RAD51-AS1
AC010746.1
RNU6-534P
ZNF829
SCUBE1
AP1S2
RUBCNL
ZNF441
LINC01475
DLL1
DCUN1D2
AL121972.1
AL162274.1
NFATC2
AC004522.2
C1orf158
USP44
TMEM147-AS1
LINC02249
AC129492.1
AC018730.2
RPL7AP11

RPL23A
AP005131.2
AL137028.1
CDH4
HYPK
BMPR1B-DT
NOL4L-DT
RF02219
ZSWIM5
DUSP16
ZNHIT2
BET1P1
GTF3C5
AC073072.1
GUSBP11
FO393401.1
TRBV29-1
AC078850.1
MT3
AC117386.1
CCL2
UBE2U
DUSP8P5
MAN2A2
SHLD2P3
BNC2-AS1
ASCL1
CDK5R1
RARRES2P4
AC007349.3
AL355355.1
SFMBT2
NKAIN2
AL138826.1
CHCHD2P7
AC012213.1
AC021092.1
AP001547.1
AC105339.5
SLC27A3
TRIM60P14
TPI1P2
DIRAS2
PPP1R12C

AC108472.1
GPR6
SLC24A5
LINC00963
AC010598.1
ADCYAP1R1
AL049749.1
AC036111.1
AC012442.1
AC025431.1
GOLGA8K
VN2R19P
NCF4
LINC00992
ZNF859P
HCLS1
ZNF563
GPR179
PADI4
AP003080.1
SUMO2P17
COX11
OR5AH1P
AC013733.1
DOC2B
SSTR4
DIRC3-AS1
AC087463.4
AL353801.3
PROX1-AS1
IGHV3-22
BHLHA15
DNER
AP001107.8
AC010300.1
RPL36
PRR29-AS1
NAGPA
LINC01666
AC108475.1
AC022306.3
AIFM3
KCNG2
AL031316.1

AC091982.1
USH2A
AC005082.1
MIR4324
AP001257.1
OR2B11
RNA5SP47
AC061975.6
ZNF879
SCARNA11
AC069294.1
RNU2-38P
AC107208.1
RNU6-1136P
MAPRE2
TUBB1
GIMAP4
AC009654.1
BTD
CEBPD
AL136380.1
AC022167.2
IL1RL2
AC073878.1
GPBAR1
INPP5D
MMP21
AC006065.1
BX119904.2
MEOX1
PNRC1
GRM7-AS3
ZNF557
ZNF689
ANAPC15
AC020917.4
G6PC2
SYT9
AL513323.1
AC010655.1
AC104794.2
AC080080.1
SYNM
CHL1

AC073626.1
AC073842.1
CDKL3
PZP
MIR4754
RPS6KL1
AC104083.1
GLRA1
AL390026.1
JARID2-AS1
AC106052.1
AC010999.1
AP000344.1
LAGE3
FAM237A
AL353611.2
HAMP
AC079385.2
AC027419.2
AC000089.1
RPL30P5
CCDC39-AS1
METTL21EP
AC084809.2
LENG1
IMPG2
PPP1R10
RPL17P33
DENND6B
CACNA1C-IT2
TMEM42
TTLL6
RNU6-1022P
AC090527.3
NECAB3
AL512306.2
CBX3P4
CMKLR1
SAPCD1-AS1
AC246787.1
AF123462.1
AL139123.1
CORO1A
SPRYD4

WDR61
MTRNR2L6
AC105415.1
RPL9P32
AC084018.1
RPL23AP32
AC005324.5
BRWD1-AS1
KCNA3
LINC00886
NAPSB
GCNA
LINC00921
TRARG1
APOC2
KBTBD3
AC009878.1
KLHDC8B
CGRRF1
FGF12-AS3
CEL
AC009065.8
DPEP3
PEX6
FABP5P3
AC004816.2
TEX22
MIR4489
SH3RF3
PDX1
AL162386.2
COL2A1
NFIC
AP005901.2
NANOGP6
C19orf44
DNM1P51
RFPL4AL1
AL157791.2
NGF
SLC8A1-AS1
AC046136.1
A2MP1
TATDN2

CCDC92
TMC3
RBM24
AC016727.1
AL355581.1
RFPL1S
MIR8083
ZNF439
SDHDP1
ABCA11P
RPL6P30
TCL1A
RGL1
LINC02596
NRG1-IT1
AC010975.3
AL354771.1
AC004847.1
CITED2
AKAP3
SUB1P1
GATA2
MYH6
U62317.2
AC012464.3
RESP18
AC090510.2
PLA2G1B
REELD1
AC103591.3
TMEM25
RN7SL357P
ARMC6
TCTEX1D1
LYRM1
IGF1
AC021491.1
AP003355.2
AL365205.3
AL034376.1
BRSK1
AP004608.1
PDZD9
AC027097.2

RN7SL305P
AC099681.2
AMT
BMPR1AP2
DPY19L2P2
LINC00486
PCK1
CHST2
ZNF503
RLN3
CGNL1
MPO
CPN1
LRIT2
AJ003147.3
AC063919.1
LINC02405
AP001099.1
CACNA1S
PEX11G
RUFY3
AC093879.1
GLYAT
AC069213.3
OR2L13
RPL12P35
TRAF3IP3
PDGFD
RPL21P42
TMEM256-PLSCR3
RPL4
PTGER3
SLC36A4
AC092135.3
NLRP11
YPEL2
CPEB2-DT
AC004052.1
STRA6LP
AC105402.3
SPEF2
ABHD12
LINC01550
AP002371.1

AC007292.2
AC087239.1
GABRG1
CYBC1
AC010834.2
NOL8P1
TEX14
PRKRIP1
AC009955.1
DENND1A
AC024587.2
AL451069.2
PRAMEF12
RPS4X
MYO1F
AC011551.1
PRLHR
ZNF658B
ATP13A5
PLEKHJ1
TBX21
RASGEF1C
FAM86B3P
AC112493.1
AP001063.1
AL359091.2
PRKCB
LINC01230
MIS12
SUCLG1
NAT9
SMARCE1P6
TMEM259
TNNI3K
SYCN
AL049780.2
IL17B
FAM99A
TNFRSF17
DNAH10OS
CD6
LINC00598
ABCD2
ANKRD44

AC090525.1
KCNV2
AC011451.2
AL049780.3
AC008946.1
CCL23
PWRN2
GRIK4
SOGA3
SMAP2
AC007485.1
HMGB3P22
TRMT2A
LINC02033
AC015813.2
AL049840.4
RN7SL138P
AL033527.3
SLC8A1
CRYGS
IGSF1
KRTAP3-3
SLC35G2
DERL3
AC245014.1
UQCRHP1
AL157904.1
HCRT
RNU6ATAC20P
NDUFA5P6
LINC00926
RIC8B
TPRN
AC148476.1
LINC02322
PDE7B
ITGAD
C1QTNF9B
P2RY14
PIK3C3
AC113368.1
GPR83
NME3
HNRNPA1L2

PCED1B-AS1
MTHFD2P1
AC005552.1
DEPDC5
RB1-DT
AC096677.1
TPT1P4
AC234783.1
TFPI2
ABCA9
AC096920.1
AC026495.1
BTG1
KRTAP16-1
SIK2
AC092941.2
TDRD6
AC015540.1
BHLHE22
AL359715.2
AC007686.2
MGC27382
GIMAP3P
NPIPA1
VIP
CXorf40A
C1QL4
HMGB1P44
AC009806.1
AL157392.2
ATG16L2
LRRC37BP1
AP001999.1
AP003469.3
AL157702.2
LRRC63
AC139099.1
AL049610.1
RNU6-5P
ATP8A1
GPR18
KANSL1-AS1
SPAG8
F10-AS1

AL160314.2
AC104463.2
AC013417.1
AC004862.1
AP001781.1
AL078621.3
AC080038.1
AF064858.3
AC121764.1
RGS5
STAG3L4
GNG3
LGALS12
AC005837.3
CDRT4
LINC01950
ESRG
LBP
RNU6-759P
RNA5SP247
ESRRAP2
SPIN2B
C1orf162
ZNF30-AS1
GZMK
AC015689.1
CTC-338M12.4
ATP13A1
AC022148.1
AL513550.1
AC069061.2
AC012640.4
NES
IGHV5-78
FAM129C
AL031656.1
RBBP8P1
AC005920.4
GYG2
AARSD1
MYOM2
AC108738.1
LINC00700
SIT1

OIT3
POLR2J
LRRC70
AC018450.1
AC010280.2
HOMER2P1
AC012368.1
LINC01765
NUP210L
ASNSP1
MTND6P4
STXBP4
AC023824.6
LOXHD1
CROCC2
UBOX5-AS1
SEC61A2
RIMS4
MIR8071-2
AC005840.4
LINC00685
AL138724.1
NELL2
TRBV6-1
AC091685.2
AL359265.1
SEPT7-AS1
DSTNP1
KCNAB3
OR2W3
AL390816.1
APOF
COPS4
ZFP92
AC023310.4
PTOV1-AS2
PDIA2
AC068643.2
PDZD2
AL022311.1
ADH1A
TGFBR3L
LINC02367
AF064858.2

KTI12
PAPPA2
DMPK
TRBV28
ARL4AP3
RAD17P2
AP001347.1
AC005487.1
HTR1E
AC010624.3
ELAC2
AP005242.3
RPL34P31
GALNT18
AL139288.1
MAGED4B
AC137630.1
ERICH3
AP003108.1
SETP17
AC093424.1
AC034102.8
TPH2
AC140725.1
GPC3
AC005920.1
U47924.3
LINC02499
AC005089.1
PABPN1L
GOLGA8R
AL355338.1
EBF3
PLCD4
AC097468.3
PEX16
GABRA5
AL354977.1
AC116447.1
C22orf15
WAS
RNU6-1056P
NHLRC4
TRBV21-1

AL590648.3
AC145285.6
AL138827.1
AL392172.1
POU2AF1
OTOAP1
AC092667.1
AC055717.1
BAALC-AS2
AC129102.1
AL451064.1
AC087565.1
RPL7AP60
ATP5S
LAMB4
AC025594.2
HEMGN
TMEM17
AC015813.1
AL109936.2
SNORA36A
RNF157
CUZD1
AL023284.3
AP001476.1
AL606970.4
NAA60
AC136632.2
VN1R35P
AC048382.4
AC067942.1
HEATR9
AC034238.1
MUM1L1
INTS6L-AS1
GLT1D1
C13orf42
QSOX2
LINC00618
HSD17B1
AC011921.1
AMPD1
AC138150.1
SNORA12

	GNAS
	MT1M
	OPRL1
	RPS3AP22
	FOXD4L6
	STAG3L5P-PVRIG2P-PILRB
	AC069307.1
	PTRHD1
	GNG8
	C20orf27
	SNORA20B
	Z97192.2
	ATP6V1B2
	VPREB3
	AP003774.4
	AC007731.1
	CALB1
	OR8G5
	PTGES2
	PLD3
	BCL2L10
	ABCC6
	AC067751.1
	IGIP
	AL121578.2
	COX7A2P1
	AC079193.1
	KDR
	AC013270.1
	STAT5B
	RNU6-415P
	XCR1
	AC019129.2
	LINC02461
	AC079949.2
	DCAF12L2
	HMGA1P5
	SASH3
	RF00410
	FO704657.1
	AC010904.2
	AC000123.2
	AL031429.2

RN7SL313P
AC011365.2
FIZ1
KRTAP10-6
AL121983.1
ATXN8OS
TMEM132B
SLC25A51
TCL6
AC005041.1
TEX11
APBA3
AC025283.2
SOX2-OT
AC126118.1
GTF3C6
NCR3
AC008750.4
GEMIN8
RPL35AP3
DCLK1
AC046168.1
AC116036.1
PARP6
AL138916.1
MEGF11
ASB5
ABCG4
AP001189.4
AP002840.2
SPRYD3
YIPF7
AL589826.1
ACAP3
PLIN4
CPA2
AC015660.3
BPIFB4
AL353753.1
PRDM12
HAS1
AC092073.1
CSTP1
AC006023.2

PPIL2
AC110285.7
GYG1
UXT-AS1
AL133338.1
KCNIP2
RF00478
RAB6C
C5orf64-AS1
AC044860.3
PGPEP1L
RPS6P2
EEF1A2
AC011499.1
AL929472.1
AC036214.1
DRG2
AC087885.1
HIST1H2BA
CD5
AC097478.3
AC007387.1
CELA1
PSMB11
AC008267.5
LINC00861
ARHGAP33
ELFN1
MIR6851
ATOX1
KRT40
RPL23AP56
AC000036.1
MAP2K4
AL138820.1
AL365226.1
RNU6-20P
SATB1
AC012485.2
TRBV20OR9-2
TIGD6
AC006504.7
SULT1A2
GNL1

STBD1
RPL5P9
SSR4P1
AC011389.2
SLC16A9
AC112250.1
RPL18A
AL022722.1
MIR6757
AC023510.2
LINC01019
REXO5
ERVFRD-1
TRPM3
FP565260.6
SKP1P1
AC092354.2
LINC01558
AC026150.1
AC119403.2
RPS23P6
AL356317.1
IARS2P1
AP003027.1
AC017083.1
PAIP2
MIR6885
AP000777.3
TLR10
AC097658.2
TNK2-AS1
STK32C
RPS3AP47
RNF122
MYO18B
OR13A1
LINC01865
AL513477.1
PPY
ZNF511
PACRG-AS1
CELA3B
WWC2-AS1
RPS26P15

AC113935.1
LRRC77P
FCGR1CP
PSIP1
AC106897.1
AC026954.2
LINC02419
SMYD4
ASB2
FBXW5
MTMR9LP
ZIM2
RCVRN
AC006484.1
ANKRD39
AC006333.2
STAM-AS1
AC009166.1
POU2F2
FP475955.1
DOCK10
AC241585.2
AC096644.1
LINC02301
ZDHHC14
GACAT2
RABEPK
NLRC3
BNC1
AC244197.2
AC007834.1
AC073072.2
UBTF
AC016831.4
AC244157.2
AC103808.5
P3H3
LY9
AL021368.2
AC006116.4
MTG1
AC064836.2
MYBBP1A
AL021395.1

EFL1P1
AC007610.1
AC141273.2
AC073263.1
AL590764.1
MCAT
RNF138P1
AL162724.2
AC016526.3
AC025253.1
AC009501.1
MGAT1
ADAMTS10
SEPT3
AC140481.2
RPL34P34
AL160394.1
RNASEK
ZNF154
FBXO40
AC087762.2
AC006372.4
AP002387.1
AC107081.3
FAM124B
AC004980.1
CBX2
CEP131
LNCARSR
AL117336.2
AC000124.1
MUC19
SULT1A1
AC015911.2
AC011815.2
TRBV14
RPRM
HOXD-AS2
AC022730.4
MIR1268B
ZNF812P
PMF1-BGLAP
EPHA5
A1CF

PFN1P11
INPP5E
AC009812.4
BX072579.2
IMP3
GABPB1-AS1
SGK3
DNAJC27
AC009102.1
NSMCE4A
LTF
TBC1D17
RFPL4AP6
AC133435.1
FLG
DYNLRB2
AC090360.1
TCEA1P1
SCAMP4
AC091153.4
PARP4P3
ARMS2
CADM2
GJA5
AC133065.3
HOXD8
LRRC7
ITGA2B
FGD3
DBIL5P2
AC000403.1
SSNA1
AL137798.1
LGALS2
PTGFR
RBM5
CHIA
PLD5
AC020983.1
EGFL8
AC074389.2
MRPL12
LINC02417
CDH23

SFI1
MROH2B
AL606760.2
STX18-AS1
AL136090.1
AC125807.1
AC011498.6
AC022148.2
AC008267.1
PMCHL2
MIR4801
MAPT-IT1
FP325330.3
SLC17A2
GYPB
AC025034.1
BOD1
OR10AH1P
AL353747.3
ENOX1-AS2
RNU6-452P
CYSLTR1
RN7SL169P
AL109910.2
AC090531.1
PTPRM
ISCA1P6
AC016405.3
RGMA
AC097478.1
SPATA18
AC018892.3
AC087636.1
KCND1
AP000894.2
RPS26P6
AC091982.2
ZNF37A
PABPC1L2B
SYNDIG1L
SCGB3A1
DPYSL4
SMCO3
ZNF891

TBX2
AC026362.1
AC002400.1
RAD1P2
OR7E94P
FES
SLC25A47
RN7SL413P
PAPOLB
TRBV19
AC013549.1
AATK
AL590682.1
AC110995.1
THEMIS3P
IGSF23
AL391244.2
RPL7P56
INAFM1
MXRA7
NKG7
LINC00710
GAS5-AS1
LINC01060
TMEM235
HMGB1P35
AC073476.3
OR2L6P
ZNF547
TMEM130
AL139231.1
OR9N1P
SGCE
KRTAP10-13P
ENPP3
BTNL9
ZNF516
MTATP6P27
TMEM212-AS1
AC127455.1
TRAM2-AS1
NRAP
AC104984.1
RPL13A

GFRA4
FXYD4
WDR87
AC026951.1
SERPINA1
AC008972.2
HACL1
AL137246.2
AC005833.2
ADRA1D
CCDC177
RPL3P3
AL391095.1
AP000302.1
TRG-AS1
KIF9
ZNF708
NDST4
CARM1P1
SERAC1
TECRP1
CYS1
TP53TG1
NARF
CC2D2B
AC246785.2
AC090948.3
ACADS
SRRM2-AS1
AC113382.1
MIR129-2
AC010641.1
MAP1LC3C
GGTLC2
F2
MAP3K14
AC079760.1
ZNF37CP
AC013264.1
DMTN
MIR3667
ANAPC11
AC080100.1
AC074250.1

AC245884.8
MTND4LP30
CETN4P
SECISBP2
MIR300
AC063977.6
ZNF784
SNORD3B-2
CD40LG
AC069222.1
ASGR2
AC015813.7
SPATA20
RNU6-984P
NFKBIL1
IGHV1OR15-6
RPL32P34
AC010285.2
AC011298.1
EIF4HP2
DOK2
LINC02104
FMR1NB
LINC00327
AC079061.1
GRHPR
LINC01849
AC090515.2
TGFBR3
AC069234.4
ZNF14
SEMA4D
MCCD1
STARD9
HMGN5
NEGR1
SERPINA11
GALNT8
L3MBTL2
B9D2
HHEX
LINC01095
AC008592.4
SNHG26

NUTM2D
KCNH1
FGFBP2
ATP5MFP1
AC009690.2
SLC10A6
AL359711.2
DDN
AL357518.1
ATG4D
B4GALT7
KLRB1
AL355102.3
NPY
ALDH8A1
TM2D3
DOLK
EMC6
KLF1
HBM
AL353600.1
GPAM
MTCO3P39
AC005261.2
KLHL14
AC017100.1
GP1BA
HMGB3P10
BAIAP3
AC010271.2
ZNF470
NTN3
DNAH12
LINC00599
AC010329.1
LINC01659
MIB2
RPL9P18
AC079949.1
GPR26
RPL21P119
AC000111.1
GAP43
TMPRSS9

RPS17
AC124283.5
KRTAP5-5
AC126564.1
MIR106B
BTLA
AC145423.2
CFAP61
RPS3AP25
NBEAP1
RNU2-71P
CTRB2
SCN9A
ADIPOQ
FBLN5
CD37
FRMD4A
CICP27
LINC01410
FAM21EP
TUB-AS1
MTND5P12
LINC01266
FUNDC2P3
AC136475.1
AC103831.1
AP001922.6
CLEC10A
AP002986.1
SLAIN1
RN7SL732P
TOLLIP
ZNF561-AS1
PREX1
COX8A
CLECL1
ST8SIA5
CELA2A
AC245060.5
AL807752.1
BX470209.1
ZKSCAN7
AL078604.1
AC144831.1

P2RY12
RFPL4A
FAM174A
ATP9B
AC020637.1
TMC3-AS1
AP001880.1
RNU6-570P
MRPL58
CTD-2201I18.1
AC006369.2
RF00190
AC026202.3
HSD17B3
LINC02568
CCDC87
LINC01100
AL033519.1
DNASE1L2
AIPL1
FAM53B-AS1
RPL5P12
RPL5P25
MTCO1P9
AC019294.2
AC233702.6
BACH2
RAB6C-AS1
GFRA1
FBXO31
TRBV6-5
ATP5ME
IKZF4
RPRML
MSANTD1
AC107959.1
AP001043.1
RPL7P20
FLI1
STIM2
AC023051.1
OR13F1
RNA5SP67
MIR503HG

RNY1P11
TGM3
RPL26P37
AL137000.1
PGR
IFIT6P
MIR200CHG
BX284656.2
OXCT2
LINC00621
COMMD4
PDE6H
AC125603.1
CCDC22
SPOCK2
LINC01034
FLCN
AC074348.1
VAMP1
HNRNPDLP4
MED27
KRTAP12-6P
PRCP
LEP
ORAOV1P1
AC124856.1
AC120057.4
AL356981.1
TRGC1
AC027682.1
ICAM2
LINC02391
MSTN
AP005019.1
ZNF785
ATF4P3
DDN-AS1
AC093423.2
RNU6-324P
KLHL29
GALNT11
AC068726.1
PIANP
AC092447.5

AC087379.2
BEGAIN
C1orf141
DTX1
AC096915.1
VN1R91P
AC013403.2
SNORD114-1
AC008897.3
AL050305.1
PSMC5
MOB1B
UBE3D
AC104472.2
NDUFAF2
CLEC12B
S1PR2
MTND3P25
CUL9
NBPF20
KRT8P30
MAFA
GDF10
AC097505.1
AC104009.1
SCGB1D2
AC008738.1
AL021997.2
MIR7-2
BNIP3P16
HSPB2
LINC01320
AC095060.1
AC008267.3
WWOX
RNU1-67P
AC073389.1
CELA2B
COG1
SMG1P7
ZNF209P
ZNF788P
LINC01886
AL391822.1

AC011487.1
TNFRSF8
OLMALINC
AC005357.2
AC084855.1
A2M
TRUB2
AC003985.2
AC004969.1
RSPO4
AC087045.3
AL391845.1
AL022724.1
AC010205.1
AL158156.1
RSPH10B
MAATS1
AC093752.1
AC004067.1
C10orf90
AL031595.1
AC073834.1
ORM2
LINC00667
AC027237.2
NMD3P2
GSTA8P
AC007375.2
ARGFX
AC024559.2
AC018529.2
GAA
RPL11P5
AC138649.1
IGFBPL1
PROCA1
AL354824.2
CA5A
AL591167.1
AC245884.4
SCTR
LINC01140
CACNA1C-AS4
CNN2P3

AC008507.1
CCDC116
AL135818.1
SLC5A7
AC022893.1
NUDT11
AP003032.1
FAAP20
CFAP99
RN7SKP276
ASMTL
SSTR1
ZBTB40
AC025164.1
STK11
AC005740.4
AL021997.3
AC125618.1
NCDN
AC127502.1
AL031058.1
AC090617.6
AC025040.1
AC015921.1
CYYR1-AS1
LINC00348
RF00537
SNORD36C
ZNF189
AC026471.4
AL136526.1
AL500527.1
SIRPAP1
AC005014.3
ARSEP1
COG8
TKTL1
MS4A1
GCGR
AAMDC
CFHR1
LEFTY2
AP001628.1
FCRLA

AC098483.1
RGL3
KCNS2
C8orf89
MSS51
UGT2B15
AC233992.2
GADD45GIP1
AC080188.2
WHAMMP2
HP
AL606760.3
KLHL41
FGD1
AC016737.2
AC090607.5
STOML1
AC114776.1
AP006587.3
LINC01440
C8orf34-AS1
LINC02440
CYP27A1
MIR8071-1
MTCO1P22
DPP10-AS1
AL133492.1
AL353621.1
AL161756.1
AC015813.3
ANKRD20A8P
AC002094.5
AL390195.1
AL355353.1
ST8SIA1
NDUFS3
LINC01740
ZNF74
AC091198.1
AC244502.1
P2RY13
AC010201.2
CTRB1
AL159169.3

AL137026.1
MZB1
SLC22A16
AC084782.3
PCYOX1L
MYCBP2-AS1
FP325330.2
AC104137.1
RPS16P5
AC025259.3
RPL5P17
UPF3A
GUSBP3
AC006116.3
ADCY2
AL049651.2
9-Mar
AC012354.1
ALPL
TBC1D22A
TSKS
AC093330.1
LINC00184
AC010809.3
AL031602.1
URM1
AL365209.1
APTX
AC055713.1
TRBV6-4
RSL24D1P8
AL139246.5
LINC02028
C1orf194
AC022167.4
AC079340.2
FAM8A1
AC104971.3
AC025521.1
TEX41
AC087855.2
AL031864.2
PAWRP1
FSIP1

AC024933.1
ZNF85
RN7SL40P
AL049775.3
CROCCP2
MIR4539
LINC01735
TRBV7-7
AC123769.1
RNU5B-2P
AL135818.2
RN7SKP160
COMMD9
UBE2F
AC120036.2
CRB2
C12orf65
CNNM3
TMEM244
LINC02240
MICAL1
SNORA84
NEURL3
AC006504.8
SLC45A1
KCNE5
LRCH2
MYH1
CCDC183
AC023790.2
AC245052.1
ACOXL
RABAC1
IGHV1-45
AC233728.1
DPPA3P3
NPY1R
AL049712.1
RPL22P8
PPIAP70
OR10G4
FCGR1B
SCO1
HOXD3

AC006441.4
SLAMF1
AP006621.3
MTND6P22
AL603839.4
AC108156.1
P2RX6P
MTHFD2P6
CLSTN2
COL14A1
AC090616.6
AC117503.1
CLDN3
WDR81
UBE2J2
FOCAD
AL137060.1
COQ3
LINCMD1
AC109446.3
LINC01497
AC002545.1
AL136537.2
PRPH2
AC012065.3
LINC01118
CD244
MIR4477B
AC015909.5
AC068254.2
POLR2E
LINC01079
AP006621.5
AL162424.1
KCNH7
AADACL3
AC002428.2
MIR581
AC091078.1
RNF208
AL137186.2
IL2
CES1
AC109361.1

DLGAP1-AS3
DPY19L2P5
TRBV12-1
AC099794.2
AC022390.1
TMEM38B
AC109449.1
AL353705.1
TENT5A
LINC01198
LINC02214
HEPACAM2
CT55
PABPC5
HPSE2
SPDYE18
MTCO1P12
FAM19A1
MEGF8
AC005410.1
ZBTB8B
OGFOD1P1
SLC25A52
RNU2-63P
USP22
AC007557.4
SOWAHD
RNU6-378P
AL353194.1
LST1
SNRNP70
CD19
AC008267.7
MIR1273A
AC034114.2
AC018665.1
RERGL
IRF2BPL
AL031716.1
SRPX
AL136379.1
LINC02235
DISP2
AL031595.2

TSHB
GPX4
ACOT12
AC005280.1
AC024022.1
RIBC1
MARCKSL1P2
MT-ND4
ZNF286A
LINC02067
GIGYF1
GRIA1
MEIOB
LINC01904
PRSS3P4
KCNRG
TRBV5-5
ULK1
PEBP1P2
CTSLP4
AC009107.2
OSBP2
C3orf86
PPIAP55
AL359706.1
TPBGL
AC068896.1
BCAT2
UBXN10-AS1
GSTT2
FLYWCH1
LINC01857
KCNA1
BEST3
ACER1
SSUH2
AL513128.1
CDKN2C
AC004801.2
AC021491.4
AL354872.2
AL139184.1
AC008496.2
BLK

AL139125.1
AC244197.3
AC004771.1
AC090607.1
PGBD4
TNFRSF13C
FTH1P19
AL109811.4
AMH
AL589986.1
AC129507.4
IQCC
AC092326.2
AC078785.1
ITGAL
AC090970.2
SCARNA12
AC016152.1
AL121594.1
RORC
TMEM252
IGLV5-37
MIR151B
CNTN4-AS2
AL355796.1
GRM7-AS1
RN7SL205P
AP002358.2
AC122710.1
AC008481.2
PRH2
GP2
AL008635.1
TAB2-AS1
RPSAP57
RBFOX3
SNRPD2P1
AC091060.1
GABRA1
THAP8
PPIAP41
SNORD115-45
SLC25A20
MRPS14

HIGD1C
WEE2-AS1
AC097374.1
CDH8
GLRXP1
POLR1E
NOC2LP1
MEF2B
C1DP4
PCDH8P1
RPLP1
AL590399.5
DUXAP7
RBPMS2P1
OR52N5
AWAT2
LINC01352
AC099654.3
AC048337.1
MT1JP
AC092447.7
AC026336.2
MYT1L-AS1
AC006511.1
AC005906.1
PHKA2
AL121917.2
AC004918.1
Z83839.2
AC010245.2
ANKRD55
ASIP
AL391069.4
TMEM191A
AC008764.2
RNU6-529P
SPNS3
AC021016.2
TRPC6
AC092118.1
LRRC61
TAL2
AC009022.1
NOSIP

DLG3-AS1
NOSTRIN
KIAA0895L
AP004607.4
AL358937.1
AC084346.1
ARF5
C5orf47
AC087623.2
ECI1
AC024601.1
AC026191.1
GSTM5
ARSA
PPP3CC
FGF18
AC139792.1
MIR4453HG
AL133255.1
C4orf51
MIMT1
LINC02471
KLRK1
ASIC1
THRSP
MRPL55
AC005180.2
AC245884.2
AC068446.1
IGLV3-22
LINC01971
MIR148A
RNF227
PKD1L3
SLITRK5
AL592546.1
AL606469.1
C10orf105
RNU6-530P
AC087783.2
MIR6812
LINC02287
AC002540.1
BCO2

	CD7
	AP001972.3
	RPS23P8
	CLPS
	UCMA
	RCSD1
	AP005901.3
	ZNF24
	AC068506.1
	AC087741.3
	PLEKHB1
	AC004948.1
	SAGE1
	AL157392.3
	AP001324.3
	AC093390.1
	AC100793.2
	PPARGC1A
	RPL21P4
	LINC01970
	ZNF502
	AP3S2
	NCOA4P2
	HAO2
	DPF1
	IGLVI-56
	CPB1
	MT-CO1
	KCNC3
	TTC3P1
	MTND4LP5
	AC244453.2
	RNU6-287P
	JTB
	AC093775.1
	AC019257.1
	MIR5693
	AC006011.2
	TCEAL4
	AC099524.1
	AP000866.5
	MAGI2-AS1
	RPL23
	LINC02306

PABPC1L2A
WNT2B
AC104024.1
AC104561.3
IGKV2OR22-4
AC099343.2
Z97633.1
KRTAP7-1
NEFH
RBM10
ZNF876P
AL023653.1
AC074351.1
FBXO41
CCR7
MORC1
AP003072.5
FAM169A
FHL5
FAM187B
MIR6806
TRBV12-2
IGLV9-49
PDE3A
AP001007.3
IKZF1
ZNF350-AS1
PAK6
EEF1A1P33
CRYGFP
LRRC37A7P
TDRKH-AS1
AC015818.6
AC005208.1
AC023490.1
AL590099.1
AC132938.2
CASC17
AC024610.2
AL359924.1
AP001596.2
AC087516.2
REG3A
KRT8P15

AL161908.1
AL662860.1
ZNF620
AC239860.1
RNA5SP456
AC002553.2
AC025035.1
AC104794.1
AL391005.1
PNPLA6
C4B
KLRC2
CCKAR
ABCB4
CACFD1
NDUFA1
AC239367.2
AC012506.2
TMEM273
Z84468.1
OGN
AC025518.1
LINC01616
CPA1
LINC02317
GOLGA8DP
ARHGAP27P2
CNTD1
SNORA80D
AC012433.1
AC107294.3
WFIKKN2
MEF2C-AS1
ATP5PBP5
KRTAP21-2
LINC01954
AC015936.2
ZCWPW1
LINC02333
AC107027.3
AC110602.1
AC119751.1
RPSAP34
KCNIP3

NSUN5P2
PTPN7
MEIS1
KIR3DL1
AL449106.1
PTH1R
ATP5MC2
AC005303.1
GCH1
ADAMTS7P1
AD000671.3
ACTG1P10
AP002008.2
UROC1
AC084879.1
AC013644.1
AL513320.1
RAB36
GUCY1B1
AL132639.2
RPL3L
NTRK1
MTCO1P24
CER1
RRS1-AS1
AP001464.1
JAKMIP2-AS1
AC078852.2
FILIP1
ZSCAN5A
AC117395.1
OR10G6
AC093849.1
RNU6-987P
LINC01336
AL021155.1
LONP1
MIR4636
POMGNT1
AL157400.2
AC011754.1
GFAP
AC110716.2
MUSK

CNKSR3
PRDM5
BNIP3P9
AC093010.3
AL157396.1
LGI1
TNMD
IPPKP1
CEP126
AC243585.1
AC019171.1
AGFG2
AC107220.1
AC011495.1
SPEF1
AC007557.1
RPL13AP7
PQLC2
AL121990.1
AL512329.2
OSTCP5
AL117372.1
AMMECR1LP1
RIMBP3B
AC097103.2
HSPD1P10
UBXN7-AS1
AC007375.1
NMD3P1
LINC00648
TCAF2P1
AC007406.3
AC104090.1
CCDC196
AL133352.1
COA5
PITPNM2
RNA5SP78
EDA2R
AL592043.2
SERPINA3
AL161629.1
AL137849.1
CACNG1

ELP1
AP000550.2
ANKRD2
ASB9
MIR4538
MTIF3
RPL17P46
RHOBTB2
PAGE4
CREM
LINC00871
AC241377.2
EIF4BP3
REG1A
LRSAM1
AC005225.3
AL359821.1
PKN1
LINC02196
AL590233.1
EEF1A1P25
RPL37A
ZNF268
AC007731.3
AC117440.1
AL391069.2
SMCP
VPS33A
ZNF641
RF02126
AL160313.2
RN7SL181P
RPSAP11
CINP
NCBP2L
C14orf93
GRID1
BTBD2
PLEKHO1
LMF1
LINC02250
OR10Y1P
PIK3R3
AC006435.2

RPS20P5
AC137630.2
AC092068.2
WDR31
ADAMTSL1
AC034102.3
CDKL2
MCOLN2
AL627309.6
LCA5L
AC006487.1
AC103719.1
AC005225.1
AC138057.1
RPS5P3
BTF3P8
HRH3
NAALADL1
AC087273.1
CROCCP3
AC010680.1
FAM118A
EPB41L4B
AL136528.1
AC015813.4
MIR411
DHRS2
IFT43
AL450311.2
RPL17P3
MYOZ2
B3GALT6
NOS1
AC113349.2
SHANK1
DBX1
AC074131.1
IGKV2-30
PGLYRP2
HAVCR1P1
DNM3-IT1
AC005252.3
AC073133.2
AC126544.1

MIR587
AL359853.3
PAN2
AC105749.1
LINC02259
CHCHD6
CYB561A3
AC108451.1
BEST1
AL132765.2
LINC00528
ZP1
CDH20
RPL21P3
RPL35P1
AC011504.1
LINC01151
CSNK1A1L
GPC6-AS1
AC002094.3
C17orf82
LINC02096
CBX3P2
RNU1-94P
CNDP1
AC010619.2
AC008073.2
LINC01278
LINC01806
BHLHB9
HSPA12A
TMEM156
RPGR
NECAB1
AC064807.3
GS1-24F4.2
AL359644.1
DNPH1
AC027601.1
MAP2K2
AL354760.1
Z97205.2
REV3L
AF129075.1

AC016044.1
AC091153.2
DSCR8
REEP1
ELANE
LINC01787
C1QTNF9-AS1
FGF10-AS1
AC007823.1
AL118508.4
ADGRE1
CHCHD5
RPL23P2
MAST4
AL590240.2
AL118508.1
AC092920.1
FAM30A
TBCD
LINC01491
C1orf229
AP000866.1
VSTM2A
AC138331.1
CNP
AC108751.5
CD180
AC006019.2
AC012409.3
AP000911.1
AC024270.2
AL353622.1
DDX11L2
LY86
GALNT13
AC005332.7
AC006511.2
TEF
AC022211.4
RNU6-117P
LINC01871
AP001046.1
AL121694.1
MUSTN1

PDE3B
AC011773.4
AC016027.1
PSG8
DNAH6
ZBTB32
MTND5P11
ACCS
LINC00824
AC254562.2
SERPIND1
RIT2
HPX
AL591848.3
MIR6864
AP4S1
AC254562.1
Z73965.1
LINC02414
SPATC1L
CD1C
AL512625.3
ZBTB3
AC106892.1
AC007998.3
AC027117.1
DNM1
IGLV4-3
SLC7A10
PNLIP
AC005244.2
DLL3
AMY1B
SLC26A7
PRR4
PGS1
LINC02549
AC103858.1
ASIC2
BBIP1
BMP5
CELA3A
AC026471.5
AL355472.2

AC010900.2
PRMT2
GALR3
LINC00555
LINC02200
POMT2
AL109659.3
NUDT10
AC108047.1
AC126474.2
AC007402.1
TTC24
AC069503.1
AP000346.4
C11orf52
ZRANB2-AS1
AL139082.1
TRPM5
IGHD4-11
POLR2I
AC061975.1
AC090651.1
SIGLEC16
PTX3
AL512378.1
SULT4A1
TRBV11-3
EIF3F
CCL3
CLEC18A
AC024341.1
C3orf67-AS1
AC006270.1
RNU6-118P
AC025884.1
EFCAB6-AS1
AL133153.2
MSH4
TUBB4BP7
AL391219.1
ARHGEF40
RPL4P1
AC100823.1
ST3GAL6

APOE
CCDC42
RPL24
LINC01869
PRKAR1B
TIGD5
ZNF444
LINC01797
AL589993.1
AC021818.1
RPS3AP2
ZNF680P1
BHMT2
NME8
AC017002.5
AC091544.5
OR52N4
MTO1
AC090136.1
AL139156.2
MROH5
IGHD5-24
STK32A
ANTXRLP1
AC073257.2
AC055855.1
CHRM3-AS2
MYOT
CLEC17A
AC022634.2
THPO
HPR
AC010422.3
AL133372.3
RPS8
ECEL1
RF00001
NPM1P47
AC008556.1
FCRL2
BX255923.2
AL137071.1
YPEL1
IGHVII-60-1

ABCA2
TESPA1
AC104458.1
CDAN1
AL022324.3
AC114808.1
AGAP6
ATP4A
CD3D
TEX43
DEFB132
RPL7AP30
AL161909.1
AP001189.5
TSHZ1
GPR182
ANO3
AC096887.1
ZNF383
AC005838.2
RPL29P2
LRRC72
RPS2P52
PM20D2
IGLC4
CR382285.1
AC004865.2
SLC26A10
GPR101
AP002768.1
RNU6-1149P
RASL12
TSPYL1
RPL7P45
DBF4B
AC116351.1
RNA5SP301
KDM4E
AL583839.1
DPEP1
LRRC9
LINC01962
RHOH
AC092135.1

AL445228.2
MED22
TYSND1
DDC
ACOT13
MIR146B
GABARAPL1
AC135731.1
LINC00174
LINC01924
EID1
RN7SL583P
LINC01532
AC008105.1
AC013437.1
PARD6G-AS1
LRP1B
PPP1R32
LINC01176
AC026780.1
MECP2
AL158211.3
AC009133.3
FAM90A1
IFNA20P
MTDHP4
CD27
AC005300.1
BX255925.1
RPS27AP3
LINC02339
LINC01678
ZNF454
AC108474.1
ETFBKMT
AC138393.2
AC090957.1
SNORA47
AL121718.1
GLDC
AC099542.2
LINC01122
AC008708.1
AL354718.3

ELL2P1
SDAD1P4
LILRA5
AC024075.3
HES6
AC021739.2
IGLV1-50
MT-TD
PLSCR2
AL158211.4
AL731563.3
AC002090.1
IRX6
DUX4L50
HNRNPA1P34
CRH
AC107294.2
AL355864.2
ZNF512
AC138696.2
ADGRL4
UNC45B
LTA
AL122008.4
MIR7158
FCRL3
AL355974.2
AC023886.1
AL356490.1
AL512631.1
AC010422.6
LINC00332
C2orf27B
MIR491
LIM2
ADAM20P2
DAB1
MRPL43
POLR3KP2
RPS24P16
ABHD17AP5
AC105271.1
MIR635
SNX18P14

AC108748.1
PRTN3
MAGEC3
AL109659.2
AC112484.3
ACTG1P24
AL161457.1
RXRB
AC003982.1
CALR3
MORF4L1P4
OXT
AC068389.2
TSPAN32
EGF
CDH12
AC012507.2
KLHL1
AC079922.1
ATF4
AL162393.1
SNORD109B
AP000936.1
AC092431.1
AC079906.1
TRAF1
PHF1
MIR1179
MKI67P1
GATD3B
MINDY4
ITIH4-AS1
HDGFL2
AC006946.3
GNL2P1
AC108471.2
AC235565.1
TMEM187
RNU6-178P
RPS17P5
AL136295.4
AC012486.1
LINC01658
AC097487.1

AP003171.1
AC005996.1
AP001120.1
BPIFB2
NPFFR2
PPOX
PLAC9P1
MORN5
UBE2E2-AS1
AC011131.1
AC244021.1
ACSM3
AC053503.2
MIR216A
AL159169.2
RPL5P4
FOXP4-AS1
IGSF21
AC103564.1
EEF1A1P31
TMC8
RNA5SP515
IGLV3-4
BX119904.3
AC138647.1
AC012435.1
HAPLN2
AC005344.1
RBM17P3
AL512310.1
LINC01489
AC010547.4
AC107294.1
TRBV27
RNU6-1089P
AL096803.2
SMIM26
AC004801.5
TAS2R8
ZNF32-AS1
ATP5MG
AC005674.1
AL132838.1
AL158209.1

AC092471.1
SNORD116-2
GOLGA8O
HSPA8P13
Z82188.1
AC027130.1
SDHAF4
C16orf78
ENDOV
TRAJ5
TRGV4
AC005094.1
GATA1
PLA2G2D
AC091962.1
AC007969.1
IGKV2-18
AOAH
MTATP6P3
FAM90A2P
VNN3
HIST1H2AA
MIR4452
LINC01744
SLC25A34-AS1
AC010463.3
AC106872.5
ANKRD13B
RING1
AL078621.2
TNPO1P1
AL445465.1
AK1
CCNQP3
ZSCAN5DP
RFPL1
ERVH-1
PTPRZ1
FRMPD4
SNORD114-28
RGCC
AL139260.1
LINC02153
PTGDR

C11orf21
DUSP8
IGKV2D-28
RPL7P6
REG1CP
AIF1L
TMEM116
ONECUT1
RN7SL672P
SMCO4P1
IGFBP7-AS1
AC009517.1
RNA5SP442
FBXO4
ZBTB9
AC010654.1
DGCR10
AC012213.2
NDRG4
LINC02046
DCAF8
AC136475.9
AC018809.1
IGKV1D-22
TMEM115
AC008764.8
RPL5P30
AL139021.1
AP006545.2
HEXA-AS1
RNU6ATAC35P
SRRM4

SPRINGER NATURE
Author Services

Editing Certificate

This document certifies that the manuscript

Expression and Prognosis Analyses of Insulin-like Growth Factor 2 mRNA Binding Protein Family in Human Pancreatic Cancer

prepared by the authors

Xiao-Han Cui, Shu-Yi Hu, Chun-Fu Zhu, Xi-Hu Qin

was edited for proper English language, grammar, punctuation, spelling, and overall style by one or more of the highly qualified native English speaking editors at SNAS.

This certificate was issued on **October 6, 2020** and may be verified on the SNAS website using the verification code **2AEF-7E50-EF8A-ECFD-DACB**.

Neither the research content nor the authors' intentions were altered in any way during the editing process. Documents receiving this certification should be English-ready for publication; however, the author has the ability to accept or reject our suggestions and changes. To verify the final SNAS edited version, please visit our verification page at secure.authorservices.springernature.com/certificate/verify.
If you have any questions or concerns about this edited document, please contact SNAS at support@as.springernature.com.

SNAS provides a range of editing, translation, and manuscript services for researchers and publishers around the world.
For more information about our company, services, and partner discounts, please visit authorservices.springernature.com.
